# 1,4-Oxazepan-7-one trifluoroacetate: a modular monomer precursor for the synthesis of functional and biodegradable poly(amino esters)†

**DOI:** 10.1039/d5py00522a

**Published:** 2025-07-03

**Authors:** Tino Mackiol, Chloé Pascouau, Manuel Nagel, Tamara M. Bizmark, Luca Montesel, Jochen Fischer-Schuch, Pol Besenius

**Affiliations:** a Department of Chemistry, Johannes Gutenberg-University Mainz Duesbergweg 10-14 55128 Mainz Germany besenius@uni-mainz.de; b Institut für Biotechnologie und Wirkstoff-Forschung gGmbH Hanns-Dieter-Hüsch-Weg 17 55128 Mainz Germany

## Abstract

*N*-Acylated poly(amino esters) (PAEs) synthesized *via* organocatalytic ring-opening polymerization (ROP) offer potential for tailored, functional and degradable polymers. In this study, a universal monomer precursor toward *N*-acylated-1,4-oxazepan-7-ones (OxP)s was synthesized using a three-step approach, allowing for the introduction of various functional groups. Two novel oxidation sensitive OxP monomers bearing a double bond and a sulfide group were designed, as well as two monomers with alkyl moieties. The organocatalytic ROP of the OxP monomers using 1,8-diazabicyclo[5.4.0]undec-7-en (DBU) and 1-(3,5-bis(trifluoromethyl)phenyl)-3-cyclohexyl thiourea (TU) as catalysts was investigated. Polymerizations were performed under ambient temperature, affording homopolymers with narrow dispersities (*Ð* = 1.09–1.13). As a proof of concept, a post-polymerization thiol–ene functionalization of the allyl functional PAE was performed *via* photo-rheology experiments. Finally, the (bio)degradability of the *N*-acylated poly(amino esters) was evaluated through a series of degradation studies under mild enzymatic catalysis, in neutral phosphate-buffered saline solution and under accelerated conditions.

## Abstract 2

*N*-Acylated poly(amino esters) (PAEs) synthesized *via* organocatalytic ring-opening polymerization (ROP) offer potential for tailored, functional and degradable polymers.

## Graphical abstract

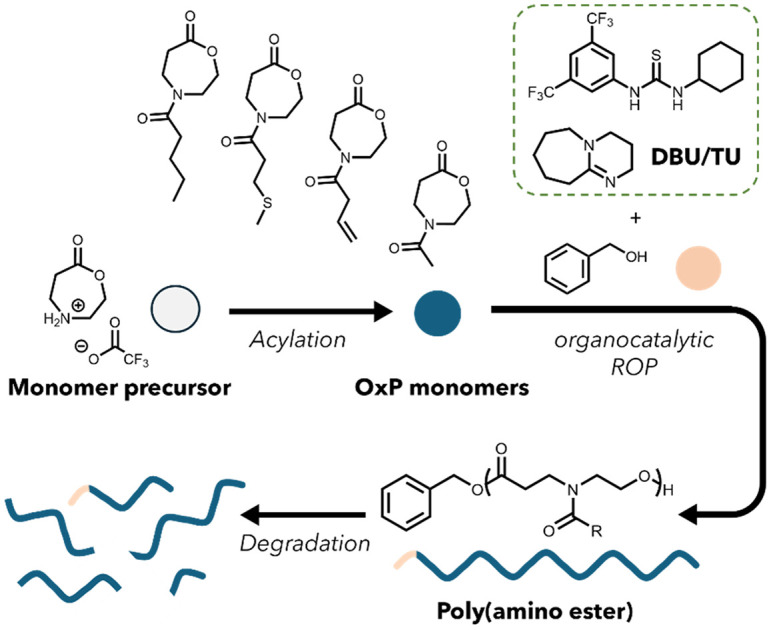

## Introduction

In response to the increasing environmental challenge, degradable polymers, such as aliphatic polyesters or polycarbonates, represent a class of materials that offer a sustainable solution to low-degradable polymers.^1,2^ These materials can be broken down into non-toxic components over time through hydrolytic and enzymatic degradation mechanisms, making them more eco-friendly and potentially more sustainable.^3–10^ Poly(amino ester)s (PAEs) represent a class of polymers, with a backbone of repeating units featuring degradable ester bonds along with amine or amide derivatives, enabling the incorporation of various pendant functional side chains.^11–16^ PEAs are used in biomedical applications as drug or gene delivery vehicles and as materials for wound dressings.^17–22^ They were found to be non-toxic for cells and their degradability was reported.^19,23–25^ In addition, the range of applications for these materials can be expanded due to their diverse functionalities and potential for post-modifications, such as crosslinking, coupling, and redox reactions.^26–30^ PAEs with various pendant groups are widely accessible through step growth polymerization techniques, including polyaddition or polycondenation.^11,31–34^ Surprisingly, chain-growth polymerization methods such as ring-opening polymerization (ROP) for PAEs are less common.^14,35–38^ Organocatalytic ROP provides a metal-free and well-established method for the synthesis of well-defined polyesters and polycarbonates under mild reaction conditions.^14,35,39–43^

Wang and Hadjichristidis reported the organocatalytic ROP of the azacaprolactone *N*-acylated-1,4-oxazepan-7-one (OxP) with a variation of aryl and alkyl substituents.^14^ The resulting poly(1,4-oxazepan-7-one)s (POxP)s were synthesized with controlled molar masses and narrow dispersities. The water-soluble derivative *N*-acetyl-1,4-oxazepan-7-one polymer P(OxP_Me_) showed very slow degradation in phosphate-buffered saline (PBS) solution and was suggested as alternative to non-biodegradable poly(oxazolines). The current literature reported synthetic route and Bayer-Villiger reaction in the final step of the monomer synthesis limits the preparation of a more diverse set of functional OxP monomers other than aryl and alkyl bearing side chains, due to the lability of many functional groups under oxidative conditions. Instead, we hereby report a broadly applicable synthetic approach for the synthesis of a wide range of OxP monomers using 1,4-oxazepan-7-one trifluoroacetate salt (OxP_TFA_) as a precursor. OxP_TFA_ enables the incorporation of various pendant groups through acylation in the final synthetic step. The synthesis of OxP monomers and their homopolymerization using the DBU/TU organocatalytic system are presented. The reactivity of the different monomers is evaluated by kinetic experiments of the organocatalytic polymerizations, which are analyzed by nuclear magnetic resonance (NMR), size exclusion chromatography (SEC). The (bio)degradability of the water-soluble P(OxP_Me_) with an acetylated side chain is investigated under mild enzymatic catalysis, in neutral phosphate-buffered saline solution and under accelerated conditions. Finally, a photo-rheology experiment is conducted to study gel formation *via* thiol–ene cross-linking using the allyl pendant functionalities of PAEs.

## Experimental section

### Materials

All reagents and solvents were purchased from commercial suppliers and used as received, unless stated otherwise. Dry solvents were obtained from Thermo Fisher Scientific Inc. Deuterated solvents were obtained from Deutero GmbH. 1,8-Diazabicyclo[5.4.0]undec-7-en (DBU) was purified by stirring over CaH_2_ for 1 hour followed by distillation under reduced pressure and was then stored in the glovebox for use. All operations involving air- and moisture-sensitive chemicals and materials were carried out in flame-dried glassware under argon atmosphere using Schlenk technique or in an argon-filled glovebox (MBraun UNILAB, <0.1 ppm of O_2_ and <0.1 ppm of H_2_O).

### Instrumentation

#### Nuclear magnetic resonance (NMR)

All NMR experiments (^1^H, ^13^C, ^1^H–^13^C HSQC, ^1^H–^13^C HMBC and ^1^H–^1^H COSY) were performed on a Bruker Avance II 400 or Avance III HD 300 spectrometer. The measurements were carried out at 274 K in deuterated solvents. Chemical shifts are reported in parts per million relative to the residual protons of the internal standard.

#### Size exclusion chromatography (SEC)

SEC measurements in THF were performed using an Agilent 1100 Series SEC system equipped with a SDV column set (10^3^/10^5^/10^6^ Å porosity) from Polymer Standard Service GmbH, RI and UV (254 nm) detectors. THF was used as the mobile phase at a flow rate of 1 mL min^−1^ at 25 °C. Toluene was used as a reference for the baseline. Poly(methyl methacrylate) standards from Polymer Standard Service GmbH were used for calibration. SEC measurements in DMF were performed using an Agilent 1100 Series SEC system equipped with a HEMA column set (300/100/40 Å), RI and UV (254 nm) detectors. DMF containing 1 g mL^−1^ lithium bromide was used as the mobile phase at a flow rate of 1 mL min^−1^ at 50 °C. Toluene was used as a reference for the baseline. Poly(methyl methacrylate) standards from Polymer Standard Service GmbH were used for calibration.

#### Differential scanning calorimetry (DSC)

Thermal analyses were conducted using a DSC 250 from TA instruments with an RCS 90 compressor, calibrated with an indium and *n*-octane standard. A minimum of 5 mg of polymer was weighed in a pan and subsequently sealed. All measurements were performed under a nitrogen atmosphere. Two heating and one cooling cycles were performed. To remove any thermal history, the samples were heated before the analysis, using a ramp of 10 °C min^−1^ to 120 °C. The measurements were conducted in a temperature range from −90 °C to 120 °C with a heating rate of 10 °C min^−1^. The glass transition temperature (*T*_g_) values were determined from the second heating cycle.

#### Mass spectrometry (MS)

Characterization of small molecules was performed on an Agilent 6545 QTOF-MS using electrospray ionization (ESI). Polymers were characterized by matrix-assisted laser desorption ionization time-of-flight mass spectrometry (MALDI-ToF MS) using a Bruker autoflex maX MALDI-TOF MS/MS with *trans*-2-(3-(4-*tert*-butylphenyl)-2-methyl-2-propenyliden)malononitrile (DCTB) as the matrix in chloroform and potassium trifluoroacetate as the ionizing agent.

### Synthetic procedures

#### Four-step synthetic route toward 4-acylated-1,4-oxazepan-7-ones (OxP)s starting from 4-piperidone

##### Synthesis of 1-tert-butoxycarbonyl-4-piperidone (P_Boc_)

4-Piperidone monohydrate hydrochloride (20.0 g, 0.13 mol, 1.0 eq.) and K_2_CO_3_ (21.6 g, 0.16 mol, 1.2 eq.) were dissolved in water (150 mL). Boc_2_O (31.3 g, 0.14 mmol, 1.1 eq.) was dissolved in THF (50 mL) and added dropwise to the aqueous solution under stirring at ambient temperature. After 16 h, the yellow solution was extracted three times with Et_2_O. The combined organic layers were dried over Na_2_SO_4_, filtered and the solvent removed under reduced pressure to obtain a colorless solid (quantitative yield).

##### Bayer-Villiger ring-expansion of P_Boc_ (Fig. 1, [2])

*m*CPBA (43.8 g, 0.20 mol, 1.5 eq.) was dissolved in DCM (200 mL). P_Boc_ (25.9 g, 0.13 mol, 1.0 eq.) dissolved in DCM (50 mL) was added dropwise while cooling. After complete addition, and the reaction stirred at room temperature for 16 h. The formed colorless precipitation was removed and a second portion of *m*CPBA (9.0 g, 0.04 mol, 0.3 eq.) was added to the reaction solution. After stirring at room temperature for 16 h, the formed colorless precipitate was removed. Saturated sodium thiosulfate solution was added, and the mixture was stirred for 30 min. The organic layer was separated, washed with saturated NaHCO_3_ solution and water, dried over Na_2_SO_4_ and filtered. After removing the solvent under reduced pressure, the crude product was obtained as colorless, slightly yellow solid. Purification by silica gel chromatography (cyclohexane/ethyl acetate = 1/1) yields the desired 4-*tert*-butoxycarbonyl-1,4-oxazepan-7-one (OxP_Boc_) (yield = 64%).

**Fig. 1:**
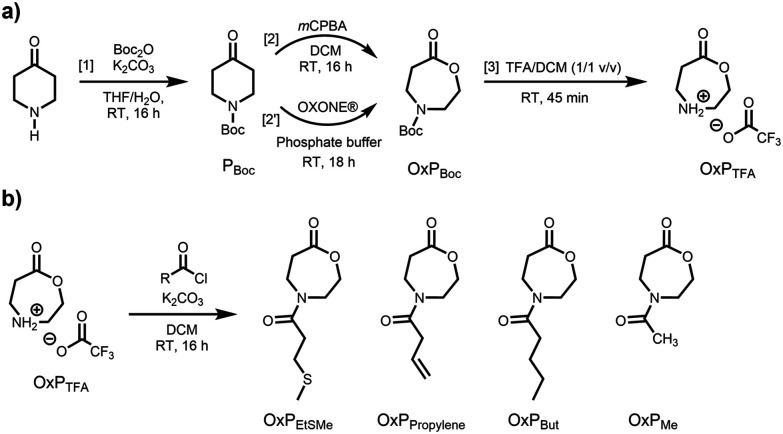
(a) Three-step synthetic route toward 1,4-oxazepan-7-one trifluoroacetate (OxP_TFA_) starting from 4-piperidone. [1] Boc protection, quantitative yield; [2] Bayer-Villiger ring-expansion, 64%; [2′] oxidation with OXONE®, 80%; [3] Boc-deprotection; 95%. (b) One-step acylation of OxP_TFA_ for the synthesis of four *N*-acylated-1,4-oxazepan-7-one (OxP) monomers with different side chains and functional groups.

##### Oxidation of P_Boc_ with OXONE® (Fig. 1, [2′])

P_Boc_ (500 mg, 2.51 mmol, 1.0 eq.) and OXONE® (6066 mg, 17.87 mmol, 7.0 eq.) were dissolved in degassed 2 M phosphate buffer (50 mL, pH = 7). The reaction was stirred for 18 h at room temperature under an argon atmosphere. The solution was extracted with ethyl acetate. The combined organic layers were dried over Na_2_SO_4_. The solvent was removed under reduced pressure to obtain OxP_Boc_ as a colorless solid (yield = 80%).

##### Boc-deprotection of OxP_Boc_

A solution of TFA in DCM (50% v/v, 25 mL) and OxP_Boc_ (5.0 g, 0.02 mol, 1.0 eq.) were stirred for 45 min at room temperature. The solvent was evaporated under reduced pressure, yielding a yellow oil. Suspending in DCM and precipitating twice in cold Et_2_O gives a colorless solid. The supernatant was removed, and the solid was dried under reduced pressure to obtain the 1,4-oxazepan-7-one trifluoroacetate salt (OxP_TFA_) (yield = 95%).

##### Acylation of OxP_TFA_

As an example of the acylation of OxP_TFA_, we provide the experimental details for the synthesis of OxP_Me_, while all other monomer syntheses and full characterization details are provided in the ESI:†OxP_TFA_ (2.0 g, 8.73 mmol, 1.0 eq.) and K_2_CO_3_ (3.6 g, 26.18 mmol, 3.0 eq.) were stirred in DCM (40 mL) at room temperature under an argon atmosphere. Acetyl chloride (1.4 g, 17.46 mmol, 2.0 eq.) was added, and the reaction was stirred for 16 h. The heterogeneous mixture was filtered, and the solvent was removed under reduced pressure to obtain the crude material. The crude material was purified by silica gel chromatography (DCM/methanol = 20/1) (yield = 58%).

#### General procedure for the homopolymerization of *N*-acylated-1,4-oxazepan-7-ones (OxP)s

Polymerizations were carried out in an argon-filled glovebox at room temperature using 1.5 mL glass vials equipped with a magnetic stir bar and a screw cap. An example for the homopolymerization of OxP monomers is given for OxP_Me_: OxP_Me_ (205 mg, 1.31 mmol, 30 eq.) was dissolved in dry DCM. In a separate glass vial, DBU (19 μL, 20 mg, 0.13 mmol, 3 eq.), TU (48 mg, 0.13 mmol, 3 eq.) and the initiator BnOH (4.5 μL, 4.7 mg, 0.04 mmol, 1 eq.) were dissolved in dry DCM. The total amount of DCM was determined to achieve a final monomer concentration of [Monomer]_0_ = 1 M (*V*_DCM_ = 1.281 μL). After 5 minutes, the initiator-catalyst solution was added to the monomer solution to start the polymerization. The mixture was stirred for 90 minutes and quenched by adding benzoic acid. The final polymer was obtained by precipitating twice in cold diethyl ether and drying under reduced pressure.

#### General procedure for polymerization kinetic experiments

Polymerizations for kinetic experiments were carried out in an argon-filled glovebox at room temperature using 1.5 mL glass vials equipped with a magnetic stir bar and a screw cap. In a glass vial, monomer was dissolved in dry solvent (DCM or toluene). In a separate glass vial, DBU (3 eq.), TU (3 eq.) and BnOH (1 eq.) were dissolved in dry solvent (DCM or toluene). The total amount of DCM was determined to achieve a final monomer concentration of [Monomer]_0_ = 1 M. After 5 minutes, the initiator-catalyst solution was added to the monomer solution to start the polymerization. Aliquots of the reaction were taken after set reaction times and quenched by adding benzoic acid. The solvent was evaporated under reduced pressure. An aliquot of the sample with the longest reaction time was precipitated twice in cold Et_2_O and dried under reduced pressure to obtain a purified sample. All samples were characterized by ^1^H NMR and SEC. The conversion rate was determined from the ratio of the integrated signals of monomer and polymer in ^1^H NMR spectra.

Additional methods, experimental conditions and full characterization are provided in the ESI.†

#### General procedure for degradation studies

##### Homopolymer degradation under accelerated conditions

A homopolymer stock solution was prepared by dissolving it in DCM. The stock solution was divided into separate vials (1.5 mL) and the solvent was evaporated under vacuum to form a polymer layer of ≃5 mg in each vial. A solution of sodium hydroxide (NaOH) in methanol (0.005 M) was added to the different vials to reach a final polymer concentration of 10 mg mL^−1^. The vials were placed in a thermoshaker set at room temperature. After the desired reaction time, the vials were removed from the thermoshaker and the solvent was evaporated under vacuum. The residual product was analyzed by SEC.

##### Homopolymer degradation under enzymatic conditions and in PBS solution

A similar procedure was employed to prepare a polymer layer (5 mg) in different vials (1.5 mL). A solution of PBS or lipase from *Pseudomonas cepacian* (LPC, ≥30 U mg^−1^) in PBS (100 U mL^−1^) was added to the different vials to reach a final polymer concentration of 10 or 5 mg mL^−1^. The vials were placed in a thermoshaker set at 37 °C. After the desired reaction time, the vials were removed from the thermoshaker and the solvent was lyophilized. The residual product was analyzed by SEC.

#### Photo-rheology experiment

The gelation behavior of the POxP_propylene_ polymer was studied by photo-rheology using an Anton Paar MCR302e rheometer with a 10 mm parallel-plate measuring system on a quartz bottom glass plate at 30 °C. For gelation, all solutions (w/v) were prepared in THF. To obtain a 10% hydrogel (*V* = 25 μL) as a first step, 12.50 μL of a 20% POxP_propylene_ solution (0.5 μmol; 13.6 μmol allyl groups) was added to 25 μL of dry THF in a dark Eppendorf tube. To ensure a 1/1 thiol to ene ratio, 2.62 μL of a 20% dithiothreitol solution (6.8 μmol; 13.6 μmol thiol groups) was added to the tube, as well as 1.25 μL of a 1% Irgacure-2959 solution (55.7 nmol). Directly afterwards an aliquot of 20 μL of the mixed solution was dispensed onto the glass plate with a 0.05 mm gap. To determine the storage and loss moduli, analyses were performed using a frequency of 1 Hz and a shear strain of 1%. After equilibration for 10 min, UV light irradiation was performed with a 365 nm LED in a Prizmatix PRI Combi-LED 5 at 80% of maximum power and a 8 mm Liquid Light guide installed under the quartz bottom plate of the rheometer.

## Results and discussion

### Synthesis of monomer precursor

OxP_TFA_ was synthesized in three steps starting from 4-piperidone monohydrate hydrochloride with an overall yield of 61% (Fig. 1a). Boc-protection and Bayer-Villiger ring-expansion using *meta*-chloroperoxybenzoic acid (*m*CPBA), afforded 4-*tert*-butoxycarbonyl-1,4-oxazepan-7-one (OxP_Boc_), which was previously reported as a monomer for ROP by the groups of Hadjichristidis^14,35^ and Waymouth.^36^ Using a recently reported greener oxidation procedure by Giraudo *et al.*,^44^ we aimed to improve the ring-expansion affording OxP_Boc_. Using this method, we avoided the use of peroxy acids and carboxylic acid waste products, which are detrimental to the basic ROP methodology if remaining traces are not removed from the OxP building blocks and monomers. Following the OXONE® based procedure, OxP_Boc_ was obtained with a yield of 80% compared to 64% using the *m*CPBA mediated Bayer-Villiger ring-expansion. Finally, treatment of OxP_Boc_ with a solution of trifluoroacetic acid (TFA) in DCM yielded OxP_TFA_ as a colorless salt after purification. Since ring-opening of 1,4-oxazepan-7-one could potentially occur under acid conditions, we performed kinetic studies to investigate the Boc-deprotection of OxP_Boc_ toward OxP_TFA_ by ^1^H NMR spectroscopy (Fig. S1†). The complete removal of the Boc-protecting group was confirmed after 30 minutes of reaction time, as observed *via* the disappearance of the ^1^H NMR signals of the methyl groups of the Boc-protecting group (*δ* = 1.41 ppm). No significant side reactions were observed during successful deprotection and a reaction time of up to 1 h, and small amounts (<5%) of impurities were easily removed by precipitation. Only after extended reaction times of 20 h, significant amounts of the ring-open form of OxP_TFA_, 3-[(2-hydroxyethyl)amino]propanoic acid were observed and confirmed by ^1^H NMR spectroscopy and ESI-TOF-MS spectrometry. This observation further underlines the robustness of our synthetic route towards a modular OxP monomer platform based on 1,4-oxazepan-7-one trifluoroacetate for the synthesis of functional poly(amino ester) derivatives.

### Acylation of OxP_TFA_ toward functional monomers

**Fig. 2:**
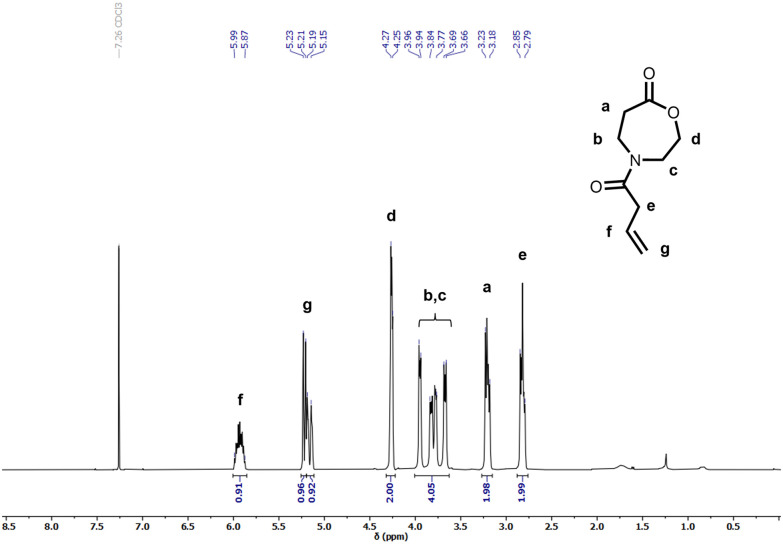
^1^H NMR spectrum of 4-(But-3-enoyl)-1,4-oxazepan-7-one (OxP_propylene_).

Several OxP monomers were synthesized in a one-step acylation of OxP_TFA_ under alkaline conditions. OxP_TFA_ was reacted with different acyl chlorides to afford the four monomers with yields ranging from 47% to 69% after purification. Purification *via* silica flush chromatography allowed the isolation of all OxP monomers in high purity. A detailed characterization of the four *N*-acylated-1,4-oxazepan-7-one monomers *via*^1^H and ^13^C NMR is provided in Fig. 2, Fig. S2–S3 and S21–S33.† Different moieties, including ethyl methyl sulfide, propylene, butyl, and methyl pendant groups were installed *via* acylation of the OxP_TFA_ precursor to synthesize a range of OxP monomers (Fig. 1b). 4-(But-3-enoyl)-1,4-oxazepan-7-one (OxP_propylene_) bears a double bond in the side chain, which could be further modified post-polymerization, *e.g.*, by addition reactions or crosslinking.^45^

4-(3-methylthio)propanoyl-1,4-oxazepan-7-one (OxP_EtSMe_) contains the analogueous side chain of the amino acid methionine. The thioether group opens the possibility of oxidizing the sulfide to a sulfoxide or sulfone after polymerization, which could impact the hydrophilicity of the polymeric chain.^46–54^ 4-Pentanoyl-1,4-oxazepan-7-one (OxP_But_) serves as the aliphatic side chain counterpart to OxP_EtSMe_. Meanwhile, OxP_Me_ yields a monomer that dissolves readily in water.

### Organocatalytic ring-opening homopolymerization of *N*-acylated-1,4-oxazepan-7-ones

**Scheme 1:**
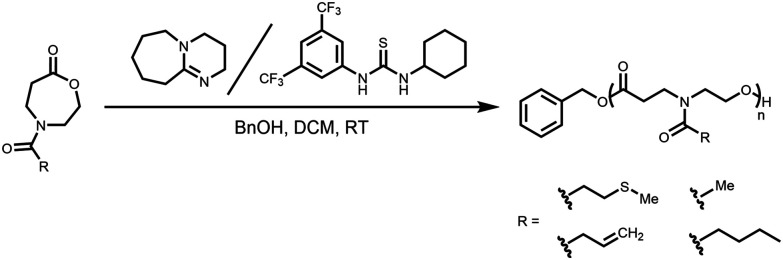
Organocatalytic ROP of *N*-acylated-1,4-oxazepan-7-ones.

**Fig. 3:**
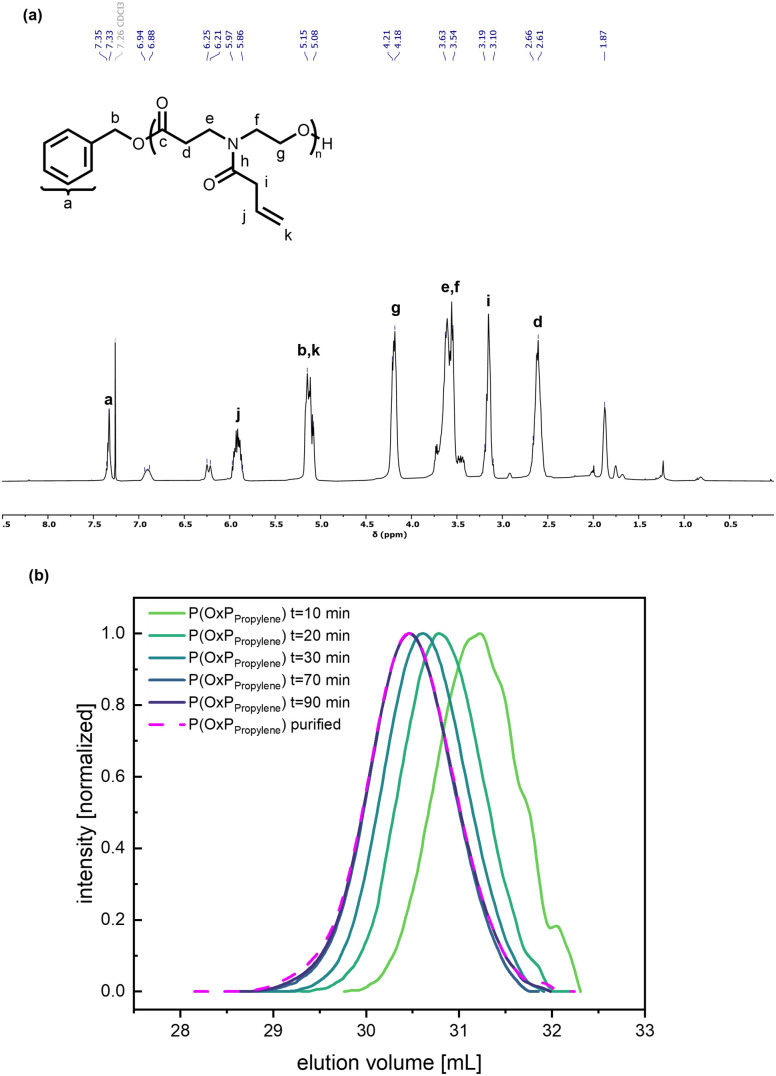
(a) ^1^H NMR spectrum of P(OxP_propylene_) with assigned signals. (b) SEC elution traces of OxP_propylene_ organocatalytic ROP after given reaction times (green to purple gradient, solid lines). Purified P(OxP_propylene_) sample from the *t* = 80 min sample (pink, dashed line). RI signal; eluent: THF, 25 °C; standard: PMMA.

The organocatalytic ROP of the four OxP monomers was carried out at room temperature in DCM using benzyl alcohol (BnOH) as initiator (Scheme 1). The combination of 1,8-diazabicyclo[5.4.0]undec-7-en (DBU) and thiourea catalyst 1-[3,5-bis(trifluoromethyl)phenyl]-3-cyclohexyl thiourea (TU) was used as an organocatalytic system, as it was reported to be efficient for the ROP of polyesters and polycarbonates.^14,35,36,41–43,55^ Further polymerization parameters were selected as follows: [M]_0_/[BnOH]_0_/[DBU]_0_/[TU]_0_ = 30/1/3/3; [M]_0_ = 1 M and *t* = 80–90 min. The homopolymers were characterized *via* NMR spectroscopy, SEC and MALDI-TOF MS (Fig. S14–17, Fig. S35–50†). The characterization data for the POxP homopolymers are summarized in Table 1. Polymerizations of the OxPs show high monomer conversions, with values ranging from 89% to complete conversion. The ^1^H NMR spectrum of P(OxP_propylene_) (Table 1, entry 1) is shown as an example for the POxP homopolymers (Fig. 3a). All homopolymers exhibit unimodal distribution, narrow dispersity values determined *via* SEC (*Ð* = 1.09–1.13), and good to excellent agreement of the number average molar mass calculated from the ^1^H NMR spectra 
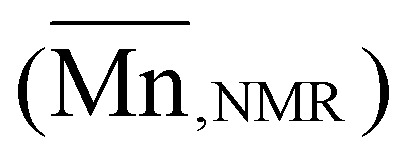
 with the theoretical molar masses 
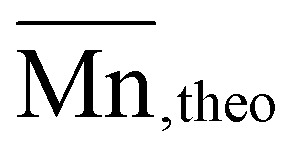
 (Table 1). These results are indicative of well controlled chain-growth polymerization. The polymers were further characterized by MALDI-TOF-MS (Fig. S14–17†). The resulting spectra show benzyl alcohol-initiated polymer species for the POxP_Me_, POxP_But_ and POxP_propylene_ homopolymers, with potassium as counter ion and the corresponding Δ *m*/*z* of the individual monomer mass.

**Table 1:** Characterization data of the organocatalytic ROP of OxP monomers

Entry	Monomer (M)	Reaction time [min]	Conv.^d^ [%]	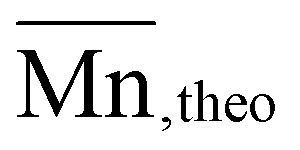 e [g mol^−1^]	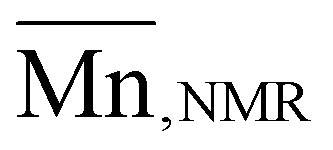 f [g mol^−1^]	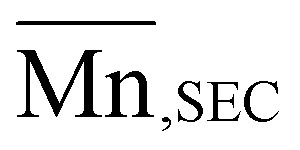 g [g mol^−1^]	*Ð* g
1	OxP_propylene_ a^,^^b^	90	100	5600	4600	2400	1.10
2	OxP_But_ a^,^^b^	90	89	5400	4500	3900	1.09
3	OxP_EtSMe_ a^,^^b^	80	99	6500	5200	2300	1.10
4	OxP_Me_ a^,^^b^	80	100	4800	4700	2500 h	1.13 h
5	OxP_But_ a^,^^c^	100	94	5700	5300	6000	1.09

a r.t.; [M]_0_/[BnOH]_0_/[DBU]_0_/[TU]_0_ = 30/1/3/3; [M]_0_ = 1 M.

b Solvent: dichloromethane.

c Solvent: toluene.

d Conversion was calculated from ^1^H NMR spectra of the crude products, using characteristic signals for monomers and polymers, calculated as follows: [M]_*t*_/([M]_*t*_ + [P]_*t*_).

e Theoretical number-average molar mass, calculated from feed ratio and monomer conversion from crude ^1^H NMR spectrum as follows: ([M]_0_/[BnOH]_0_) × conversion × (M.W. of OxP) + (M.W. BnOH).

f Calculated from^1^H NMR spectrum of isolated product, using signal integrals of the initiator (BnOH) and the polymer.

g Determined *via* SEC analysis (RI signal, eluent: THF, 25 °C, standard: PMMA).

h Determined *via* SEC analysis (RI signal, eluent: DMF, 50 °C, standard: PMMA).

**Fig. 4:**
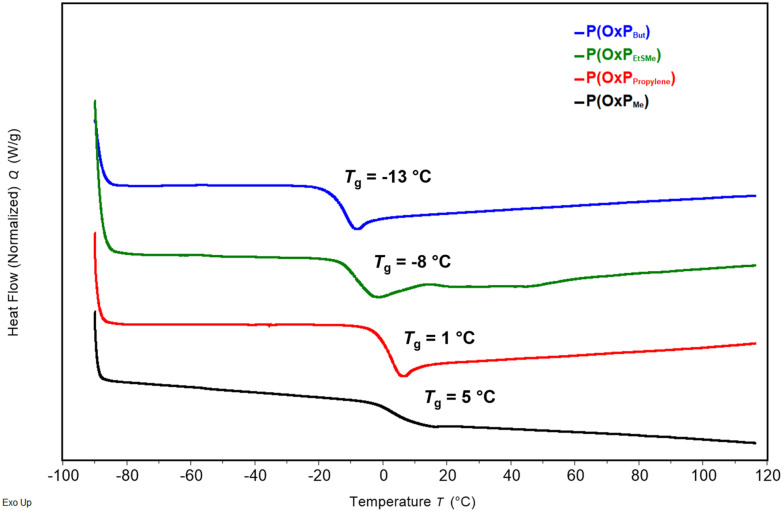
DSC thermogram of POxP homopolymers: POxP_But_, POxPEtS_Me_, POxP_propylene_, POxP_Me_.

The DSC analysis of the four OxP homopolymers yielded distinct glass transition temperatures (*T*_g_) (Fig. 4). The amorphous character of POxPs is attributed to the irregular orientation of the amide bonds in the polymer backbone.^14^ The lowest *T*_g_ was measured for POxP_But_ (−13 °C), while the highest *T*_g_ was measured for POxP_Me_ (5 °C). This observation is consistent with our expectations, as the *T*_g_ decreases with increasing aliphatic side chain length for POxP_Me_ compared to POxP_But_.

The well controlled polymerization of OxP monomers allowed the synthesis of a diblock copolymer P(OxP_Me_)_25_-*b*-P(OxP_Boc_)_10_ using similar reaction conditions. The synthesis proceeded by polymerization of the OxP_Me_ monomer, followed by addition and polymerization of OxP_Boc_ after complete consumption of the first monomer. The ^1^H NMR spectrum of the block copolymer (Fig. S51†) showed the presence of both OxP_Me_ and OxP_Boc_ repeating units, which was also confirmed by MALDI-TOF MS (Fig. S52†). The molar mass of the block copolymer determined by NMR 
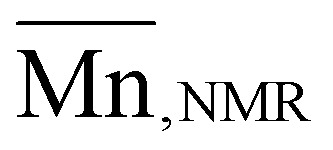
 = 5400 g mol^−1^, is also in good agreement with the theoretical molar mass 
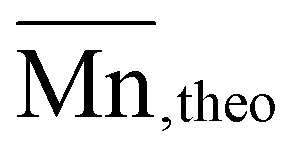
 (Table S2†). Finally, characterization by SEC revealed the synthesis of the diblock copolymer P(OxP_Me_)_25_-*b*-P(OxP_Boc_)_10_ with a low dispersity *Ð* = 1.1 (Fig. S53†).

### Kinetic studies of organocatalytic ROP

**Fig. 5:**
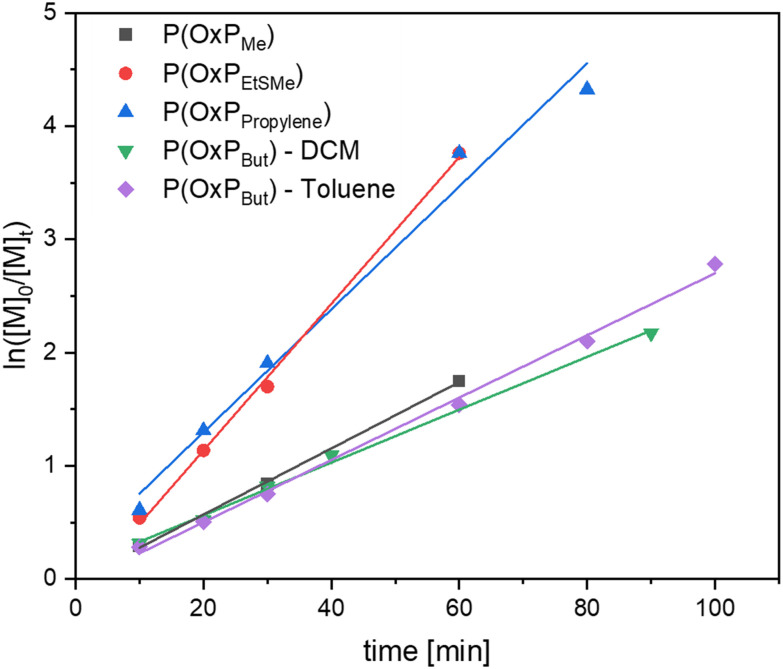
Kinetic plot of organocatalytic ROP of OxPs in DCM. Semilogarithmic plots over the reaction time (dots with varying shapes and colors for every OxP homopolymer), fitted by linear regression (line with varying color for every OxP homopolymer).

Kinetic investigations of the organocatalytic ROP for the four OxP monomers with DBU/TU as catalysts were carried out under the previously mentioned reaction conditions. Aliquots of the running polymerization were analyzed *via*^1^H NMR spectroscopy and SEC (Fig. 3, Fig. 5, and Fig. S4–13†). The elugrams of the P(OxP_Proylene_) polymerization (Fig. 3b) at each measured time point of the polymerization reactions are unimodal and give narrow dispersities (*Ð* < 1.1). As illustrated in Fig. S13,†
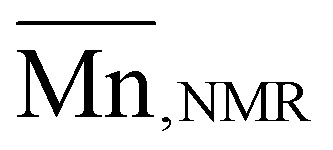
 increases linearly with the monomer conversion.

The semilogarithmic plots of monomer concentration *versus* reaction time follow a linear trend, indicating first-order kinetics for the polymerizations of the four monomers (Fig. 5). OxP_But_ has the lowest reactivity, followed by slightly more reactive OxP_Me_. Polymerization kinetics for OxP_But_ were also conducted in toluene and were found to be slightly faster than in DCM. Given that not all the monomers are soluble in toluene, DCM was the solvent of choice for all the polymerizations. The reactivities of OxP_EtSMe_ and OxP_propylene_ were found to be higher than those of OxP_Me_ and OxP_But_. The group of Hadjichristidis suggested that aromatic side chains in OxP monomers increase the basicity of the lactone functionality and efficiency of catalyst activation compared to the aliphatic side chains.^14^ However in our case, electronic effects are less likely to have a similar impact in the case of OxP_EtSMe_ and OxP_propylene_ compared to OxP_Me_ and OxP_But_.

### Homopolymer degradation in organic and aqueous media

The degradability of POxPs was evaluated by performing degradation studies under accelerated and enzymatic conditions,^56,57^ in accordance with previous studies.^58–60^ The degradation tests were carried out using the water-soluble P(OxP_Me_) with a molar mass 
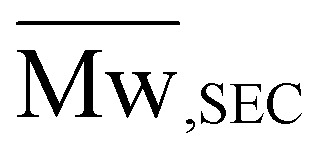
 of 3500 g mol^−1^ and a dispersity of *Ð* = 1.1. Degradation under accelerated conditions was performed at room temperature (25 °C) using a polymer concentration of 10 mg mL^−1^ in a low-concentrated solution of NaOH in methanol (0.005 m). As shown in Fig. S18,† the initial homopolymer is almost completely degraded in short reaction times (5–60 min), showing a shift toward lower molar masses (
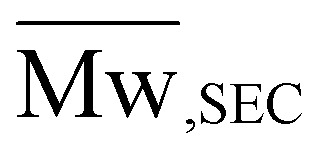
 = 420 g mol^−1^, or 88% decrease in 
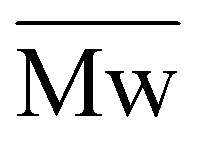
). Only low molar mass species with a multimodal distribution remain after the test.

**Fig. 6:**
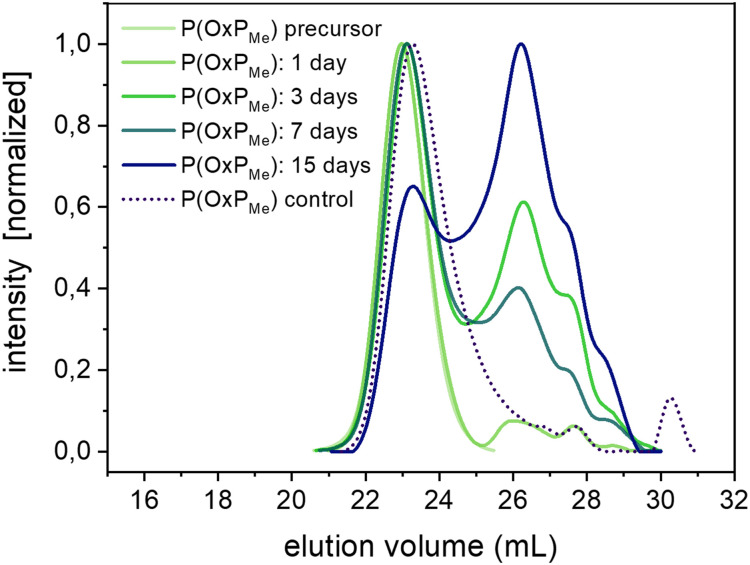
SEC elution traces of P(OxP_Me_) degradation under enzymatic conditions: [P(OxP_Me_)] = 5 mg mL^−1^, [LPC] = 100 U mL^−1^, PBS, 37 °C (DMF, standard: PMMA).

Additionally, degradation studies were performed under enzymatic catalysis conditions at 37 °C using lipase from *Pseudomonas cepacian* (LPC) in PBS buffer (100 U mL^−1^). After 15 days and at a polymer concentration of 10 mg mL^−1^, the formation of low molar mass species is observed, resulting in a decrease in molar mass to 
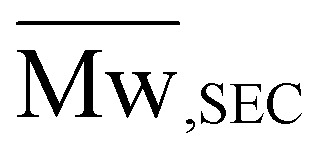
 = 2700 g mol^−1^ (or a 23% decrease in 
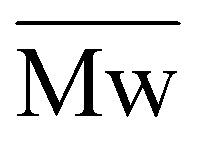
) and a broadening of the dispersity (Fig. S19†). The main polymer species remains present in solution, indicating a slow degradation process. By lowering the polymer concentration to 5 mg mL^−1^, the molar mass is further decreased to 
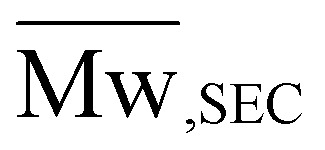
 = 1400 g mol^−1^ (or 60% decrease in 
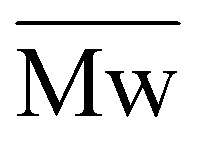
), followed by a broadening of the dispersity (Fig. 6 and Table S1†). Under these conditions, a significant increase in the relative fraction of the lower molar mass species is observed, which confirms faster degradation at a lower polymer concentration and fixed amount of lipase. Control degradation experiments of P(OxP_Me_) in PBS after 15 days also revealed slight degradation with the formation of lower molar mass species. Further investigation was conducted on a P(OxP_Me_) precursor (
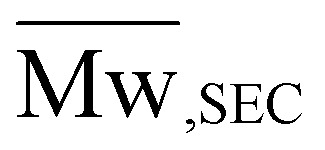
 of 3900 g mol^−1^ and *Ð* = 1.1) to determine the potential degradability of the polymer in PBS at 37 °C, closer to physiological conditions (Fig. S20†). After 50 days of reaction, an increase in the fraction of low molar mass species was observed, as well as an increase in dispersity (
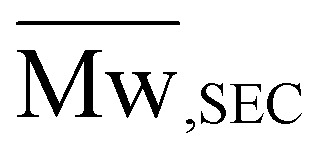
 = 2100 g mol^−1^, or 46% decrease in 
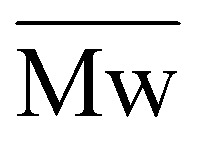
). In summary, the amide linkages in the side chains of the poly(amino ester)s likely increase their hydrolytic stability, but decrease the potential for pH-responsiveness behaviour in PAEs with alkyl side chains.^16^ Monomer and polymer modification strategies that open opportunities for pH-dependent degradation of PAEs are currently under investigation.

### Post-polymerization modification of PAE

**Fig. 7:**
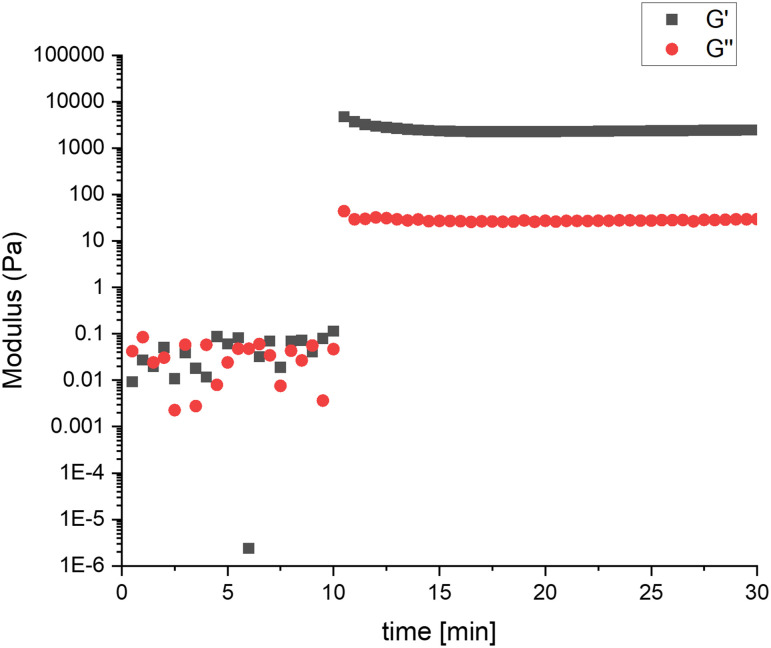
Storage and loss moduli (*G*′ and *G*′′) as a function of time for POxP_propylene_ photo-induced cross-linking with DTT before and after irradiation (10 min) with 365 nm UV light.

The diverse functionalities conferred by the PAE pendant chains pave the way for post-modification reactions and tailored materials properties. As a proof of concept, a gelation experiment was performed by thiol–ene coupling using the POxP_propylene_ homepolymer bearing allyl functional groups in the pendent side chains. Similar to previous gelation studies on poly(oxazoline),^61,62^ a photo-induced cross-linking experiment was conducted by reacting POxP_propylene_ with dithiothreitol (DTT) cross-linker using Irgacure-2959 as photoinitiator. The experiment was carried out with an equimolar ratio of thiol to ene in THF at 30 °C to obtain a final 10% (w/v) gel. As shown in Fig. 7, no viscoelastic behavior is visible before irradiation (0–10 min). When UV light is applied (*λ* = 365 nm, 10 min), *G*′ increases and crosses *G*′′ within 30 s, indicating gel formation. The thiol–ene reaction takes place when UV light is applied, thus crosslinking of the polymers occurs with DTT. As a result, a polymer network is formed as observed in the rheological experiment. The POxP_propylene_ network exhibits a storage modulus *G*′ of about 3000 Pa, which is a similar gel strength as obtained in the case of the poly(oxazoline)-based network reported in the literature.^61,62^ These experiments confirm the successful functionalization of POxP_propylene_*via* photo-induced thiol-ene cross-linking, and potential for post-polymerization modification of functional PAEs in general.

## Conclusions

A three-step approach involving Boc-protection, Bayer-Villiger ring-expansion or oxidation with OXONE®, followed by Boc-deprotection was developed to synthesize a universal monomer precursor, OxP_TFA_, for the synthesis of new functional monomers. A one-step acylation of OxP_TFA_ enabled the synthesis of four monomers with yields ranging from 47 to 69% and various pendant groups, including butyl, propylene, ethyl methyl sulfide, and methyl side chains.

The organocatalytic ROP of the four monomers at room temperature using DBU and TU as catalysts, yielded polymers with high monomer conversions (≥ 89%) and low dispersities *Ð* ≤ 1.16. Kinetic studies of the homopolymerizations exhibited a linear increase in the 
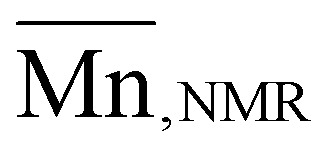
 with the monomer conversion and first-order kinetics for all four monomers. Furthermore, a photo-rheology experiment involving a thiol–ene cross-linking reaction of POxP_propylene_ showed that pendant allyl functionalities of PAEs can be used for post-polymerization reactions to open avenues in material functionalization. Finally, degradation studies of POxP_Me_ under accelerated conditions revealed near-complete degradation of the polymer. Conducting the degradation in PBS solution and at body temperature allowed a partial degradation of the polymer, with the formation of species in the low molar mass region. The use of LPC as enzyme promoted the degradation of the polymer, by increasing the fraction of smaller species formed.

The development of this three-step synthetic route toward functional monomers provides new opportunities in the design of functional and degradable poly(amino ester) polymers, including blocked architectures. The ability to introduce diverse functionalities opens the way for post-polymerization reactions and enables fine-tuning of material properties, thereby opening avenues in a range of applications.

## Author contributions

Tino Mackiol: conceptualization, data curation, investigation, methodology, validation, visualization, writing – original draft, review & editing; Chloé Pascouau: data curation, investigation, methodology, validation, visualization, review & editing; Manuel Nagel: investigation; Tamara M. Bizmark: investigation; Luca Montesel: investigation; Jochen Fischer-Schuch: conceptualization, methodology, validation, review&editing; Pol Besenius: conceptualization, validation, funding acquisition, resources, writing – review & editing.

## Conflicts of interest

There are no conflicts to declare.

## Supplementary Material

PY-016-D5PY00522A-s001

## Data Availability

The data supporting this article have been included as part of the ESI (Fig. S1–S50†).
