# Supplementary material for: 1,4-Oxazepan-7-one trifluoroacetate: a modular monomer precursor for the synthesis of functional and biodegradable poly(amino esters)
Source: Polym Chem. 2025 Jul 3;16(30):3450–8. doi: 10.1039/d5py00522a (PMC12242916; doi:10.1039/d5py00522a)
Supplement: PY-016-D5PY00522A-s001 [file PY-016-D5PY00522A-s001.pdf]

Supplementary Information (SI) for Polymer Chemistry.
This journal is © The Royal Society of Chemistry 2025

Supporting Information

1,4-Oxazepan-7-one trifluoroacetate: a modular monomer precursor for the synthesis of functional and biodegradable poly(amino esters)

Tino Mackiol,[a] Chloé Pascouau,[a] Manuel Nagel,[a] Tamara M. Bizmark,[a] Luca Montesel,[a] Jochen Fischer-Schuch,[b] and Pol Besenius*[a]

1. **Supplementary Data for OxP Monomer Synthesis** **2**
2. **Supplementary Data for POxP Homopolymers** **4**
3. **Synthetic Procedure and Characterization for OxP Monomers and Catalyst** **16**
4. **Synthetic Procedure and Characterization for POxP Homopolymers** **22**
5. **NMR Data of OxP monomers and catalyst** **25**
6. **NMR Data of OxP homopolymers** **32**
7. **Supplementary Data for $P(OxP_{Me})_{25}$-b-$P(OxP_{Boc})_{10}$ Block Copolymer Synthesis** **40**

1. Supplementary Data for OxP Monomer Synthesis

Kinetic of OxPBoc deprotection in TFA/DCM

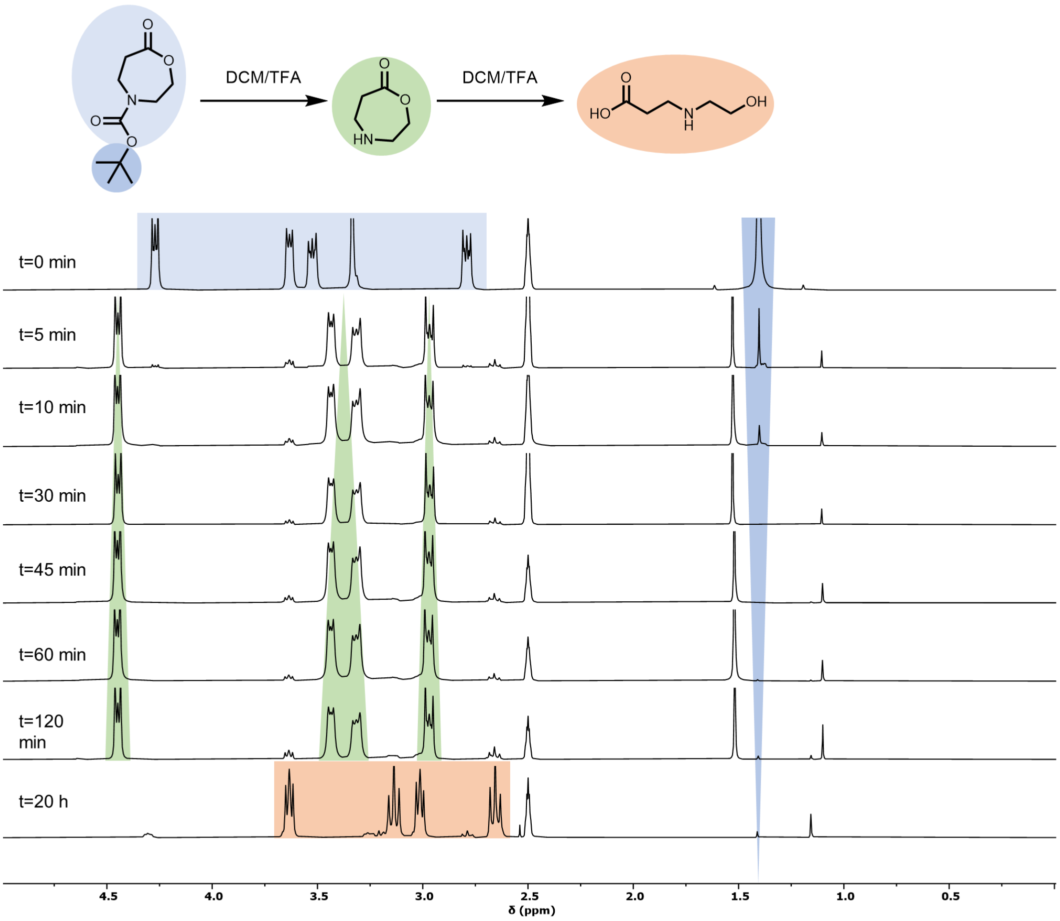

Fig. S1 Stacked ^{1}H NMR spectra of Boc-deprotection reaction with OxP_{Boc} in TFA/DCM solution after different reaction times (400 MHz, DMSO-d_6). OxP_{Boc} and its corresponding signals are shown in blue. 1,4-oxazepan-7-on is shown in green. The product of the ring opening 3-[(2-hydroxyethyl)amino]propanoic acid is shown in orange.

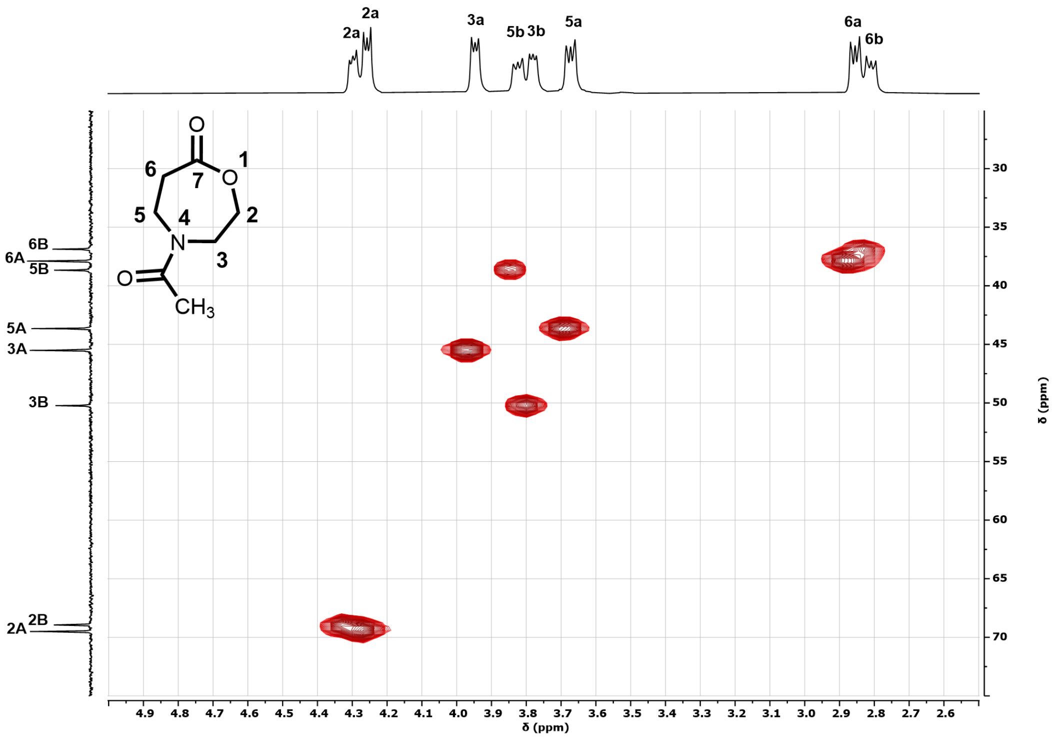

Fig. S2 HSQC of **OxP$_{Me}$** (400 MHz, $CDCl_3$).

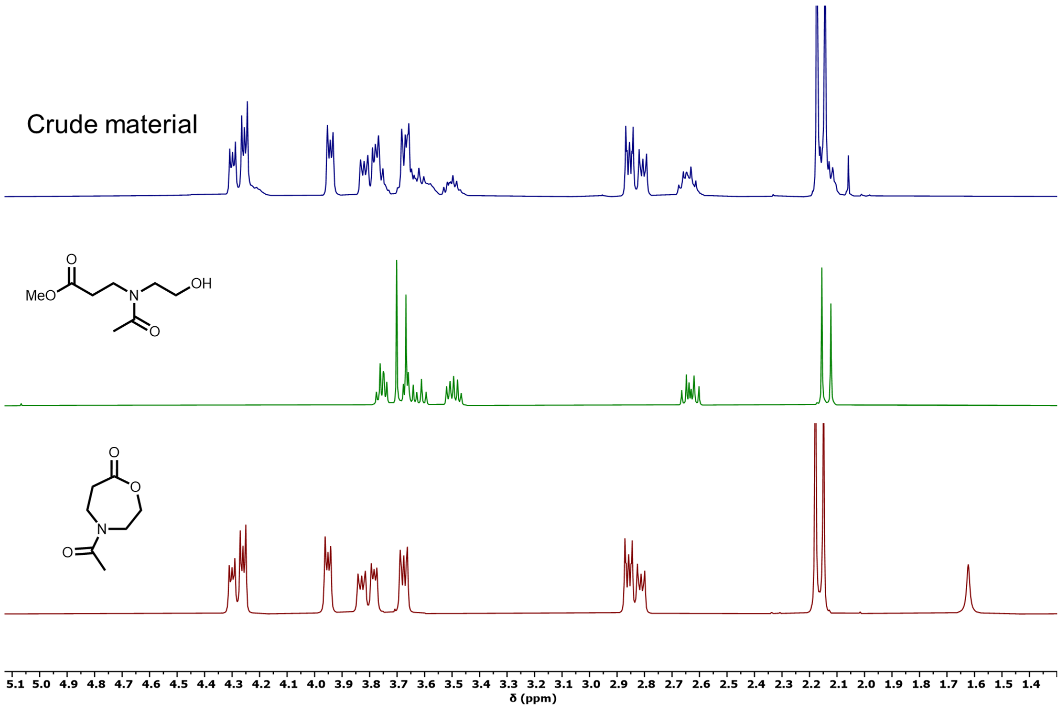

Fig. S3 Stacked 1H NMR spectra from different steps in the monomer synthesis of OxPMe. The upper spectrum refers to crude material before workup with silica column chromatography. The middle spectrum shows collected fraction of side product, which was found to be methyl 3-(N-(2-hydroxyethyl)acetamido)propanoate. The lower spectrum shows the spectrum of purified monomer OxPMe.

2. Supplementary Data for POxP Homopolymers

Kinetic Experiments

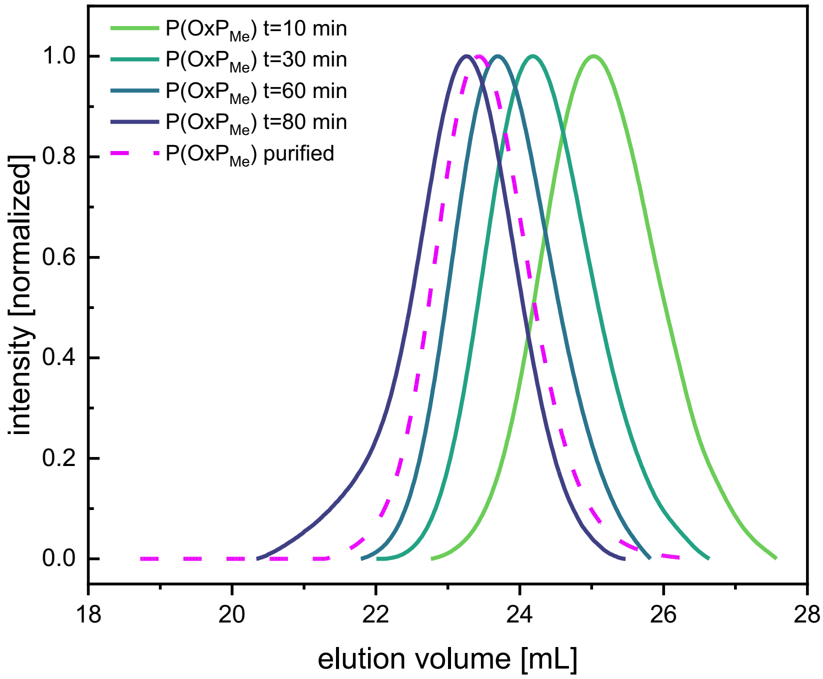

Fig. S4 SEC elution traces of **OxP$_{Me}$** polymerization in DCM after given reaction times (green to purple gradient, solid lines). Purified **P(OxP$_{Me}$)** sample from t=80 min sample (pink, dashed line)(RI signal, eluent: DMF, 50°C, standard: PMMA).

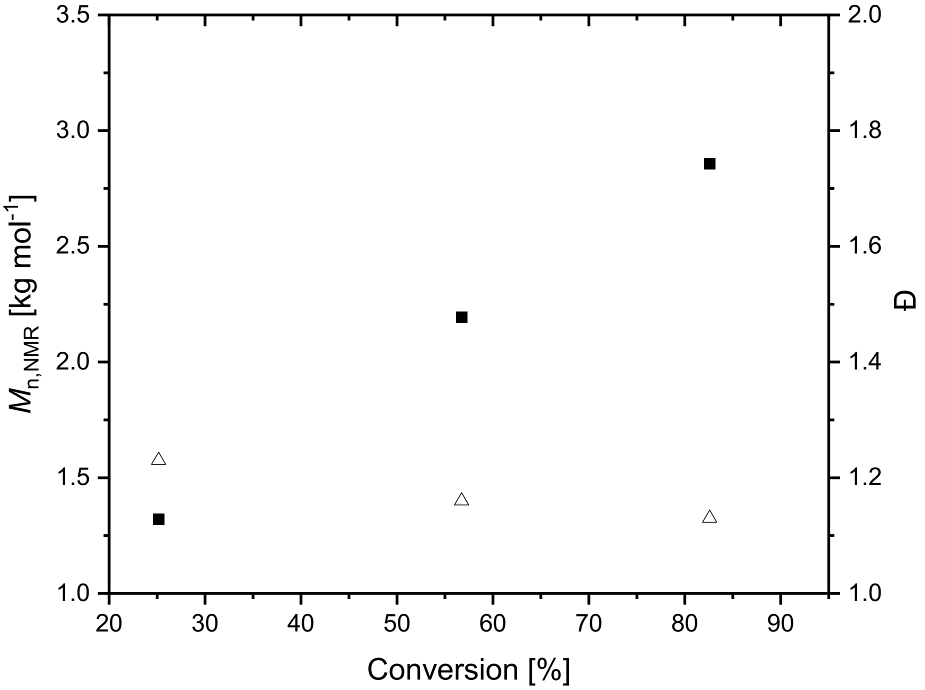

Fig. S5 Plot of $M_{n,NMR}$ and disperity versus monomer conversion for **OxP$_{Me}$** polymerization in DCM.

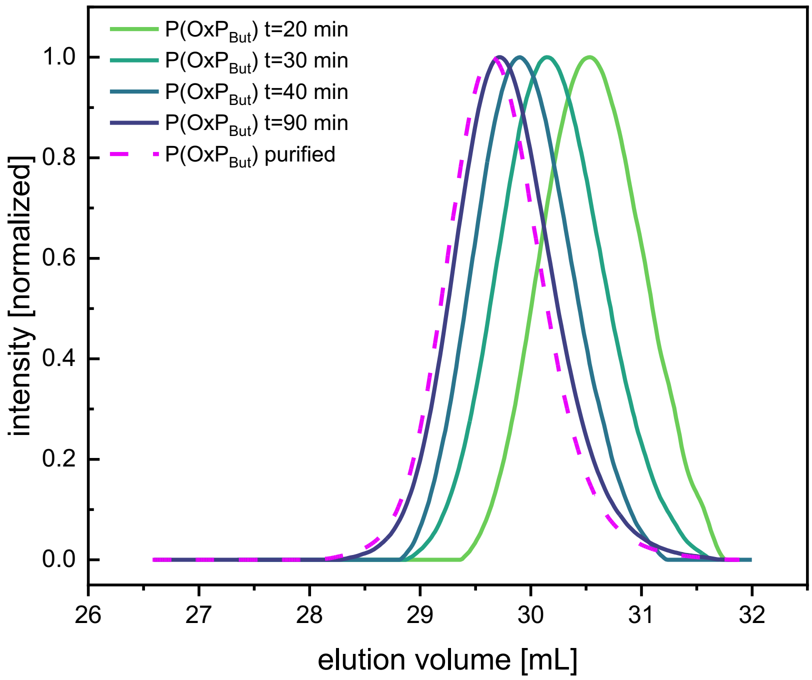

Fig. S6 SEC elution traces of **OxP$_{But}$** polymerization in DCM after given reaction times (green to purple gradient, solid lines). Purified **P(OxP$_{But}$)** sample from t=90 min sample (pink, dashed line)(RI signal, eluent: THF, 25°C, standard: PMMA).

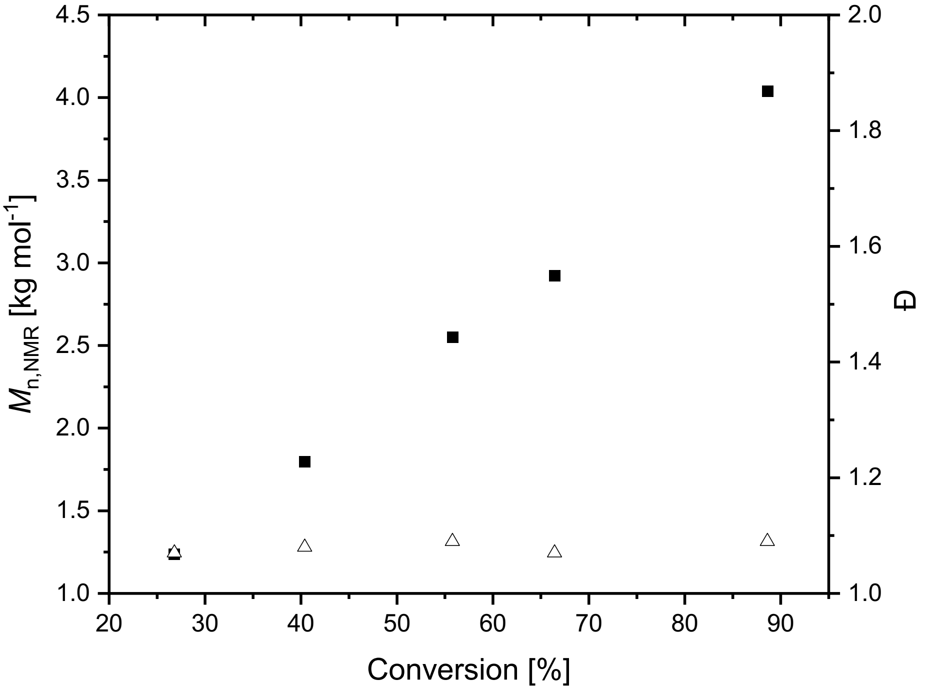

Fig. S7 Plot of $M_{n,NMR}$ and disperity versus monomer conversion for **OxP$_{But}$** polymerization in DCM.

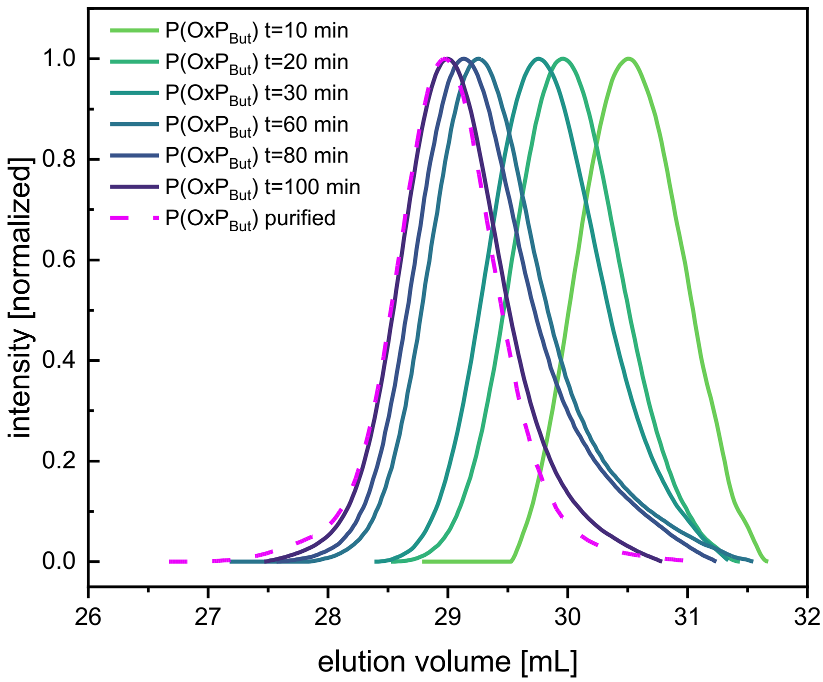

Fig. S8 SEC elution traces of **OxP$_{But}$** polymerization in toluene after given reaction times (green to purple gradient, solid lines). Purified **P(OxP$_{But}$)** sample from t=100 min sample (pink, dashed line)(RI signal, eluent: THF, 25°C, standard: PMMA).

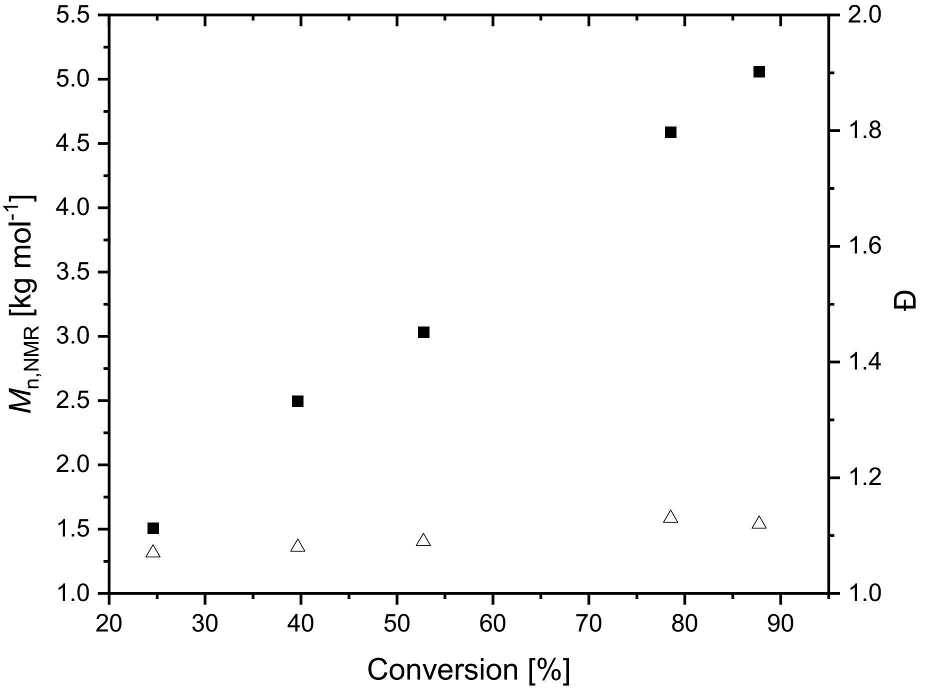

Fig. S9 Plot of $M_{n,NMR}$ and disperity versus monomer conversion for **OxP$_{But}$** polymerization in toluene.

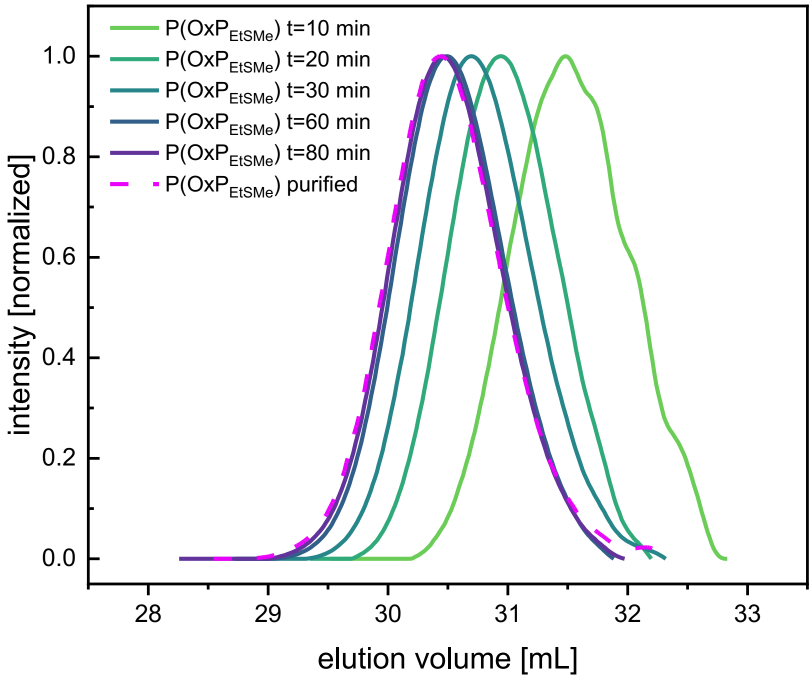

Fig. S10 SEC elution traces of **OxP$_{EtSMe}$** polymerization in DCM after given reaction times (green to purple gradient, solid lines). Purified **P(OxP$_{EtSMe}$)** sample from t=80 min sample (pink, dashed line)(RI signal, eluent: THF, 25°C, standard: PMMA).

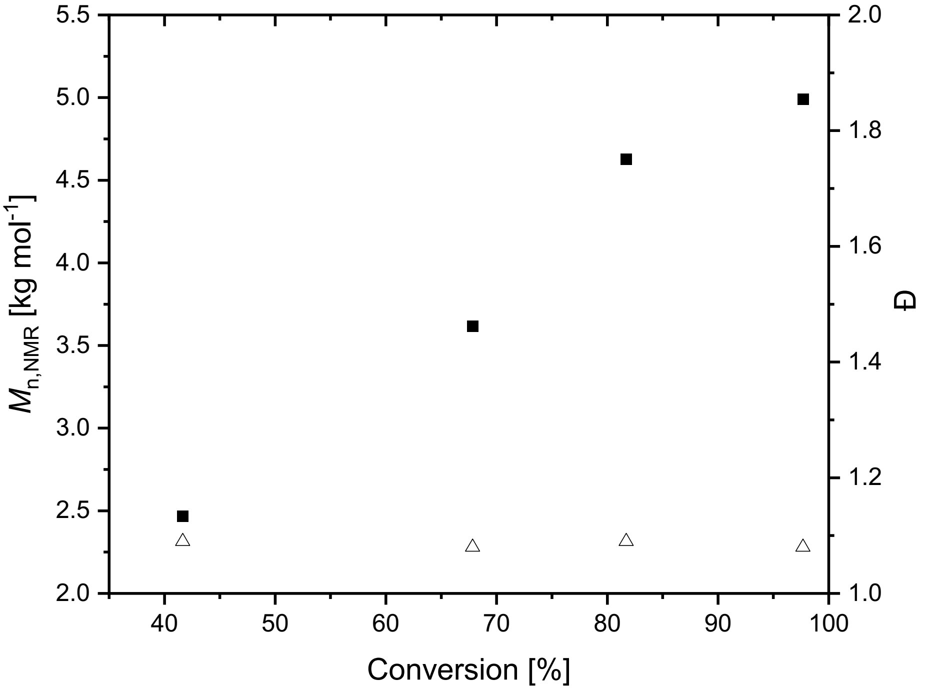

Fig. S11 Plot of $M_{n,NMR}$ and disperity versus monomer conversion for **OxP**$_{\mathbf{EtSMe}}$ polymerization in DCM.

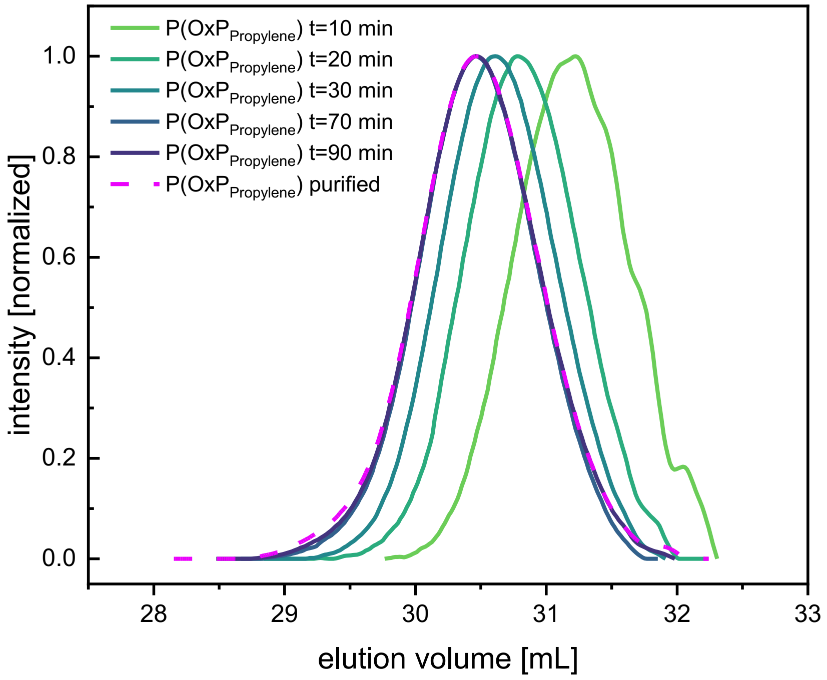

Fig. S12 SEC elution traces of **OxP**$_{\mathbf{Propylene}}$ polymerization in DCM after given reaction times (green to purple gradient, solid lines). Purified **P(OxP**$_{\mathbf{Propylene}}$**)** sample from t=90 min sample (pink, dashed line)(RI signal, eluent: THF, 25°C, standard: PMMA).

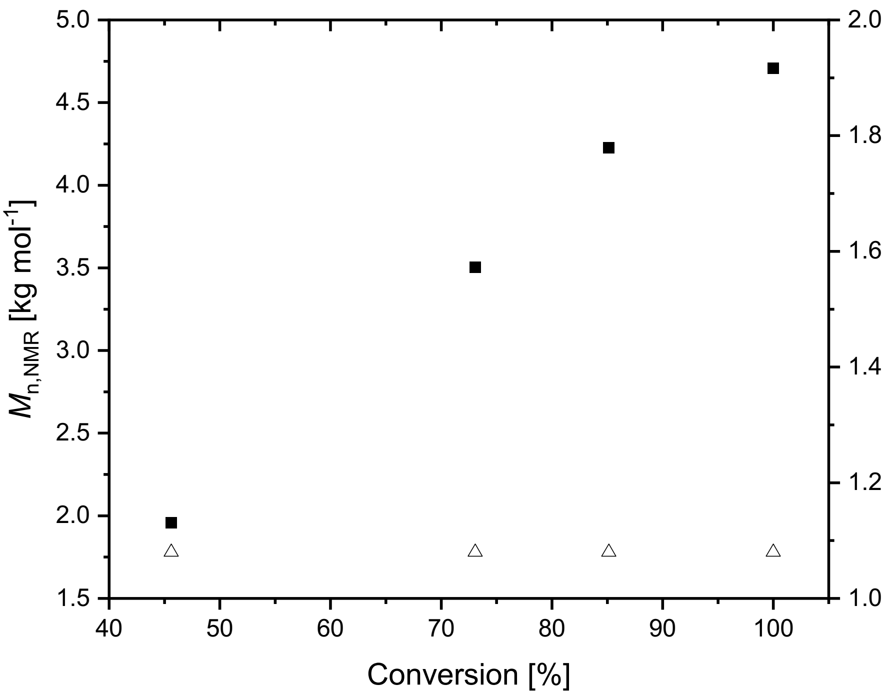

Fig. S13 Plot of $M_{n,NMR}$ and disperity versus monomer conversion for $\mathbf{OxP_{Propylene}}$ polymerization in DCM.

MALDI-Tof MS measurements

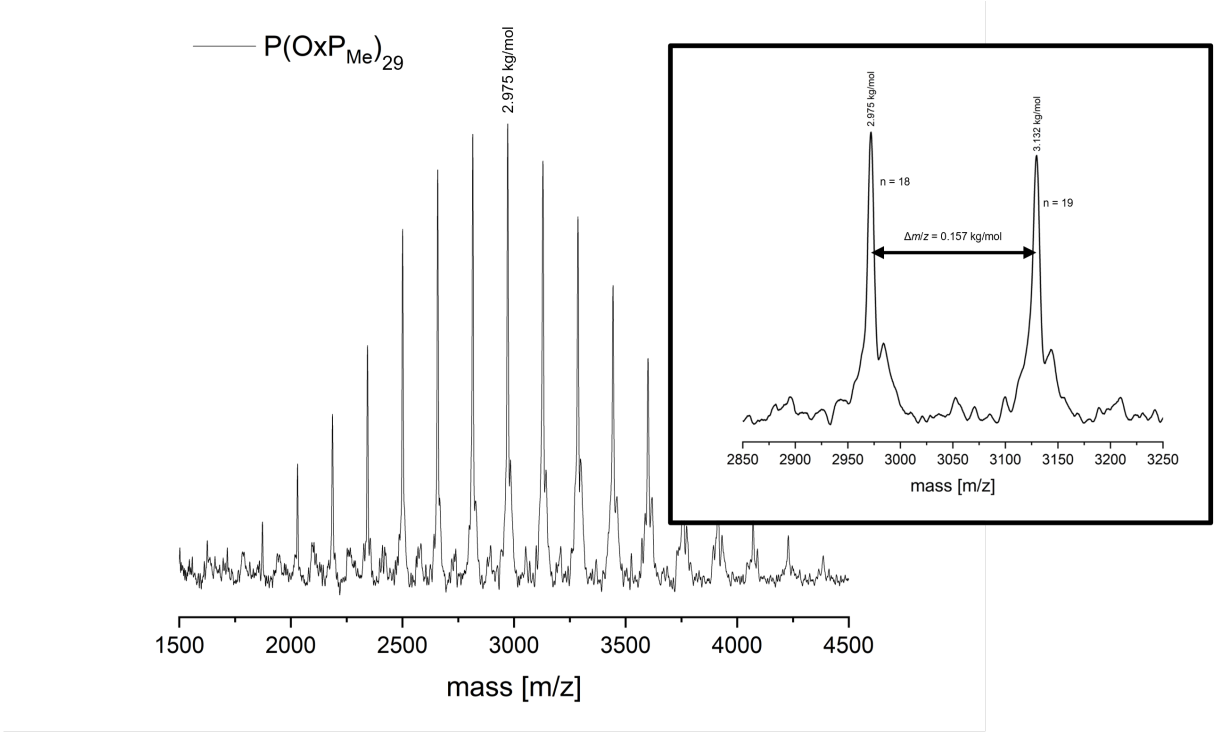

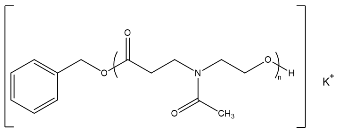

$$m/z = 0.108\ \text{kg/mol} + n \cdot 0.157\ \text{kg/mol} + 0.039\ \text{kg/mol}$$

Fig. S14 MALDI-ToF mass spectrum of purified **POxP$_{Me}$** (DCTB, KTFA).

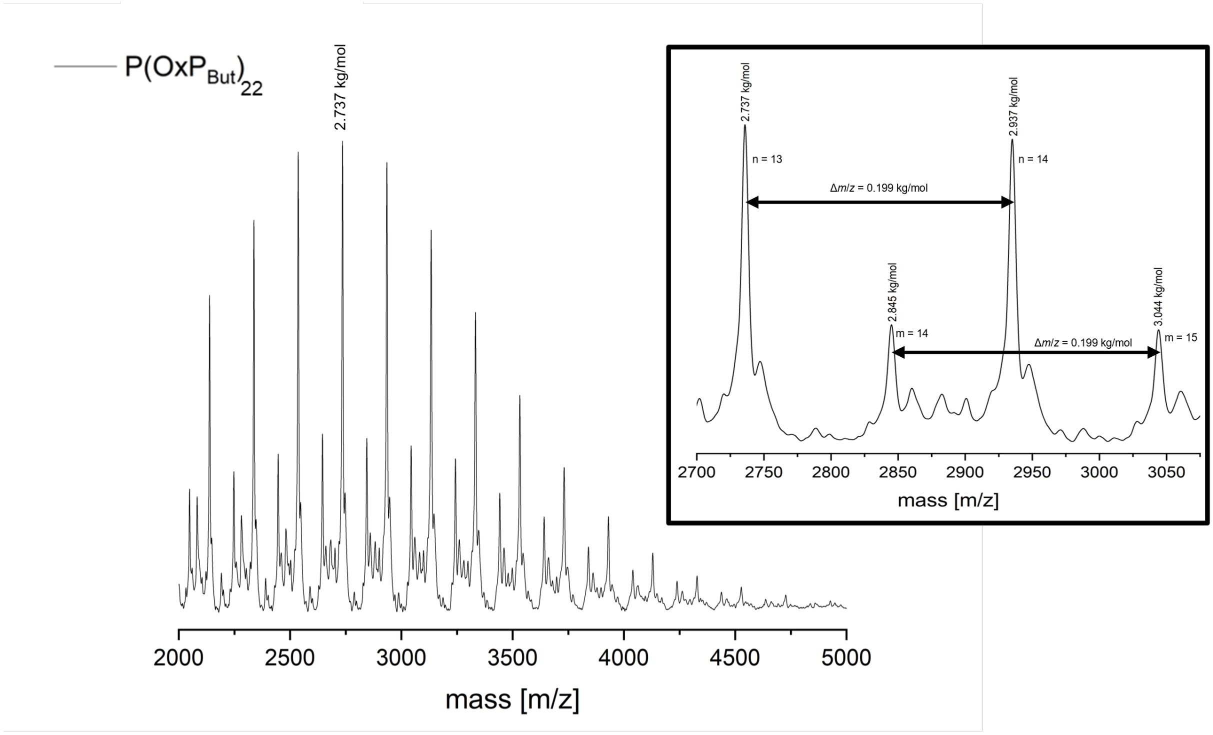

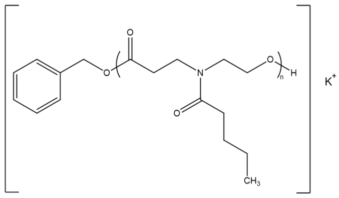

$$m/z = 0.108 \text{ kg/mol} + n \cdot 0.199 \text{ kg/mol} + 0.039 \text{ kg/mol}$$

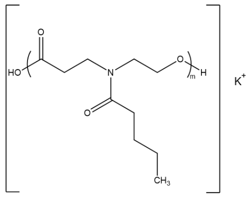

$$m/z = 0.018 \text{ kg/mol} + m \cdot 0.199 \text{ kg/mol} + 0.039 \text{ kg/mol}$$

Fig. S15 MALDI-ToF mass spectrum of purified **POxP$_{But}$** (DCTB, KTFA).

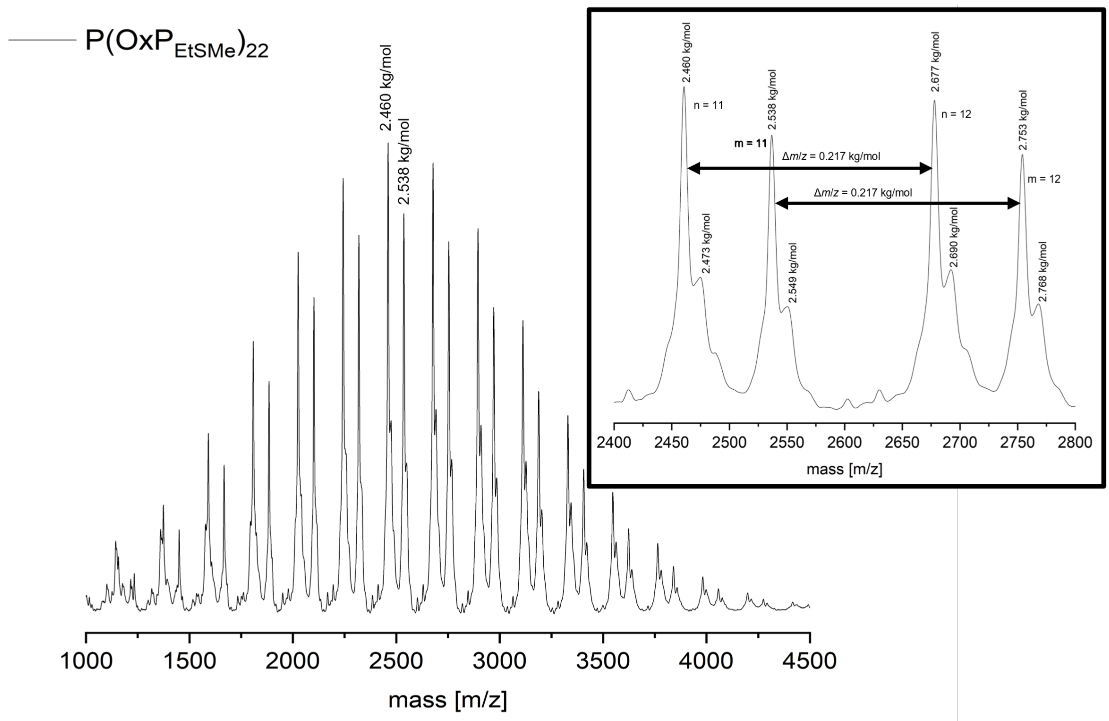

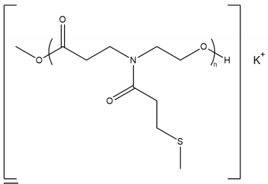

m/z = 0.032 kg/mol + n · 0.217 kg/mol + 0.039 kg/mol

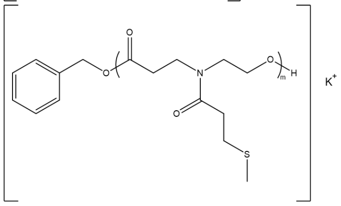

m/z = 0.108 kg/mol + m · 0.217 kg/mol + 0.039 kg/mol

Fig. S16 MALDI-ToF mass spectrum of purified **POxP$_{EtSMe}$** (DCTB, KTFA).

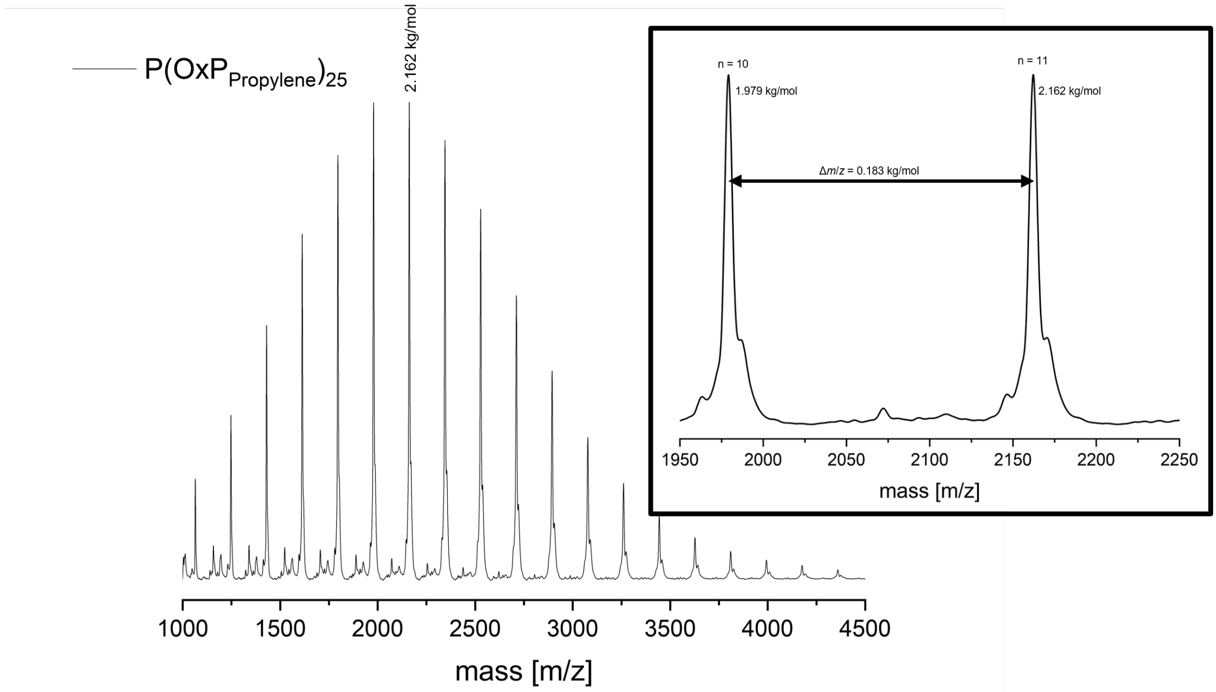

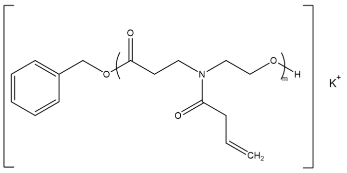

m/z = 0.108 kg/mol + m · 0.183 kg/mol + 0.039 kg/mol

Fig. S17 MALDI-ToF mass spectrum of purified **POxP$_{Propylene}$** (DCTB, KTFA).

Degradation

A

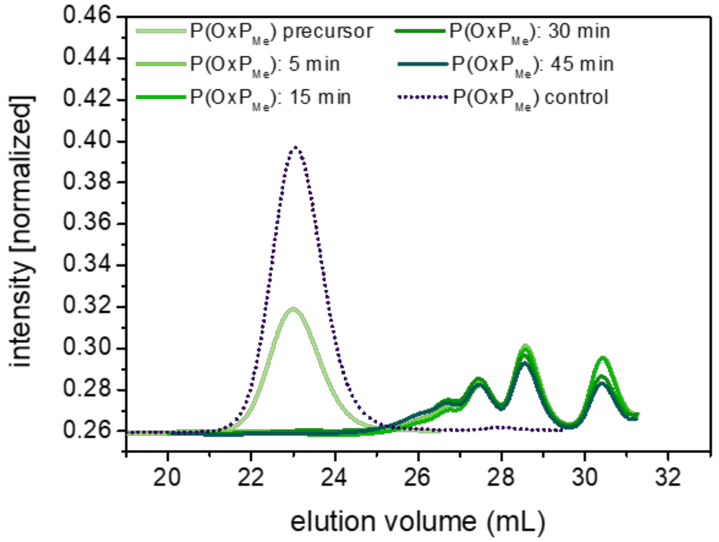

B

P(OxP$_{Me}$)	$\overline{Mn}_{,SEC}$ (g/mol)	$\overline{Mw}_{,SEC}$ (g/mol)
Precursor	3 200	3 500
5 min	90	190
15 min	80	180
30 min	110	230
45 min	110	260
Control	3 100	3 400

Fig. S18 P(OxP$_{Me}$) degradation under accelerated conditions: [P(OxP$_{Me}$)]= 10 mg/mL, [NaOH]= 0.005 M, MeOH, 25 °C. A) SEC elution traces (DMF, standard: PMMA). B) Table of the $\overline{Mn}_{,SEC}$ and $\overline{Mw}_{,SEC}$ values.

A

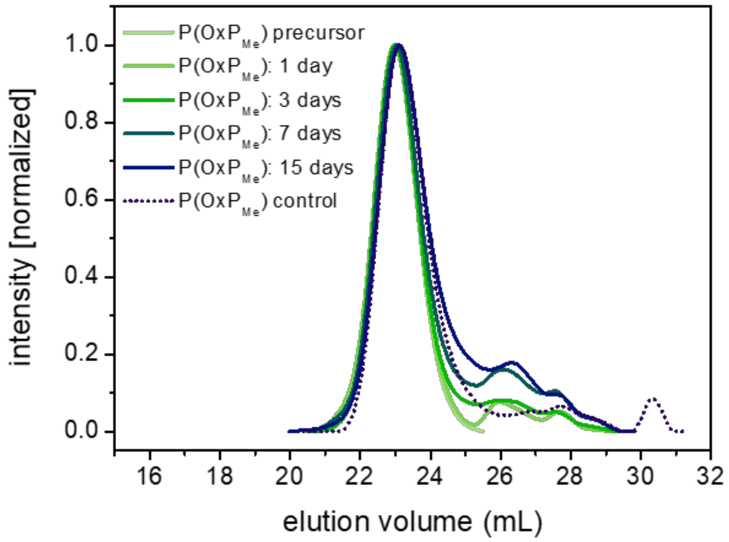

B

P(OxP$_{Me}$)	$\overline{Mn}_{,SEC}$ (g/mol)	$\overline{Mw}_{,SEC}$ (g/mol)
Precursor	3 200	3 500
1 day	3 200	3 600
3 days	1 700	3 100
7 days	1 200	2 900
15 days	1 200	2 700
Control	1 300	2 900

Fig. S19 P(OxP$_{Me}$) degradation under enzymatic conditions: [P(OxP$_{Me}$)]= 10 mg/mL, [LPC]= 100 U/mL, PBS, 37 °C. A) SEC elution traces (DMF, standard: PMMA). B) Table of the $\overline{Mn}_{,SEC}$ and $\overline{Mw}_{,SEC}$ values.

Table S1: $\overline{\mathbf{Mn}}_{,\mathrm{SEC}}$ and $\overline{\mathbf{Mw}}_{,\mathrm{SEC}}$ values of the $P(OxP_{Me})$ degradation under enzymatic conditions: $[P(OxP_{Me})]$= 5 mg/mL, [LPC]= 100 U/mL, PBS, 37 °C.

$P(OxP_{Me})$	$\overline{\mathbf{Mn}}_{,\mathrm{SEC}}$ (g/mol)	$\overline{\mathbf{Mw}}_{,\mathrm{SEC}}$ (g/mol)
Precursor	3 200	3 500
1 day	1 800	3 200
3 days	670	1 900
7 days	860	2 200
15 days	540	1 400
Control	1 300	2 600

A

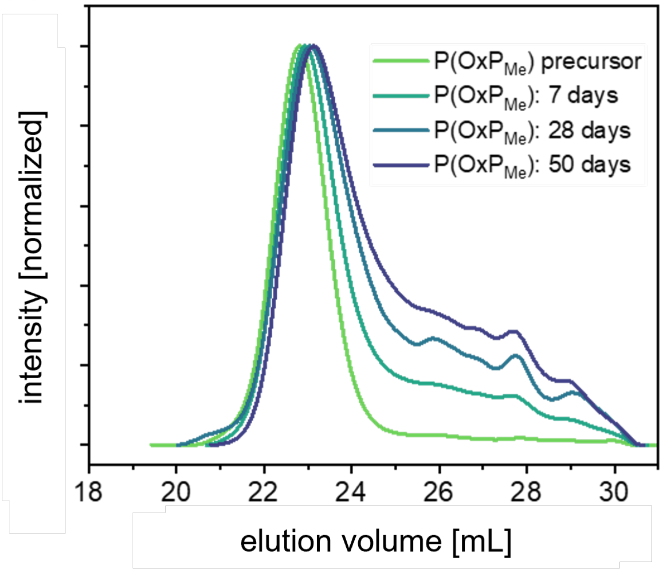

B

$P(OxP_{Me})$	$\overline{\mathbf{Mn}}_{,\mathrm{SEC}}$ (g/mol)	$\overline{\mathbf{Mw}}_{,\mathrm{SEC}}$ (g/mol)
Precursor	3 600	3 900
7 days	780	2 800
28 days	590	2 400
50 days	530	2 100

Fig. S20: $P(OxP_{Me})$ degradation in PBS solution: $[P(OxP_{Me})]$= 10 mg/mL, 37 °C. a) SEC elution traces (DMF, standard: PMMA). b) Table of the $\overline{\mathbf{Mn}}_{,\mathrm{SEC}}$ and $\overline{\mathbf{Mw}}_{,\mathrm{SEC}}$ values.

3. Synthetic Procedure and Characterization for OxP Monomers and Catalyst

1-*tert*-Butoxycarbonyl-4-piperidone (P_{Boc})

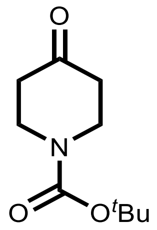

4-Piperidone monohydrate hydrochloride (20.0 g, 0.13 mol, 1.0 eq.) and K_2CO_3 (21.6 g, 0.16 mol, 1.2 eq.) were dissolved in water (150 mL). Boc_2O (31.3 g, 0.14 mmol, 1.1 eq.) was dissolved in THF (50 mL) and added dropwise to the aqueous solution under stirring at ambient temperature. After 16 h the yellow solution was extracted three times with Et_2O. The combined organic layers were dried over Na_2SO_4, filtered and the solvent removed under reduced pressure.

Yield: 25.9g (0.13 mol, quant.), colorless solid.

MF $C_{10}H_{17}NO_3$ MW 199.2500 g/mol [199.1208].

^{1}H NMR (300 MHz, $CDCl_3$, 294 K) δ / ppm = 3.71 (t, J = 6.3 Hz, 4H, 2x CH_2N), 2.43 (t, J = 6.2 Hz, 4H, 2x CH_2CO), 1.47 (s, 9H, CH_3).

4-*tert*-Butoxycarbonyl-1,4-oxazepan-7-one (OxP_{Boc})

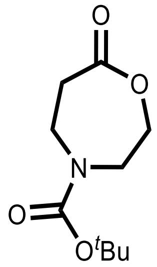

m-CPBA (43.8 g, 0.20 mol, 1.5 eq.) was dissolved in DCM (200 mL). Under cooling $\mathbf{P_{Boc}}$ (25.9 g, 0.13 mol, 1.0 eq.) dissolved in DCM (50 mL) was added dropwise. After full addition the cooling was removed, and the reaction stirred at room temperature for 16 h. The formed colorless precipitation was removed and a second portion of *m*-CPBA (9.0 g,0.04 mol, 0.3 eq.) was added to the reaction solution. After stirring at room temperature for 16 h the formed colorless precipitation was removed. Saturated sodium thiosulfate solution was added stirred for 30 min. The organic layer was separated, washed with saturated $NaHCO_3$-solution and water, dried over Na_2SO_4 and filtered. After removing the solvent under reduced pressure, the crude product was obtained as colorless, slightly yellow solid. Purification by silica gel chromatography (CH:EA=1:1) yield to the desired product.

Yield: 17.9 g (0.08 mol, 64%.), colorless solid.

MF $C_{10}H_{17}NO_4$ MW 215.2490 g/mol [215.1158].

R_F: 0.50 (SiO_2, CH:EA=1:1)

HR-TOF-MS (ESI, pos.), m/z: $[M+H]^+$ 238.1049 (calc. 238.1050), $[2M+MeOH+Na]^+$ 485.2466 (calc. 485.2470).

^1H NMR (400 MHz, $CDCl_3$, 294 K) δ / ppm = 4.26–4.24 (m, 2H, $COOCH_2$), 3.78–3.76 (m, 2H, $COOCH_2CH_2N$), 3.67–3.64 (m, 2H, NCH_2CH_2COO), 2.81–2.79 (m, 2H, CH_2COO), 1.47 (s, 9H, CH_3).

^{13}C NMR (101 MHz, $CDCl_3$, 294 K) δ / ppm = 173.97 (C-7), 154.45 ($C=O^{Boc}$), 81.14 (C_q^{Boc}), 69.57 (C-2), 47.52 (C-3), 40.91 (C-5), 37.63 (C-6), 28.44 (CH_3^{Boc}).

1,4-Oxazepan-7-one trifluoroacetate salt (OxP_{TFA})

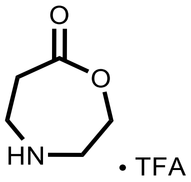

OxP_{Boc} (5.0 g, 0.02 mol, 1.0 eq.) and a solution of TFA in DCM (50% v/v, 25 mL) were stirred 45 min at room temperature. The solvent was evaporated under reduced pressure, yielding a yellow oil. Suspending in DCM and two times precipitation in cold Et_2O leading to a colorless solid. The supernatant was removed and the solid dried under reduced pressure.

Yield: 5.1 g (0.02 mmol, 95%), colorless solid.

MF $C_7H_{10}F_3NO_4$	MW g/mol $229.1552_{TFA\text{-}salt}$	$[229.0562]_{TFA\text{-}salt}$.
	MW g/mol $115.1320_{1,4\text{-}Oxazepan\text{-}7\text{-}one}$	$[115.0633]_{1,4\text{-}Oxazepan\text{-}7\text{-}one}$

HR-TOF-MS (ESI, pos.), m/z: $[M+H]^+$ 116.0709 (calc. 116.0706).

^1H NMR (400 MHz, DMSO-d_6, 294 K) δ/ppm = 4.46–4.44 (m, 2H, $COOCH_2$), 3.44–3.42 (m, 2H, $COOCH_2CH_2N$), 3.33–3.30 (m, 2H, NCH_2CH_2COO), 2.98–2.95 (m, 2H, CH_2COO).

^{13}C NMR (101 MHz, $CDCl_3$, 294 K) δ / ppm = 172.40 (C-7), 63.92 (C-2), 46.47 (C-3), 39 (C-5, under DMSO-signal), 31.81 (C-6).

Acylation of 1,4-oxazepan-7-one trifluoroacetate salt (OxP_{TFA}) towards 4-acylated-1,4-oxazepan-7-ones (GEP-1):

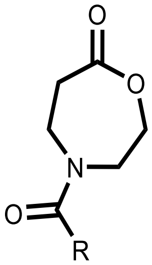

OxP_{TFA} (1.0 eq.) and K_2CO_3 (3.0 eq.) were stirred in DCM at room temperature under argon atmosphere. The corresponding acyl chloride (2.0 eq.) was added, and the reaction stirred for 16 h. The heterogeneous mixture was filtered, and the solvent removed under reduced pressure to obtain the crude material. The crude material was purified by silica gel chromatography.

4-Acetyl-1,4-oxazepan-7-one (OxP$_{Me}$)

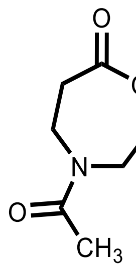

The compound was synthesized according to **GEP-1** using **OxP$_{TFA}$** (2.0 g, 8.73 mmol, 1.0 eq.), K_2CO_3 (3.6 g, 26.18 mmol, 3.0 eq.), acetyl chloride (1.4 g, 17.46 mmol, 2.0 eq.) and DCM (40 mL). The crude material was purified by silica gel chromatography (DCM:MeOH=20:1).

Yield: 1.0 g (6.59 mmol, 58%), colorless solid.

MF $C_7H_{11}NO_3$ MW 157.1690 g/mol [157.0739].

R_F: 0.22 (SiO_2, DCM:MeOH=20:1)

HR-TOF-MS (ESI, pos.), m/z: $[M+H]^+$ 158.0812 (calc. 158.0812), $[M+Na]^+$ 180.0631 (calc. 180.0631).

^{1}H NMR (400 MHz, $CDCl_3$, 294 K) δ/ppm = 4.31–4.25 (m, 2H, COOCH_2), 3.96–3.66 (m, 4H, N(CH_2)$_2$), 2.87–2.80 (m, 2H, CH_2COO), 2.18-2.15 (d, J = 11.8 Hz, 3H, CH_3).

^{13}C NMR (101 MHz, $CDCl_3$, 294 K) δ / ppm = 173.52 (C-7^b), 173.11 (C-7^a), 169.43 (C=O^{Acetyl}), 69.51 (C-2^a), 68.94 (C-2^b), 50.23 (C-3^b), 45.51 (C-3^a), 43.65 (C-5^a), 38.67 (C-5^b), 37.89 (C-6^a), 36.87 (C-6^b), 21.87 (CH_3^b), 21.53 (CH_3^a).

4-Pentanoyl-1,4-oxazepan-7-one (OxP$_{But}$)

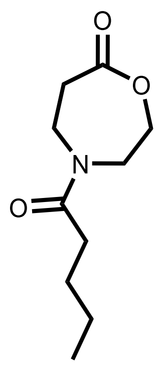

The compound was synthesized according to **GEP-1** using **OxP$_{TFA}$** (1.0 g, 4.36 mmol, 1.0 eq.), K_2CO_3 (1.8 g, 13.09 mmol, 3.0 eq.), pentanoyl chloride (1.1 g, 8.73 mmol, 2.0 eq.) and DCM (20 mL). The crude material was purified by silica gel chromatography (CH:EA=1:3).

Yield: 517 mg (2.59 mmol, 59%), colorless oil.

MF $C_{10}H_{17}NO_3$ MW 199.2500 g/mol [199.1208].

R_F: 0.30 (SiO_2, CH:EA=1:3)

HR-TOF-MS (ESI, pos.), m/z: $[M+H]^+$ 200.1283 (calc. 200.1281), $[M+Na]^+$ 222.1104 (calc. 222.1100), $[M+K]^+$ 238.0841 (calc. 238.0840)

^{1}H NMR (400 MHz, $CDCl_3$, 294 K) δ/ppm = 4.27–4.23 (m, 2H, COOCH_2), 3.94–3.65 (m, 4H, N(CH_2)$_2$), 2.84–2.78 (m, 2H, CH_2COO), 2.39–2.31 (m, 2H, CH_2^{α}), 1.64–1.56 (p, J = 7.6 Hz, 2H, CH_2^{β}), 1.35 (h, J = 7.5 Hz, 2H, CH_2^{γ}), 0.92 (t, J = 7.3 Hz, 3H, CH_3).

^{13}C NMR (101 MHz, $CDCl_3$, 294 K) δ / ppm = 173.65 (C-7^b), 173.23 (C-7^a), 172.11 (C=O^{Acetyl}), 69.67 (C-2^a), 69.10 (C-2^b), 49.40 (C-3^b), 45.65 (C-3^a), 42.83 (C-5^a), 38.79 (C-5^b), 38.04 (C-6^a), 36.99 (C-6^b), 33.40 (C$^{\alpha,b}$), 33.08 (C$^{\alpha,a}$), 27.47 (C$^{\beta}$), 22.64 (C$^{\gamma}$), 13.99 (CH_3).

4-(3-Methylthio)propanoyl-1,4-oxazepan-7-one (OxP$_{EtSMe}$)

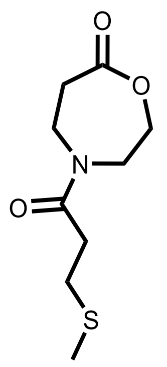

The compound was synthesized according to **GEP-1** using **OxP$_{TFA}$** (100 mg, 0.44 mmol, 1.0 eq.), K_2CO_3 (181 mg, 1.31 mmol, 3.0 eq.), 3-(methylthio)propionyl chloride (121 mg, 0.87 mmol, 2.0 eq.) and DCM (2 mL). The crude material was purified by silica gel chromatography (CH:EA=1:3).

Yield: 45 mg (0.21 mmol, 47%), colorless oil.

MF $C_9H_{15}NO_3S$ MW 217.2830 g/mol [217.0773].

R_F: 0.20 (SiO_2, CH:EA=1:3)

HR-TOF-MS (ESI, pos.), m/z: $[M+H]^+$ 218.0841 (calc. 218.0846), $[M+Na]^+$ 240.0664 (calc. 240.0665), $[M+K]^+$ 256.0402 (calc. 256.0405).

^{1}H NMR (400 MHz, $CDCl_3$, 294 K) δ/ppm = 4.32–4.25 (m, 2H, COOCH_2), 3.97–3.67 (m, 4H, N(CH_2)$_2$), 2.87–2.79 (m, 4H, CH_2COO, CH_2^β), 2.70-2.61 (m, 2H, CH_2^α) 2.13 (s, 3H, CH_3).

^{13}C NMR (101 MHz, $CDCl_3$, 294 K) δ / ppm = 173.45 (C-7^b), 173.07 (C-7^a), 170.28 (C=O^{Acetyl}), 69.47 (C-2^a), 68.90 (C-2^b), 49.29 (C-3^b), 45.85 (C-3^a), 42.74 (C-5^a), 38.96 (C-5^b), 37.85 (C-6^a), 36.84 (C-6^b), 33.53 (C$^{\alpha,a}$), 33.18 (C$^{\alpha,b}$), 29.76 (C$^\beta$), 16.14 (CH_3).

4-(But-3-enoyl)-1,4-oxazepan-7-one (OxP$_{Propylene}$)

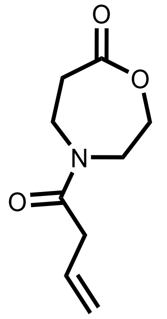

3-Butenoic acid (281 mg, 3.27 mmol, 1.0 eq.) and thionyl chloride (0.5 g, 4.25 mmol, 1.3 eq.) were stirred at 70°C for 1 h to form 3-butenoyl chloride. Evolving HCl-gas was trapped in a water filled beaker connected to the apparatus. Residues of thionyl chloride were removed under removed pressure, leaving a yellow to brown liquid.

OxP$_{TFA}$ (0.75 g, 3.27 mmol, 1.0 eq.) and K_2CO_3 (1.4 g, 9.82 mmol, 3.0 eq.) were stirred in DCM (15 mL) at room temperature under argon atmosphere. Freshly prepared 3-butenoyl chloride was added

dropwise via syringe. The reaction stirred for 16 h at room temperature. The heterogeneous mixture was filtered, and the solvent removed under reduced pressure to obtain the crude product. The crude material was purified by silica gel chromatography.

Yield: 0.4 g (2.24 mmol, 69%), colorless oil.

MF $C_9H_{13}NO_3$ MW 183.2070 g/mol [183.0895].

HR-TOF-MS (ESI, pos.), m/z: $[M+H]^+$ 184.0966 (calc. 184.0968), $[M+Na]^+$ 206.0785 (calc. 206.0787)

R_F: 0.21 (SiO_2, CH:EA=1:3)

^1H NMR (400 MHz, $CDCl_3$, 294 K) δ/ppm = 5.99–5.87 (m, 1H, C*H*), 5.23–5.15 (m, 2H, $CHCH_2$), 4.27–4.25 (m, 2H, $COOCH_2$), 3.96–3.66 (m, 4H, $N(CH_2)_2$), 3.23–3.18 (m, 2H, CH_2^{α}), 2.85–2.79 (m, 2H, CH_2COO).

^{13}C NMR (101 MHz, $CDCl_3$, 294 K) δ / ppm = 173.55 (C-7^b), 173.13 (C-7^a), 169.95 (C=O^{Acetyl}), 169.85 (C=O^{Acetyl}), 130.93 (C$^{\beta}$), 118.75 (C$^{\gamma}$), 69.48 (C-2^a), 69.03 (C-2^b), 49.63 (C-3^b), 45.72 (C-3^a), 43.08 (C-5^a), 39.14 (C-5^b), 38.91 (C$^{\alpha}$), 38.84 (C$^{\alpha}$), 37.89 (C-6^a), 36.86 (C-6^b).

1-[3,5-bis(trifluoromethyl)phenyl]-3-cyclohexyl thiourea (TU)

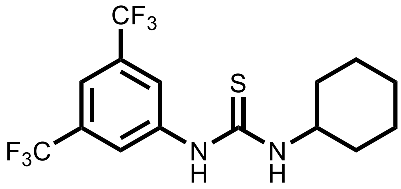

Cyclohexylamine (174 mg, 1.75 mmol, 1.0 eq.) was dissolved in DCM (1.3 mL) and stirred under cooling, using a water bath. 3,5-Bis-(trifluoromethyl)-phenyl isothiocyanate (476 mg, 1.75 mmol, 1.0 eq.) was added dropwise. After full addition the cooling was removed and stirred at room temperature for 5 minutes. The colorless precipitate was filtered, washed with *n*-pentane and dried under reduced pressure.

Yield: 512 mg (1.57 mmol, 90%), colorless solid.

MF $C_{15}H_{16}F_6N_2S$ MW 370.3574 g/mol [370.0938].

HR-TOF-MS (ESI, pos.), m/z: $[M+H]^+$ 371.1011 (calc. 371.1011).

^1H NMR (400 MHz, $CDCl_3$, 294 K) δ/ppm = 7.89 (bs, 1H, Ar-N*H*), 7.75–7.71 (m, 3H, Ar-*H*), 6.00 (bs, 1H, Cy-N*H*), 4.19 (bs, 1H, NCy-C*H*), 2.11–2.06 (m, 2H, Cy-*H*), 1.74–1.60 (m, 3H, Cy-*H*), 1.50–1.36 (m, 2H, Cy-*H*), 1.28–1.13 (m, 3H, Cy-*H*).

4. Synthetic Procedure and Characterization for POxP Homopolymers

General procedure for the homopolymerization of *N*-acylated-1,4-oxazepan-7-ones (GEP-2)

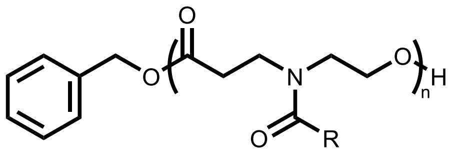

All polymerizations were carried out in an argon-filled glovebox (*MBraun* UNILAB, <0.1 ppm of O_2 and <0.1 ppm of H_2O) at room temperature in 1.5 mL glass vials equipped with magnetic stir bar and screw cap. Monomer (30 eq.) was dissolved in dry solvent (DCM or toluene) in a 1.5 mL glass vial. The organocatalysts DBU (3 eq.), TU (3 eq.) and the initiator BnOH (1 eq.) were dissolved in dry solvent (DCM or toluene). The amount of solvent is determined so that $[\text{Monomer}]_0$=1 M. After 5 minutes the initiator-catalyst solution was added to the monomer solution to start the polymerization. The mixture was stirred 80–90 minutes and quenched by the addition of benzoic acid. The polymer was precipitated two times in cold Et_2O and dried under reduced pressure.

Poly(4-acetyl-1,4-oxazepan-7-one) (P(OxP_{Me}))

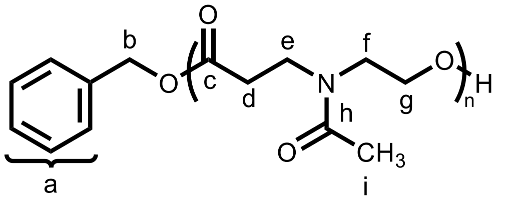

The polymerization was conducted as described in **GEP-2**, using OxP_{Me} (205 mg, 1.31 mmol, 30 eq.), DBU (19 µL, 20 mg, 0.13 mmol, 3 eq.), TU (48 mg, 0.13 mmol, 3 eq.), BnOH (4.5 µL, 4.7 mg, 0.04 mmol, 1 eq.) and DCM (1281 µL).

Find more data in Table 1, entry 3.

^{1}H NMR (400 MHz, $CDCl_3$, 294 K) δ/ppm = 7.37–7.33 (m, 5H, CH^{a}), 5.11 (d, 2H, *J* = 8.8 Hz, CH_2^{b}) 4.22–4.19 (m, 2H, CH_2^{g}), 3.64–3.54 (m, 4H, CH_2^{e}, CH_2^{f}), 2.65–2.60 (m, 2H, CH_2^{d}), 2.13–2.10 (m, 3H, CH_3^{i}).

^{13}C NMR (101 MHz, $CDCl_3$, 294 K) δ / ppm = 172.00 (C^{c}), 171.33 (C^{h}), 171.24 (C^{h}), 170.96 (C^{h}), 170.80 (C^{h}), 128.73 (C^{a}), 128.61 (C^{a}), 128.47 (C^{a}), 66.62 (C^{b}), 62.45 (C^{g}), 61.99 (C^{g}), 48.19 (C^{f}), 48.06 (C^{f}), 45.23 ($C^{e/f}$), 44.82 ($C^{e/f}$), 44.59 ($C^{e/f}$), 42.50 (C^{e}), 33.75 (C^{d}), 33.61 (C^{d}), 32.72 (C^{d}), 21.73 (C^{i}), 21.68 (C^{i}), 21.56 (C^{i}).

Poly(4-pentanoyl-1,4-oxazepan-7-one) (P(OxP$_{But}$))

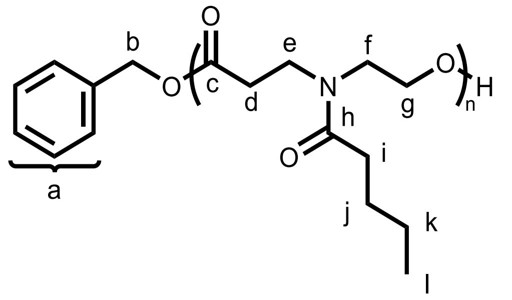

The polymerization was conducted as described in **GEP-2**, using OxP$_{But}$ (115 mg, 0.58 mmol, 30 eq.), DBU (9 µL, 9 mg, 0.06 mmol, 3 eq.), TU (21 mg, 0.06 mmol, 3 eq.), BnOH (2 µL, 2 mg, 0.02 mmol, 1 eq.) and DCM (568 µL).

Find more data in Table 1, entry 4 and 5.

^{1}H NMR (400 MHz, CDCl$_3$, 294 K) δ/ppm = 7.35–7.33 (m, 5H, CH^{a}), 5.11 (d, 2H, J = 9.4 Hz, CH_2^{b}) 4.20–4.17 (m, 2H, CH_2^{g}), 3.63–3.54 (m, 4H, CH_2^{e}, CH_2^{f}), 2.60–2.52 (m, 2H, CH_2^{d}), 2.32–2.29 (m, 2H, CH_2^{i}), 1.61–1.55 (m, 2H, CH_2^{j}), 1.37–1-32 (m, 2H, CH_2^{k}), 0.93–0.88 (m, 3H, CH_3^{l}).

^{13}C NMR (101 MHz, CDCl$_3$, 294 K) δ / ppm = 173.65 (C^{h}), 172.01 (C^{c}), 170.81 (C^{c}), 128.68 (C^{a}), 128.50 (C^{a}), 62.45 (C^{g}), 62.12 (C^{g}), 47.12 ($C^{e/f}$), 44.88 ($C^{e/f}$), 44.32 ($C^{e/f}$), 42.64 ($C^{e/f}$), 33.91 (C^{d}), 33.74 (C^{d}), 32.84 (C^{i}), 27.48 (C^{j}), 22.61 (C^{k}), 14.05 (C^{l}).

Poly(4-(3-methylthio)propanoyl-1,4-oxazepan-7-one) (P(OxP$_{EtSMe}$)

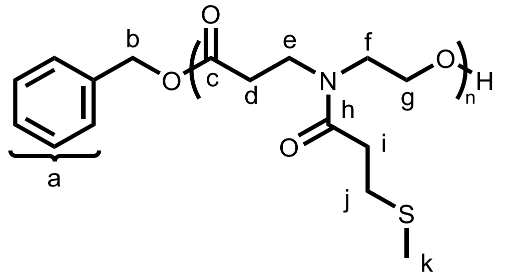

The polymerization was conducted as described in **GEP-2**, using OxP$_{EtSMe}$ (95 mg, 0.44 mmol, 30 eq.), DBU (7 µL, 7 mg, 0.04 mmol, 3 eq.), TU (16 mg, 0.04 mmol, 3 eq.), BnOH (1.5 µL, 1.6 mg, 0.014 mmol, 1 eq.) and DCM (430 µL).

Find more data in Table 1, entry 1.

^{1}H NMR (400 MHz, CDCl$_3$, 294 K) δ/ppm = 7.35–7.30 (m, 5H, CH^{a}), 5.10 (d, 2H, J = 9.9 Hz, CH_2^{b}) 4.21–4.18 (m, 2H, CH_2^{g}), 3.65–3.56 (m, 4H, CH_2^{e}, CH_2^{f}), 2.79–2.74 (m, 2H, CH_2^{j}), 2.66–2.62 (m, 4H, CH_2^{d}, CH_2^{i}), 2.12–2.10 (m, 3H, CH_3^{k}).

^{13}C NMR (101 MHz, CDCl$_3$, 294 K) δ / ppm = 171.97 ($C^{c/h}$), 171.89 ($C^{c/h}$), 171.73 ($C^{c/h}$), 170.84 ($C^{c/h}$), 170.73 ($C^{c/h}$), 128.77 (C^{a}), 128.68 (C^{a}), 128.55 (C^{a}), 128.39 (C^{a}), 66.57 (C^{b}), 62.35 (C^{g}), 62.01 (C^{g}), 47.05 ($C^{e/f}$), 46.95 ($C^{e/f}$), 44.97 ($C^{e/f}$), 44.76 ($C^{e/f}$), 44.25 ($C^{e/f}$), 43.16 ($C^{e/f}$), 42.69 ($C^{e/f}$), 33.77 ($C^{d/i}$), 33.60 ($C^{d/i}$), 33.33 ($C^{d/i}$), 33.23 ($C^{d/i}$), 32.69 ($C^{d/i}$), 32.62 ($C^{d/i}$), 29.67 (C^{j}), 16.10 (C^{k}).

Poly(4-(but-3-enoyl)-1,4-oxazepan-7-one) (P(OxP$_{Propylene}$))

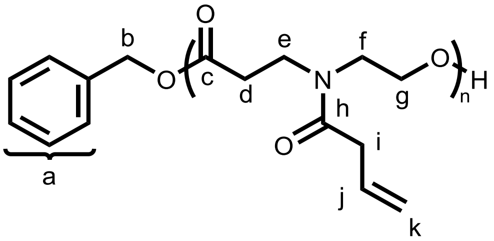

The polymerization was conducted as described in **GEP-2**, using OxP$_{Propylene}$ (107 mg, 0.59 mmol, 30 eq.), DBU (9 µL, 9 mg, 0.06 mmol, 3 eq.), TU (21 mg, 0.06 mmol, 3 eq.), BnOH (2 µL, 2 mg, 0.02 mmol, 1 eq.) and DCM (576 µL).

Find more data in Table 1, entry 2.

^{1}H NMR (400 MHz, $CDCl_3$, 294 K) δ/ppm = 7.35–7.33 (m, 5H, CH^a), 5.97–5.86 (m,1H, CH^j), 5.15–5.08 (m, 4H, CH_2^b, CH_2^k), 4.21–4.18 (m, 2H, CH_2^g), 3.63–3.54 (m, 4H, CH_2^e, CH_2^f), 3.19–3.10 (m, 2H, CH_2^i). 2.66–2.61 (m, 2H, CH_2^d).

^{13}C NMR (101 MHz, $CDCl_3$, 294 K) δ / ppm = 171.90 ($C^{c/h}$), 171.45 ($C^{c/h}$), 170.86 ($C^{c/h}$), 131.47 (C^j), 128.78 (C^a), 128.67 (C^a), 128.55 (C^a), 128.39 (C^a), 121.27 (C^k), 118.08 (C^k), 66.96 (C^b), 66.56 (C^b), 62.33 (C^g), 62.02 (C^g), 47.21 ($C^{e/f}$), 47.21 ($C^{e/f}$), 44.83 ($C^{e/f}$), 44.40 ($C^{e/f}$), 42.62 ($C^{e/f}$), 38.42 ($C^{e/f}$), 33.64 (C^i), 32.61 (C^d).

5. NMR Data of OxP monomers and catalyst

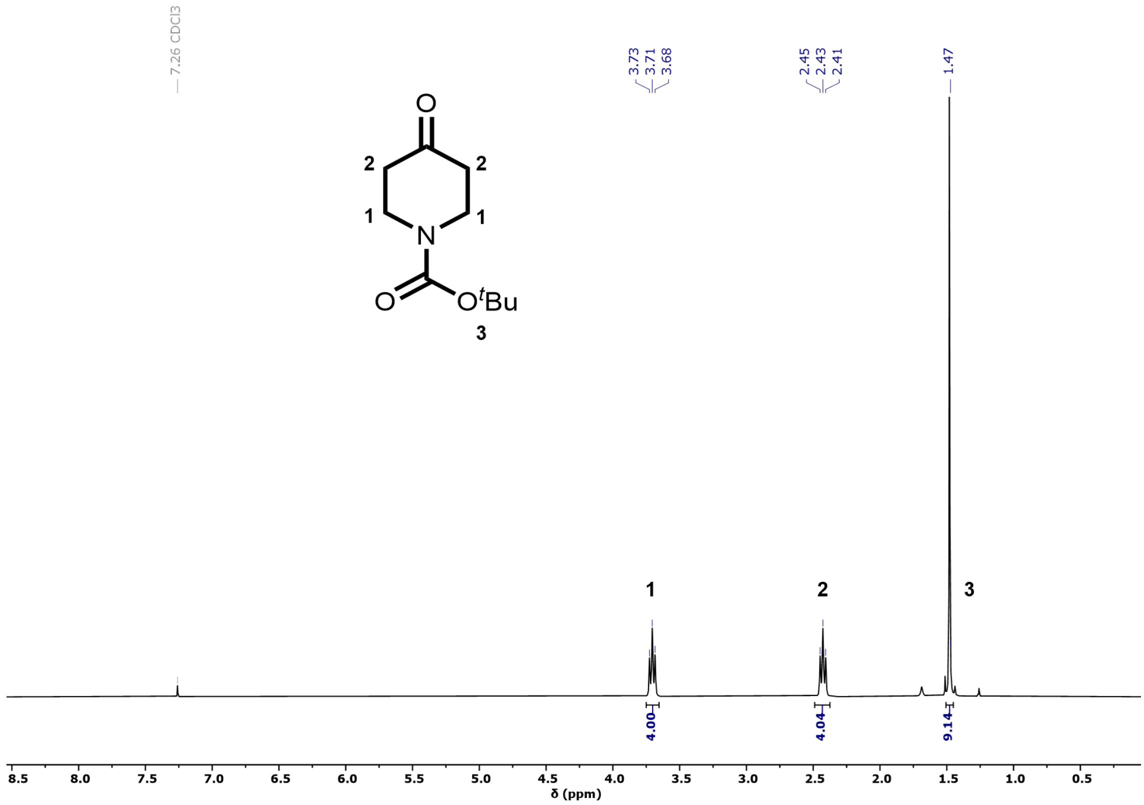

Fig. S21 ^{1}H NMR spectrum of 1-*tert*-Butoxycarbonyl-4-piperidone (**P$_{Boc}$**) (300 MHz, $CDCl_3$).

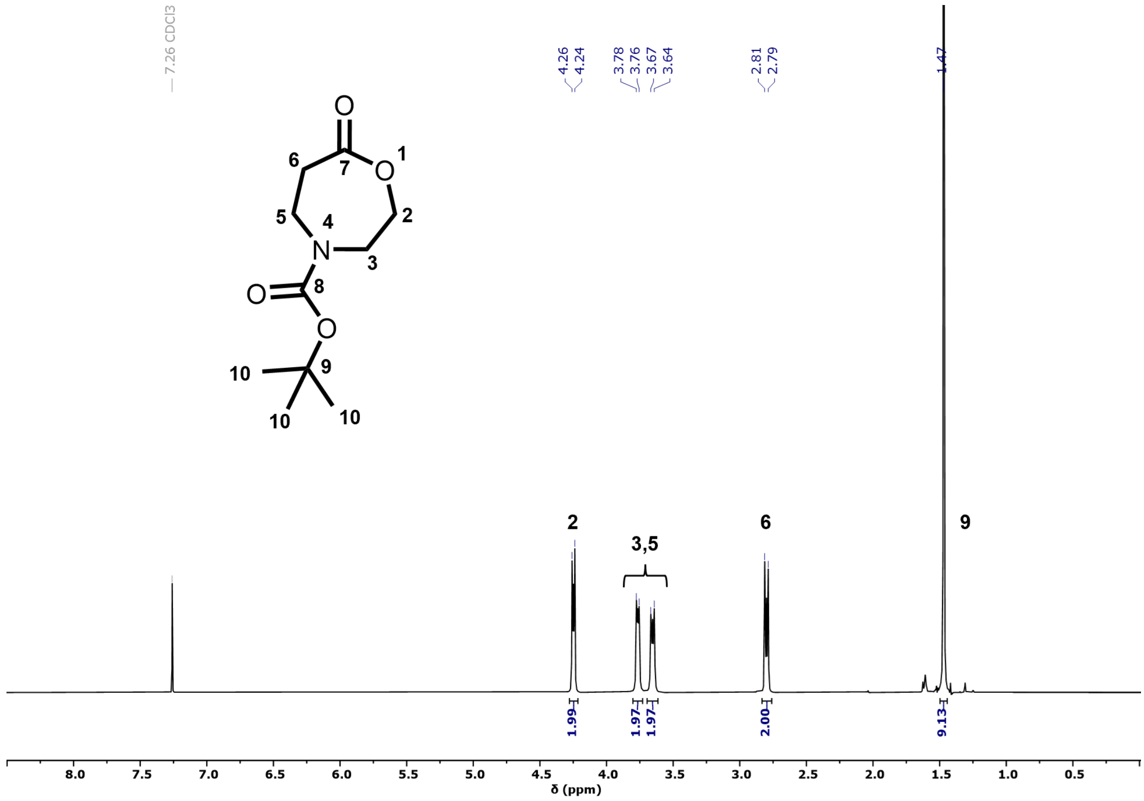

Fig. S22 ^{1}H NMR spectrum of 4-*tert*-Butoxycarbonyl-1,4-oxazepan-7-one (**OxP$_{Boc}$**) (400 MHz, $CDCl_3$).

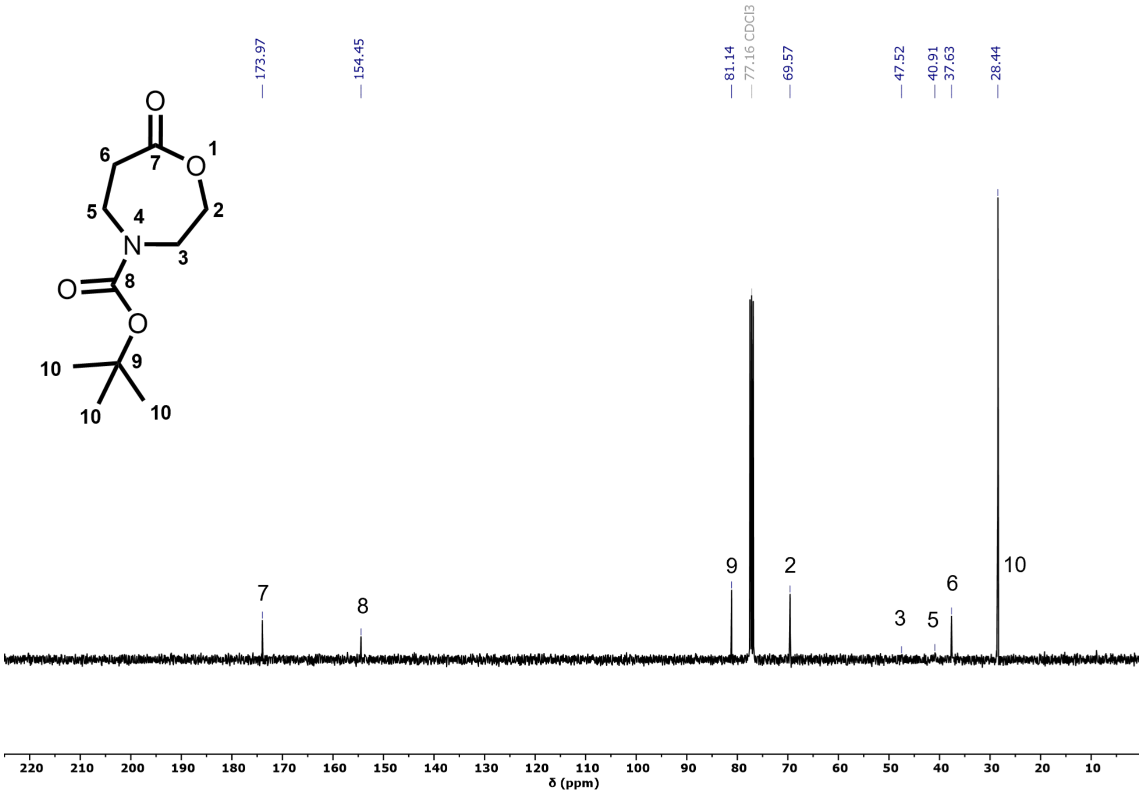

Fig. S23 ^{13}C NMR spectrum of 4-*tert*-Butoxycarbonyl-1,4-oxazepan-7-one (**OxP$_{Boc}$**) (400 MHz, $CDCl_3$).

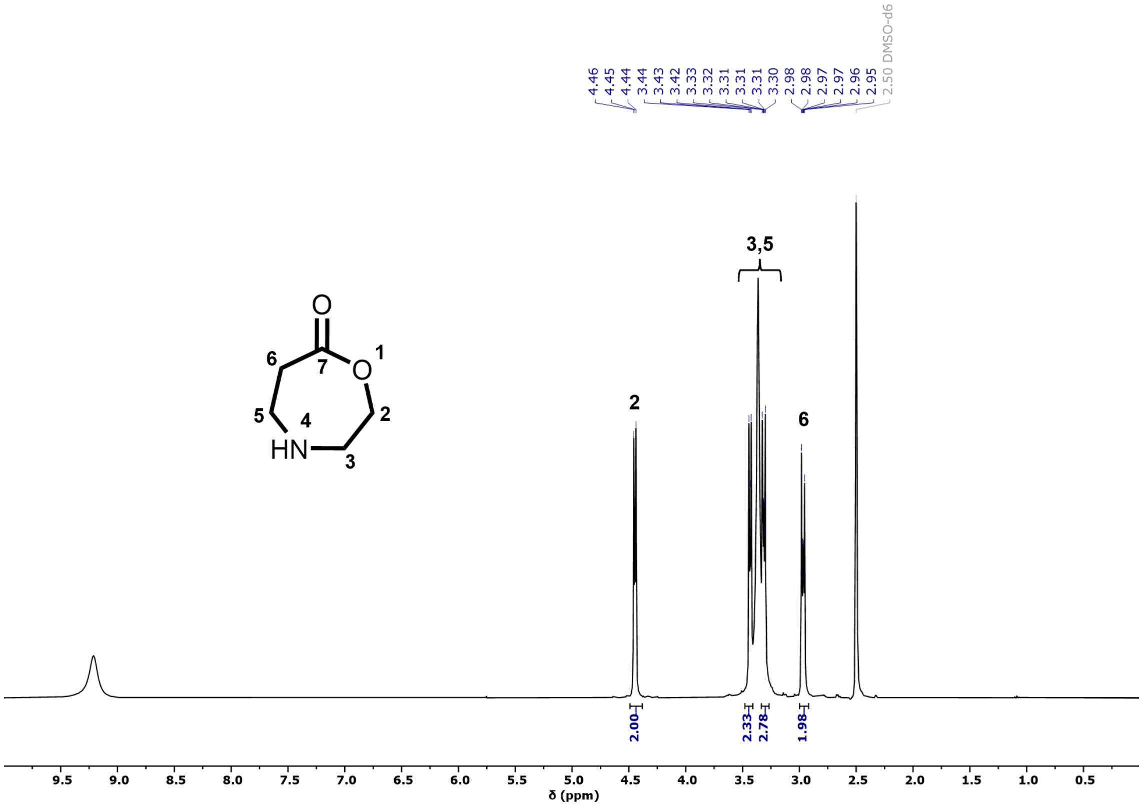

Fig. S24 ^{1}H NMR spectrum of 1,4-Oxazepan-7-one trifluoroacetate salt (**OxP$_{TFA}$**) (400 MHz, DMSO-d_6)

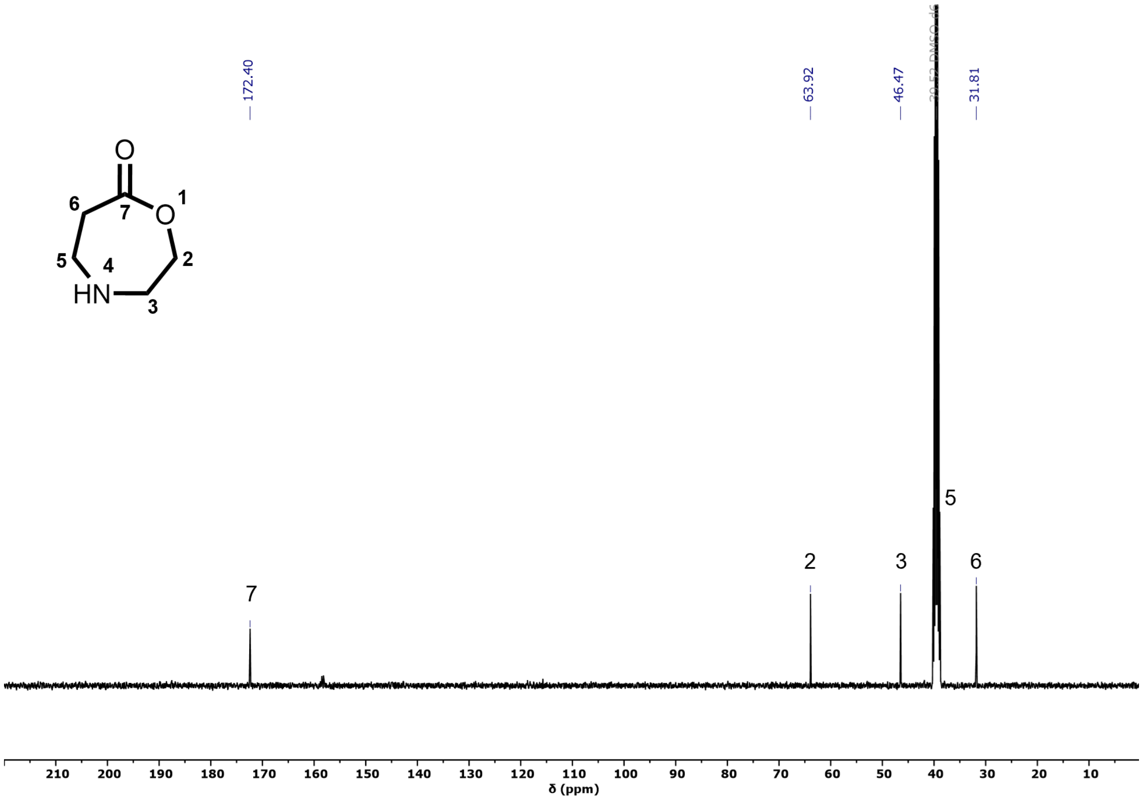

Fig. S25 ^{13}C NMR spectrum of 1,4-Oxazepan-7-one trifluoroacetate salt (**OxP$_{TFA}$**) (400 MHz, DMSO-d_6)

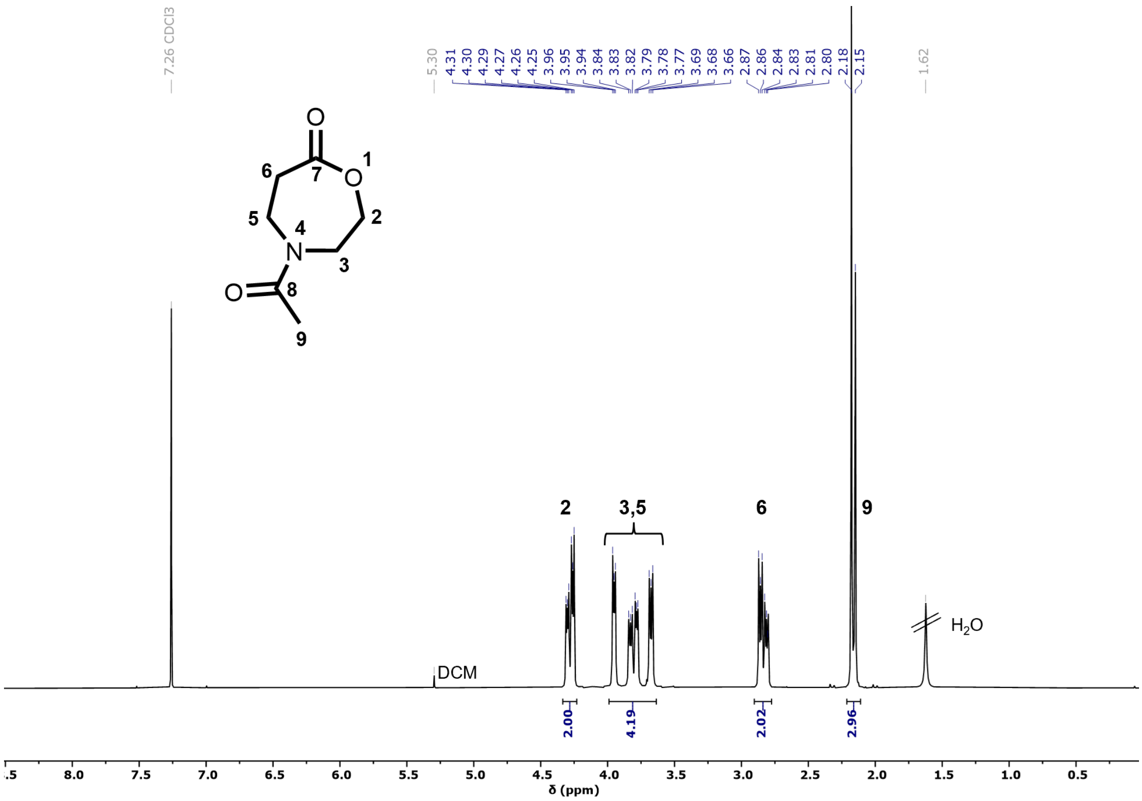

Fig. S26 ^{1}H NMR spectrum of 4-Acetyl-1,4-oxazepan-7-one (**OxP$_{Me}$**) (400 MHz, CDCl$_3$).

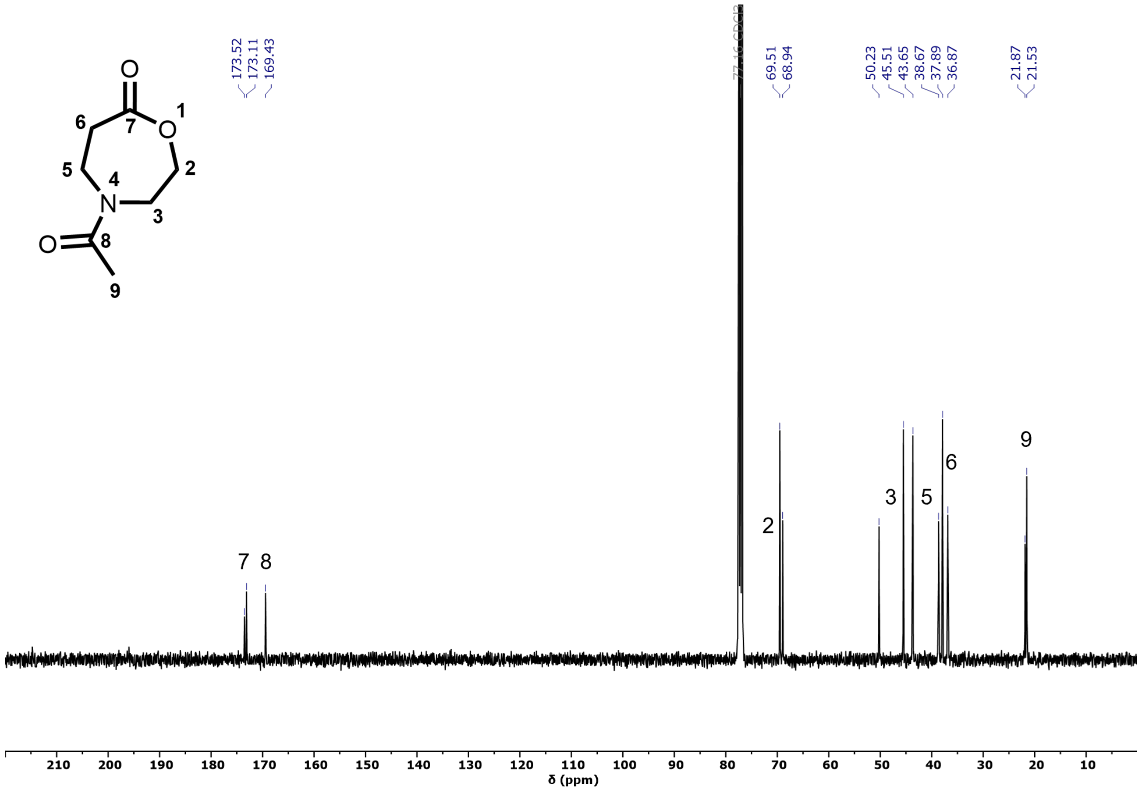

Fig. S27 ^{13}C NMR spectrum of 4-Acetyl-1,4-oxazepan-7-one (**OxP$_{\mathbf{Me}}$**) (400 MHz, CDCl$_3$).

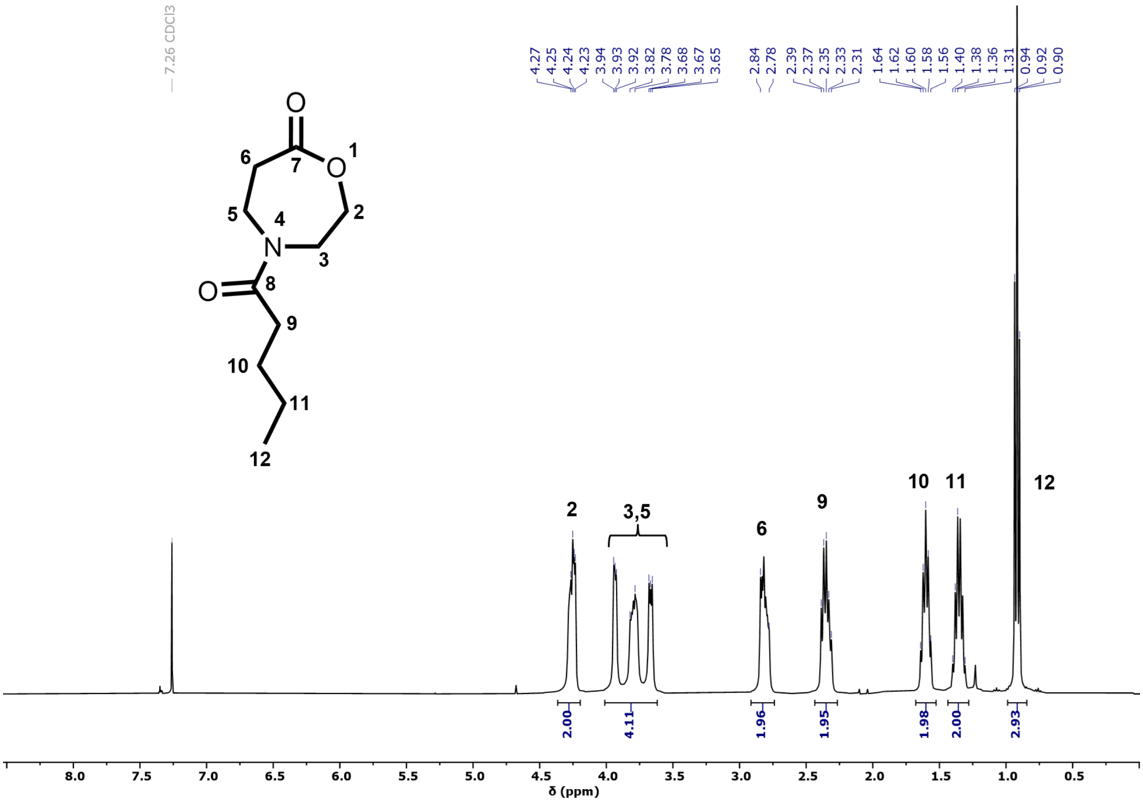

Fig. S28 ^{1}H NMR spectrum of 4-Pentanoyl-1,4-oxazepan-7-one (**OxP$_{\mathbf{But}}$**) (400 MHz, CDCl$_3$).

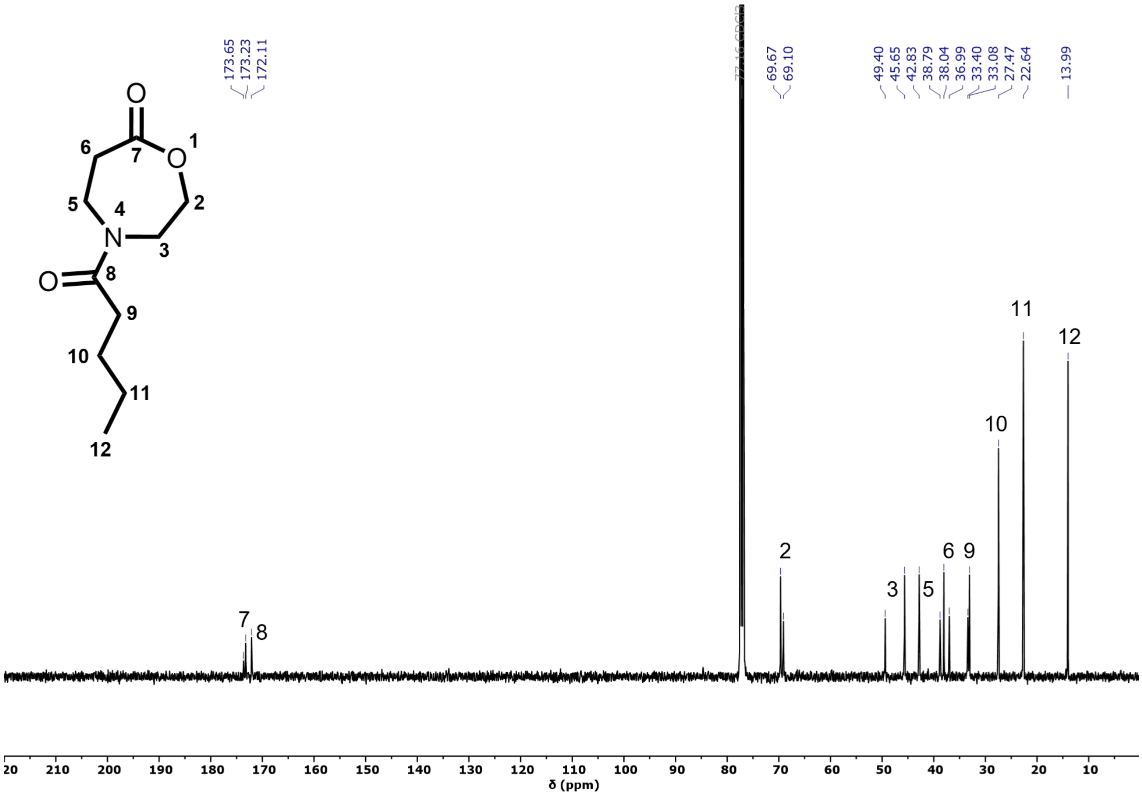

Fig. S29 ^{13}C NMR spectrum of 4-Pentanoyl-1,4-oxazepan-7-one (**OxP$_{But}$**) (400 MHz, CDCl$_3$).

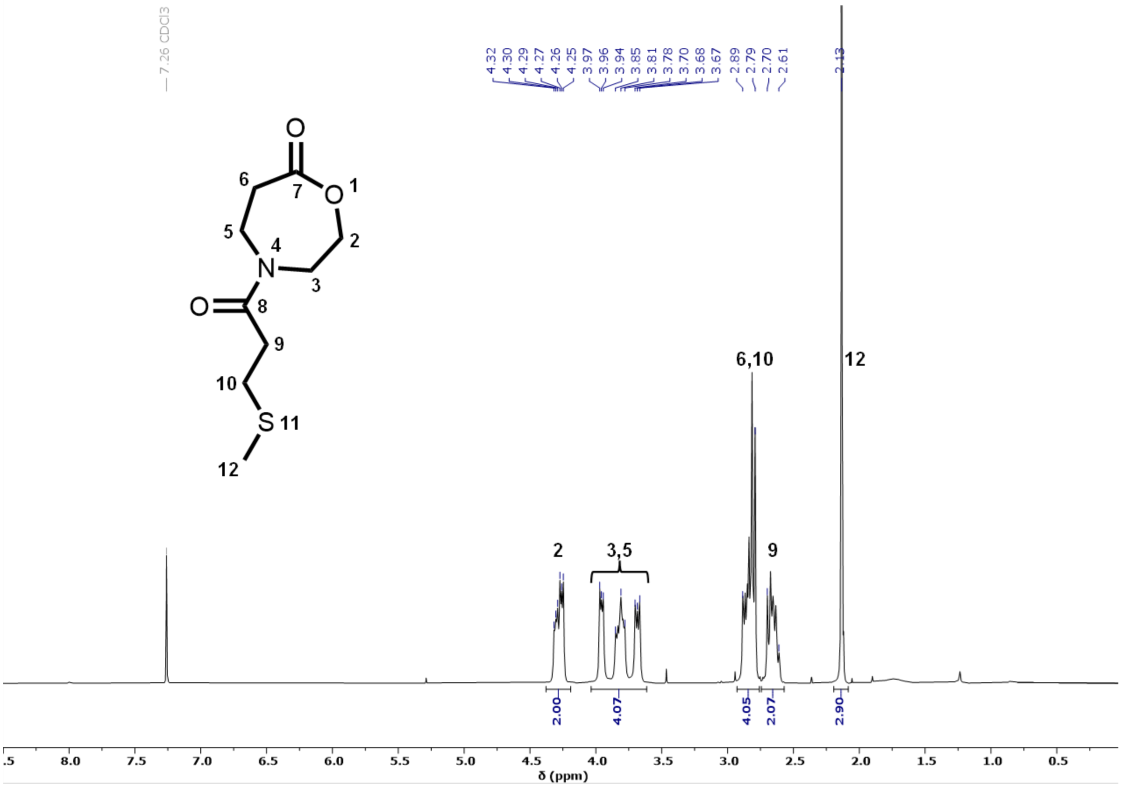

Fig. S30 ^{1}H NMR spectrum of 4-(3-Methylthio)propanoyl-1,4-oxazepan-7-one (**OxP$_{EtSMe}$**) (400 MHz, CDCl$_3$).

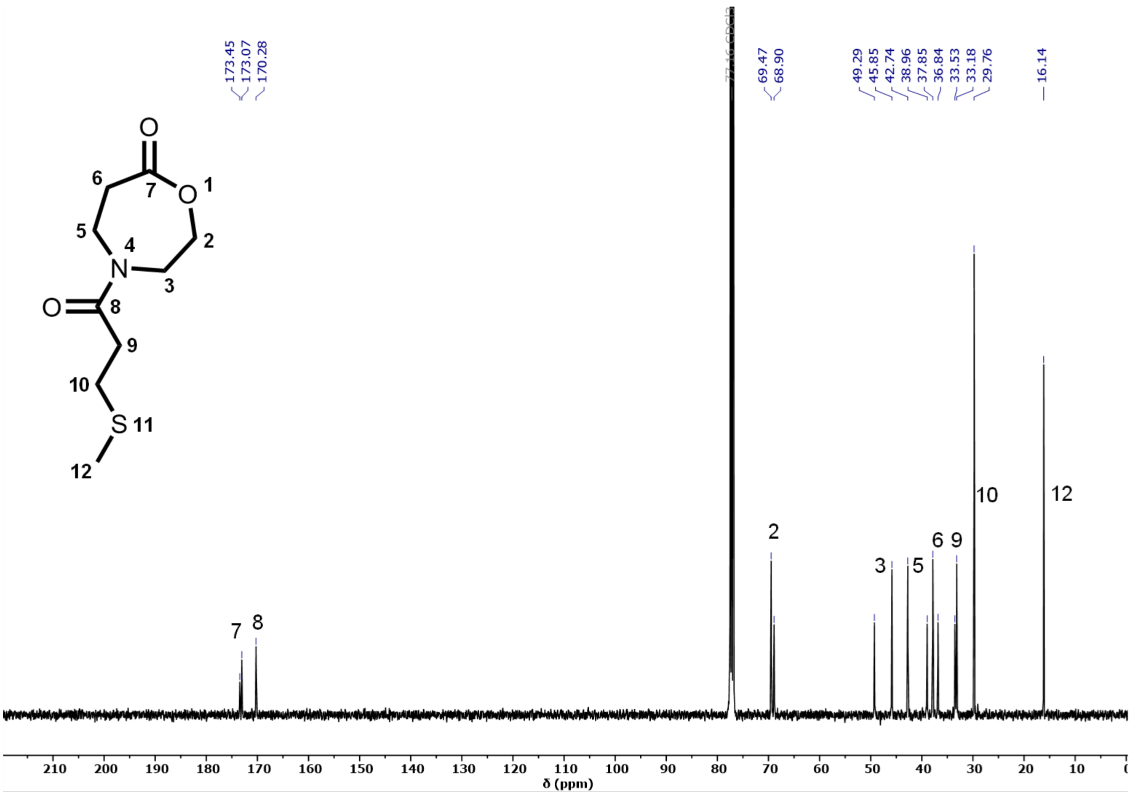

Fig. S31 ^{13}C NMR spectrum of 4-(3-Methylthio)propanoyl-1,4-oxazepan-7-one (**OxP$_{EtSMe}$**) (400 MHz, CDCl$_3$).

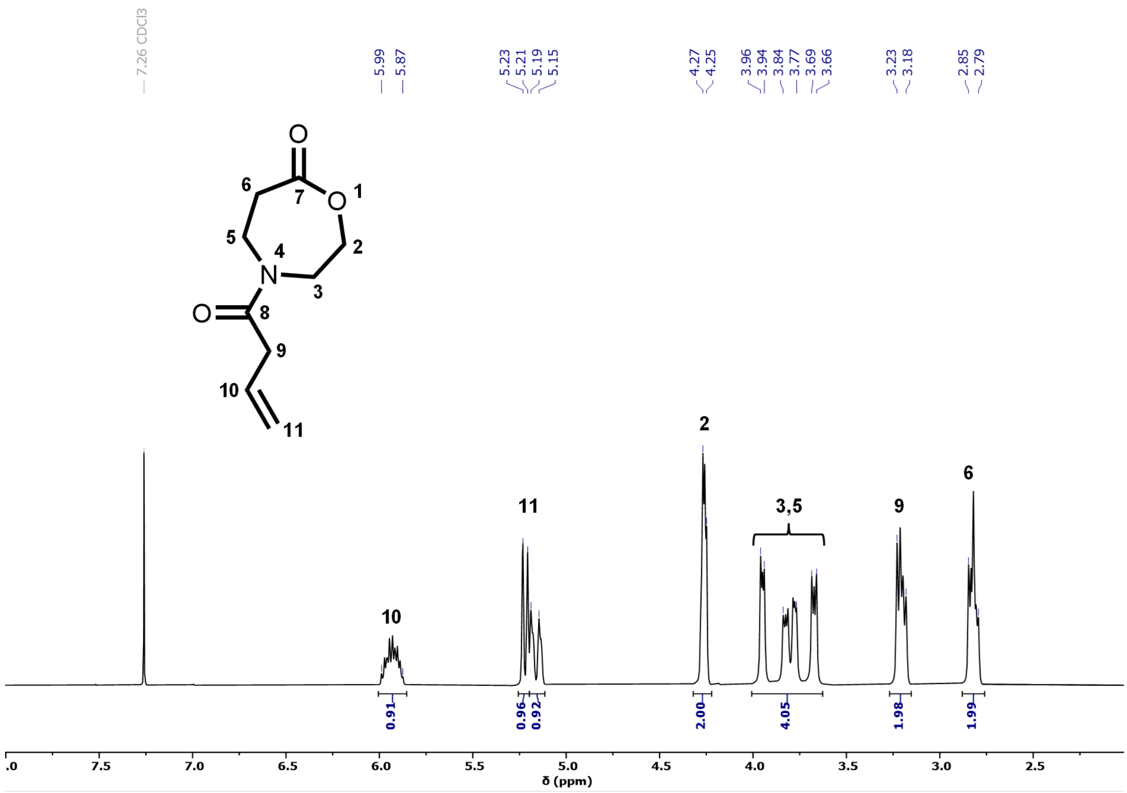

Fig. S32 ^{1}H NMR spectrum of 4-(But-3-enoyl)-1,4-oxazepan-7-one (**OxP$_{Propylene}$**) (400 MHz, CDCl$_3$).

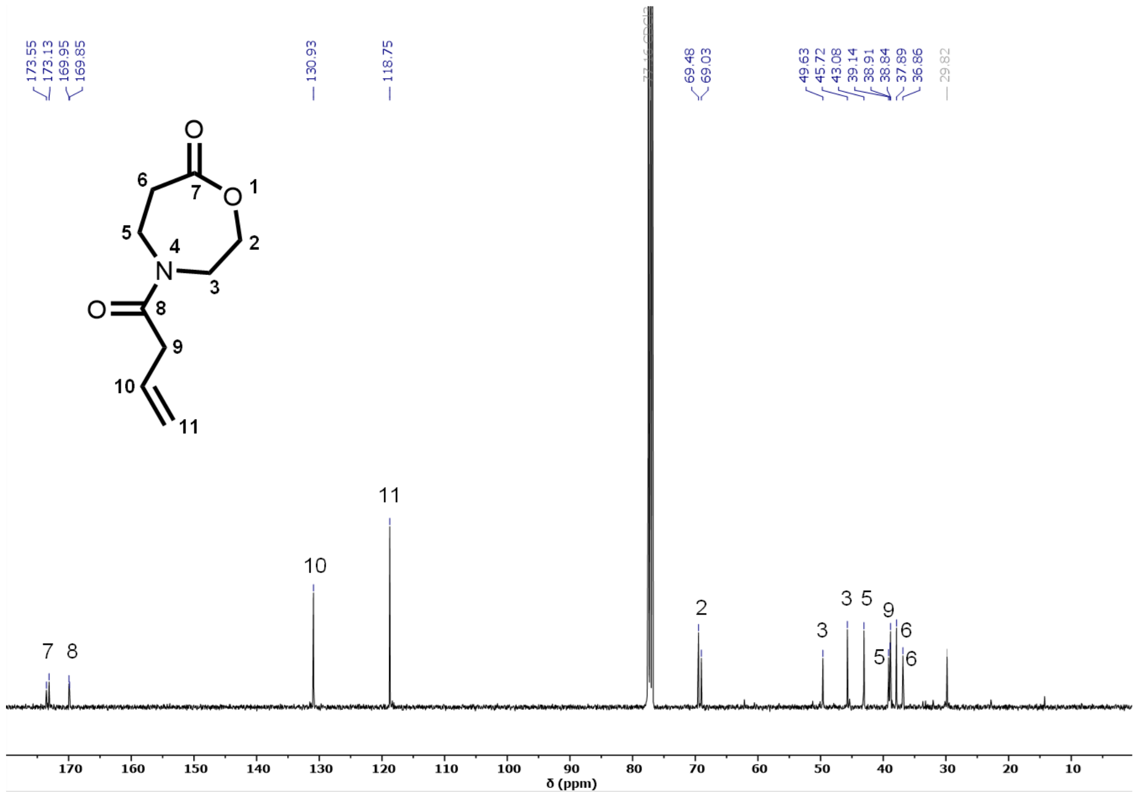

Fig. S33 ^{13}C NMR spectrum of 4-(But-3-enoyl)-1,4-oxazepan-7-one (**OxP$_{Propylene}$**) (400 MHz, CDCl$_3$).

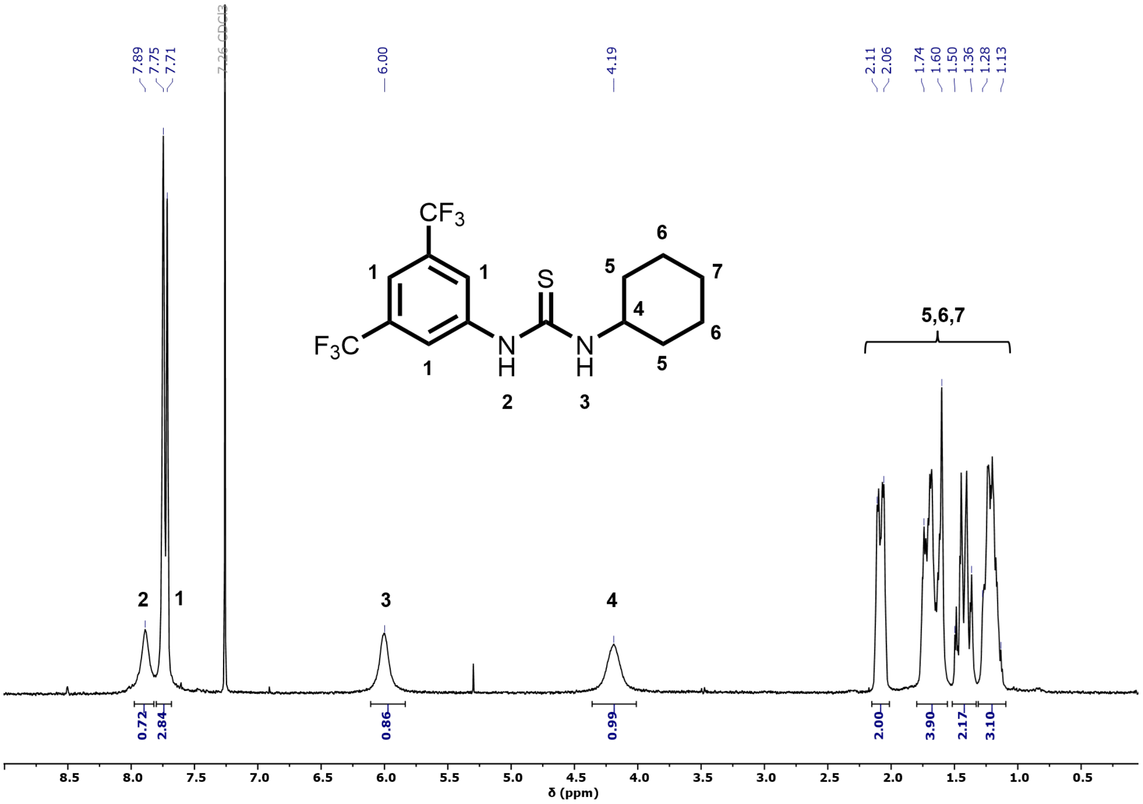

Fig. S34 ^{1}H NMR spectrum of 1-[3,5-bis(trifluoromethyl)phenyl]-3-cyclohexyl thiourea (**TU**) (400 MHz, CDCl$_3$).

6. NMR Data of OxP homopolymers

Poly(4-acetyl-1,4-oxazepan-7-one) (P(OxP$_{Me}$))

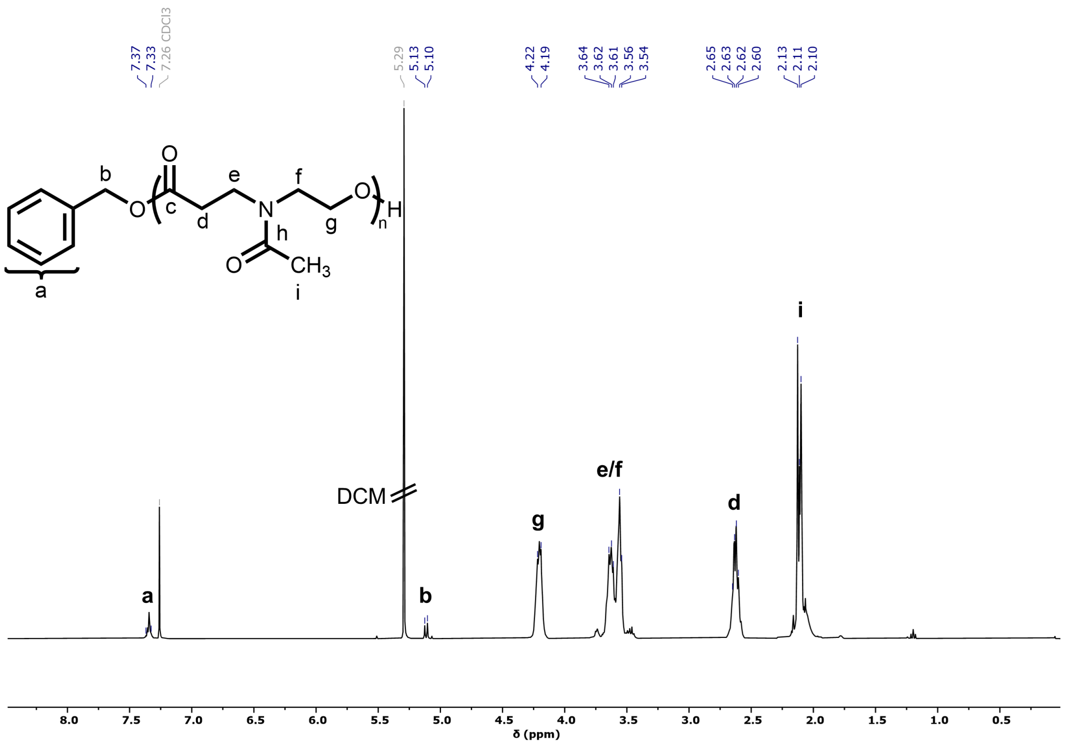

Fig. S35 ^{1}H NMR spectrum of **P(OxP$_{Me}$)** (400 MHz, C*D*Cl$_3$).

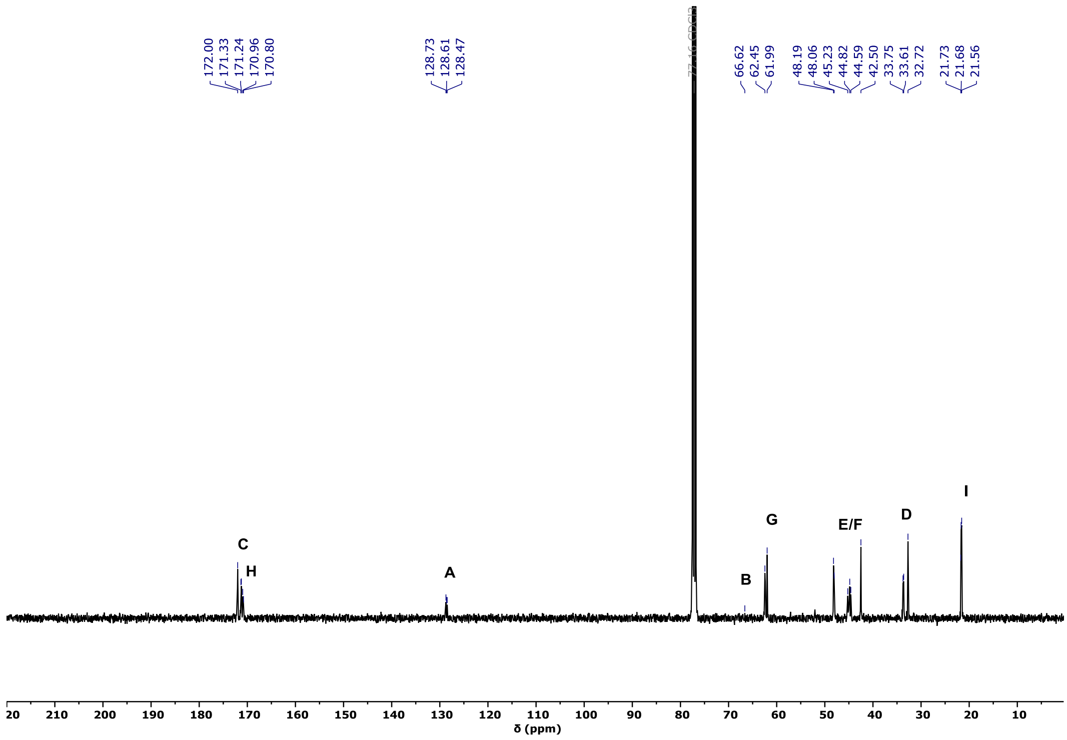

Fig. S36 ^{13}C NMR spectrum of **P(OxP$_{Me}$)** (400 MHz, C*D*Cl$_3$).

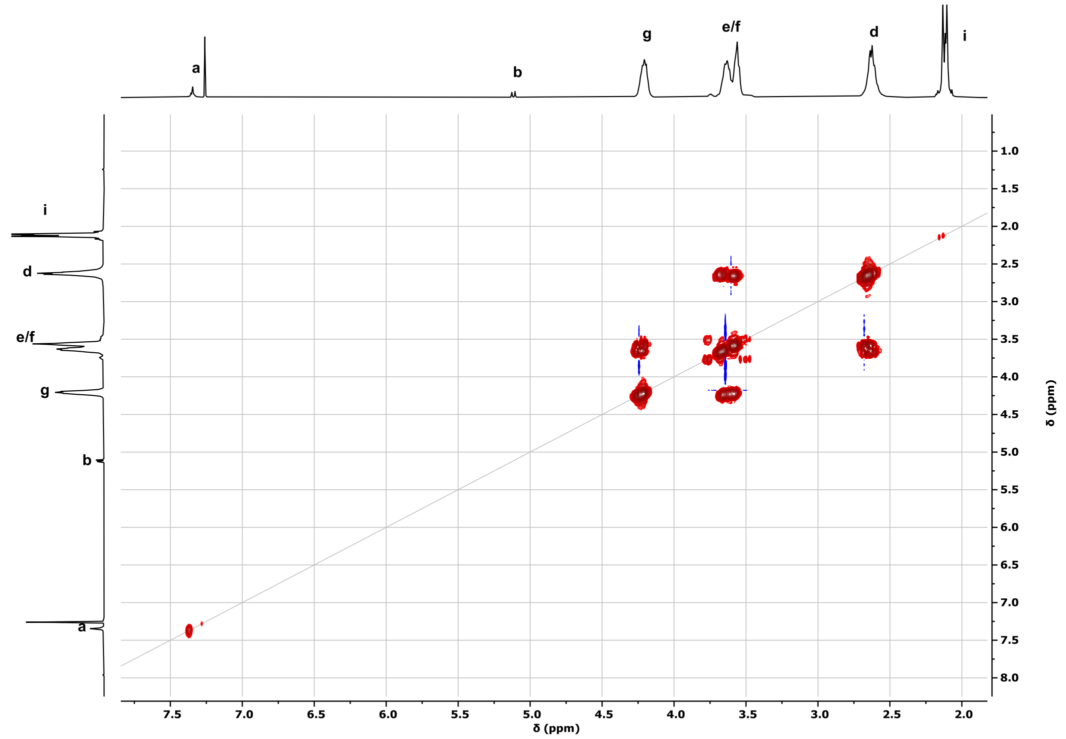

Fig. S37 COSY NMR spectrum of **P(OxP$_{Me}$)** (400 MHz, C*D*Cl$_3$).

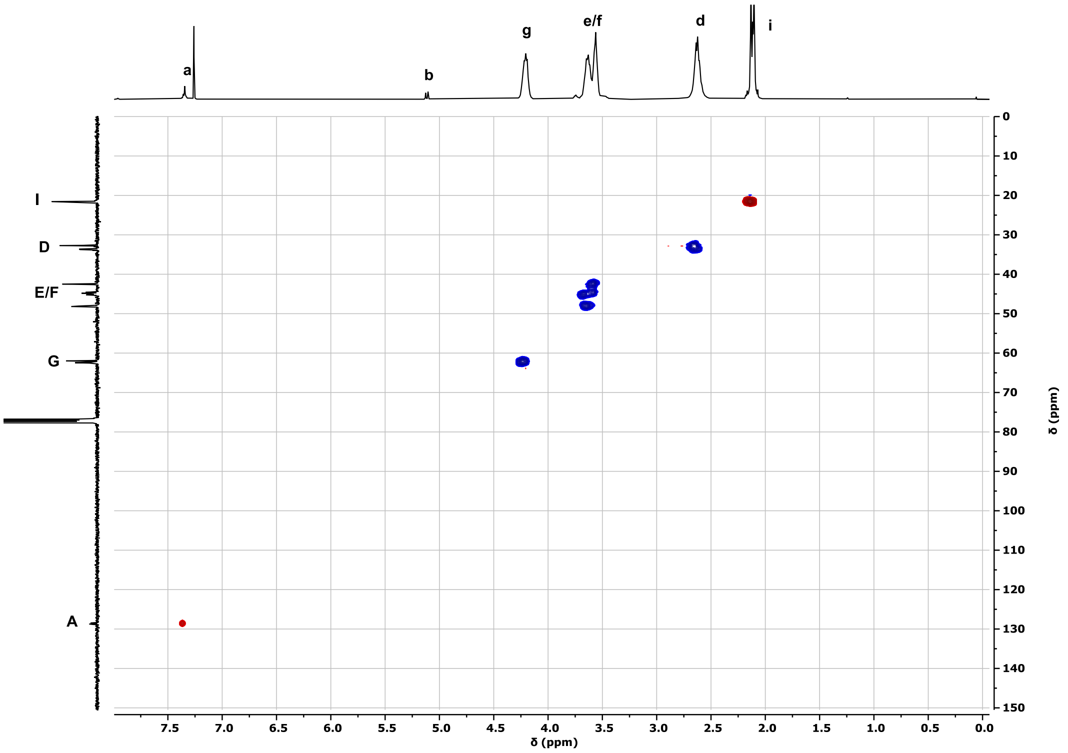

Fig. S38 HSQC NMR spectrum of **P(OxP$_{Me}$)** (400 MHz, C*D*Cl$_3$).

Poly(4-pentanoyl-1,4-oxazepan-7-one) (P(OxP$_{But}$))

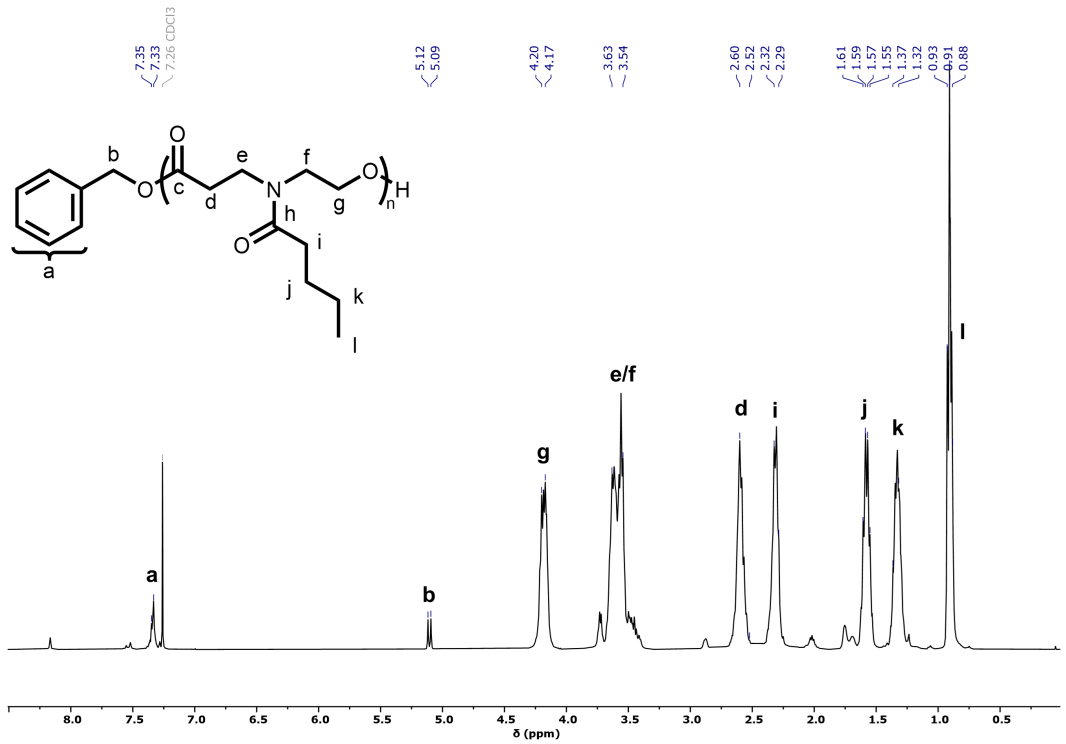

Fig. S39 ^{1}H NMR spectrum of **P(OxP$_{But}$)** (400 MHz, C*D*Cl$_3$).

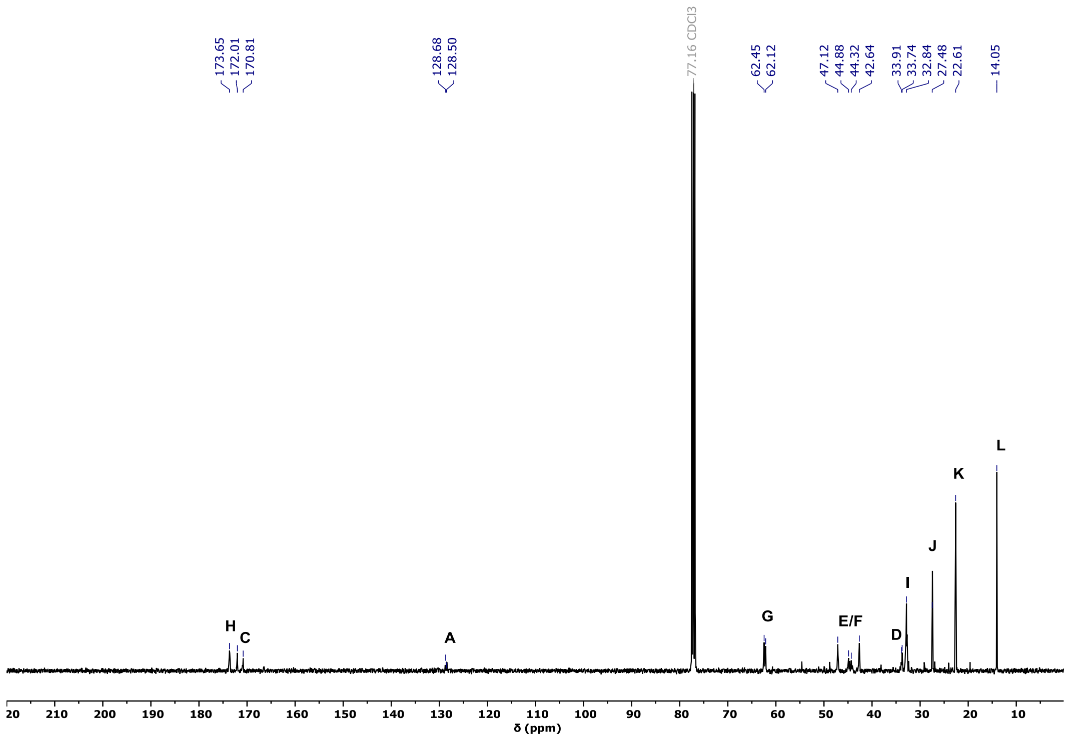

Fig. S40 ^{13}C NMR spectrum of **P(OxP$_{But}$)** (400 MHz, C*D*Cl$_3$).

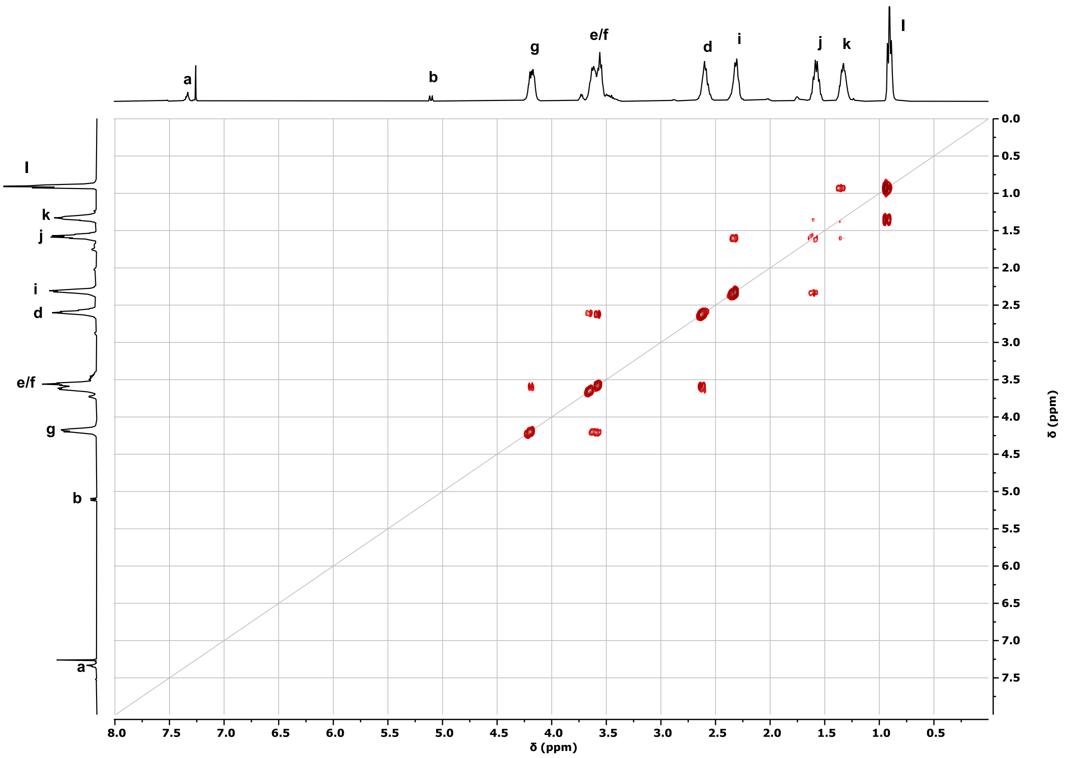

Fig. S41 COSY NMR spectrum of **P(OxP$_{But}$)** (400 MHz, *C*DCl$_3$).

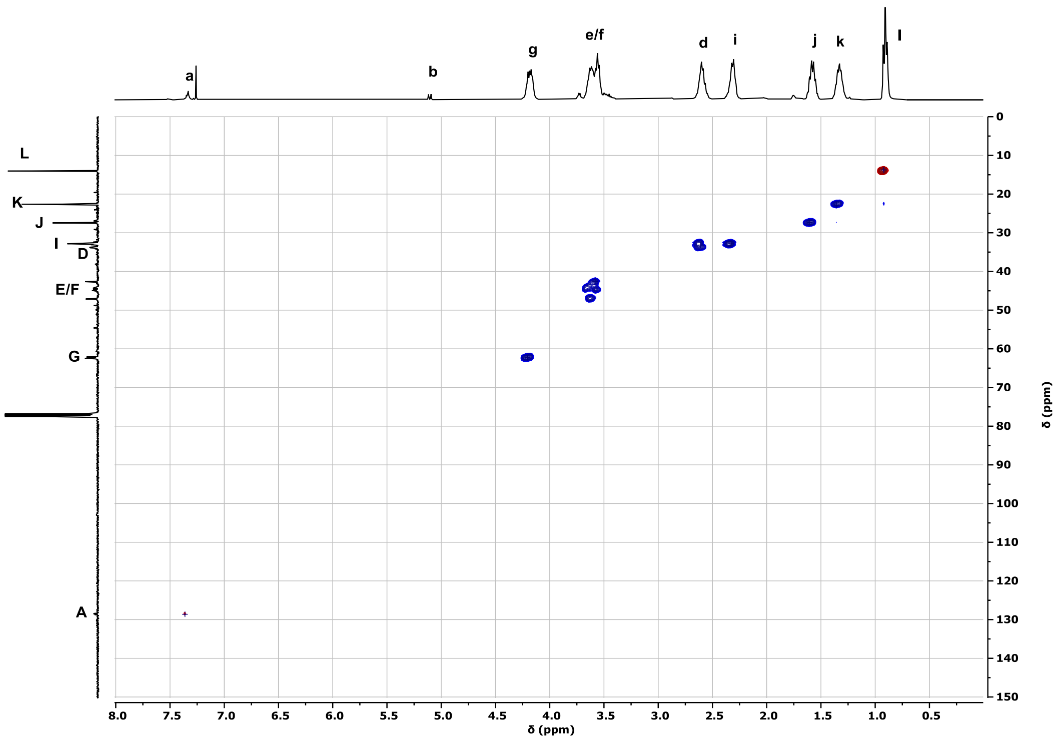

Fig. S42 HSQC NMR spectrum of **P(OxP$_{But}$)** (400 MHz, *C*DCl$_3$).

Poly(4-(3-methylthio)propanoyl-1,4-oxazepan-7-one) (P(OxP$_{EtSMe}$)

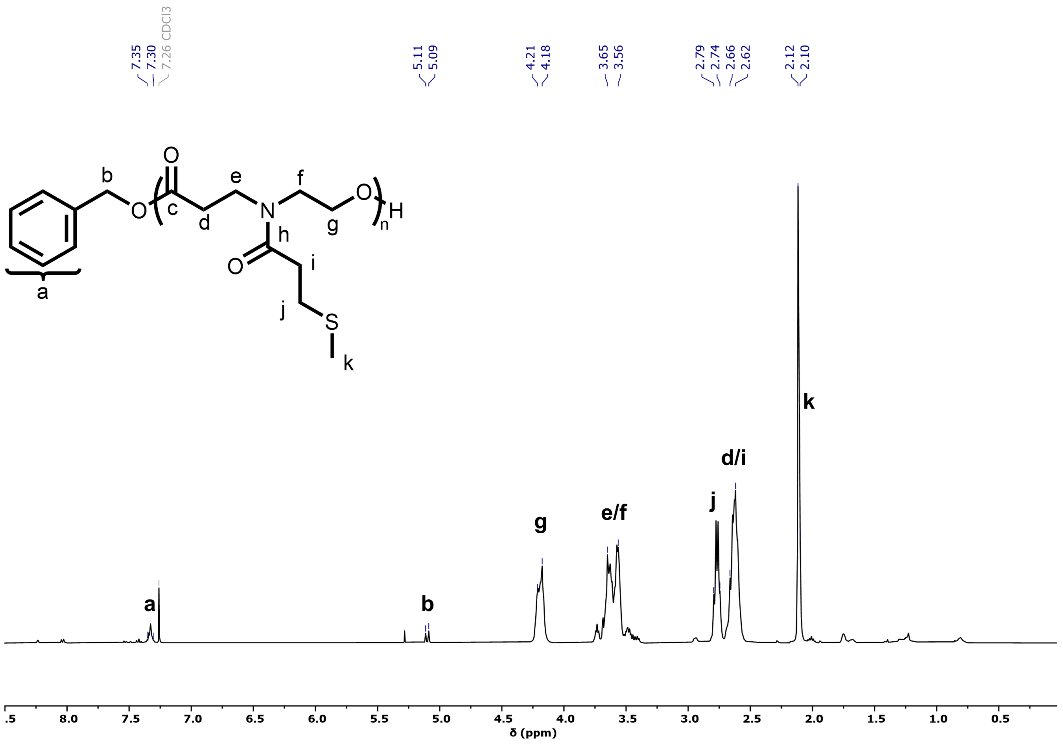

Fig. S43 ^{1}H NMR spectrum of **P(OxP$_{EtSMe}$)** (400 MHz, C*D*Cl$_3$).

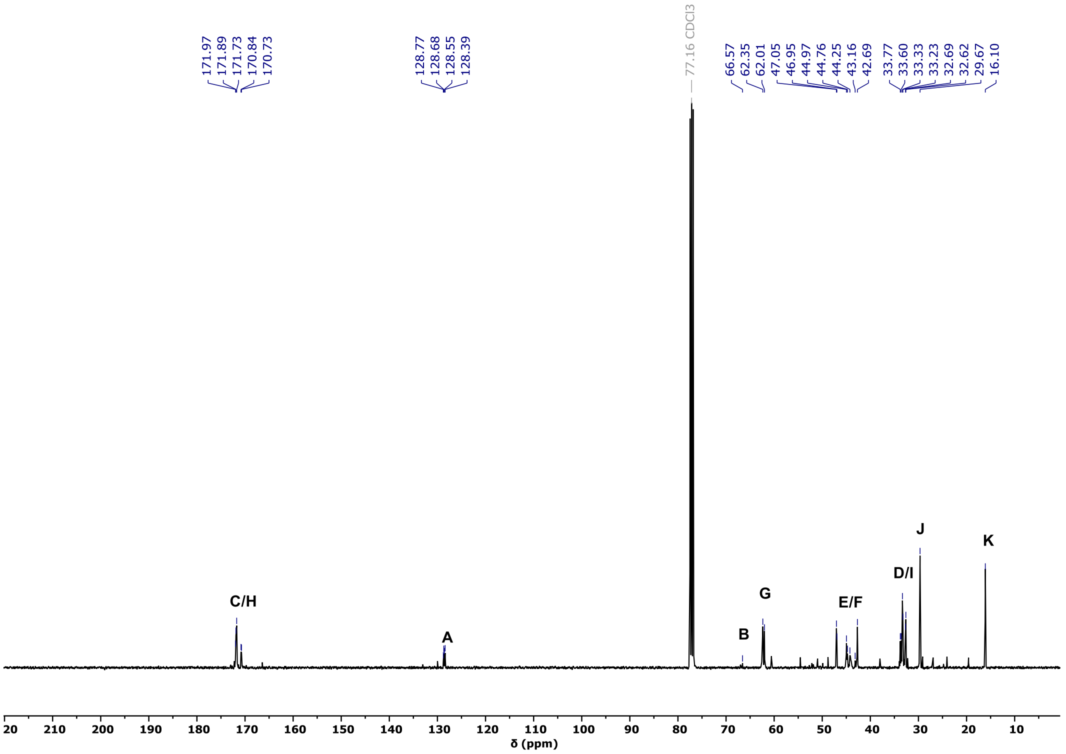

Fig. S44 ^{13}C NMR spectrum of **P(OxP$_{EtSMe}$)** (400 MHz, C*D*Cl$_3$).

Fig. S45 COSY NMR spectrum of **P(OxP$_{EtSMe}$)** (400 MHz, C*D*Cl$_3$).

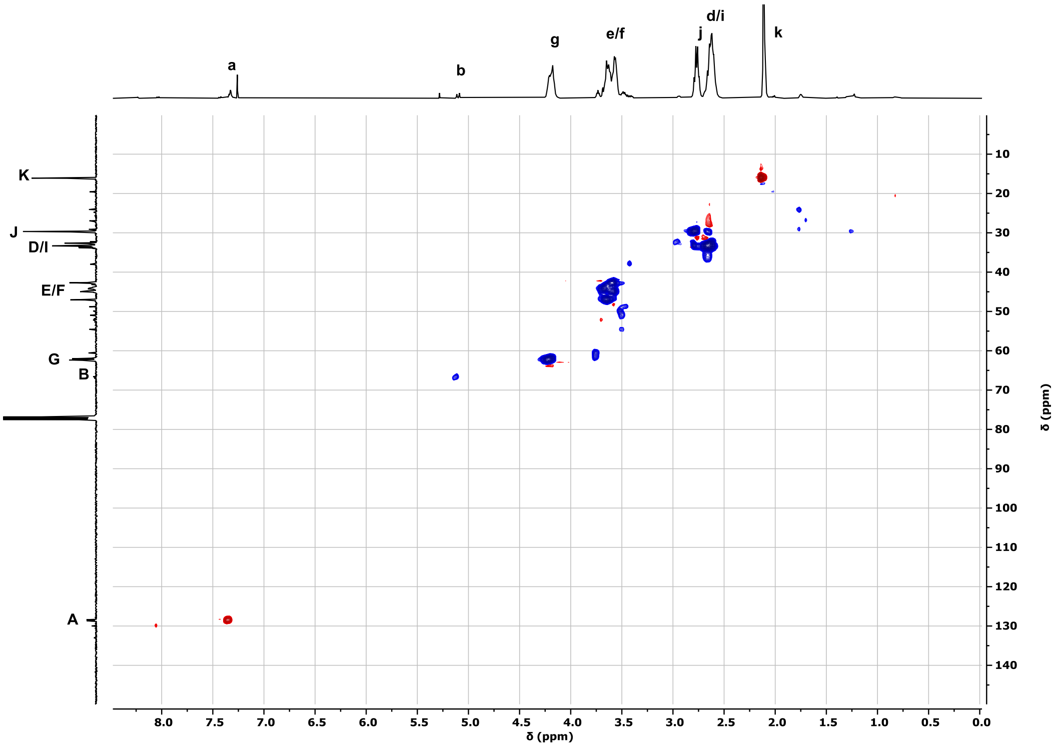

Fig. S46 HSQC NMR spectrum of **P(OxP$_{EtSMe}$)** (400 MHz, C*D*Cl$_3$).

Poly(4-(but-3-enoyl)-1,4-oxazepan-7-one) (P(OxP$_{Propylene}$))

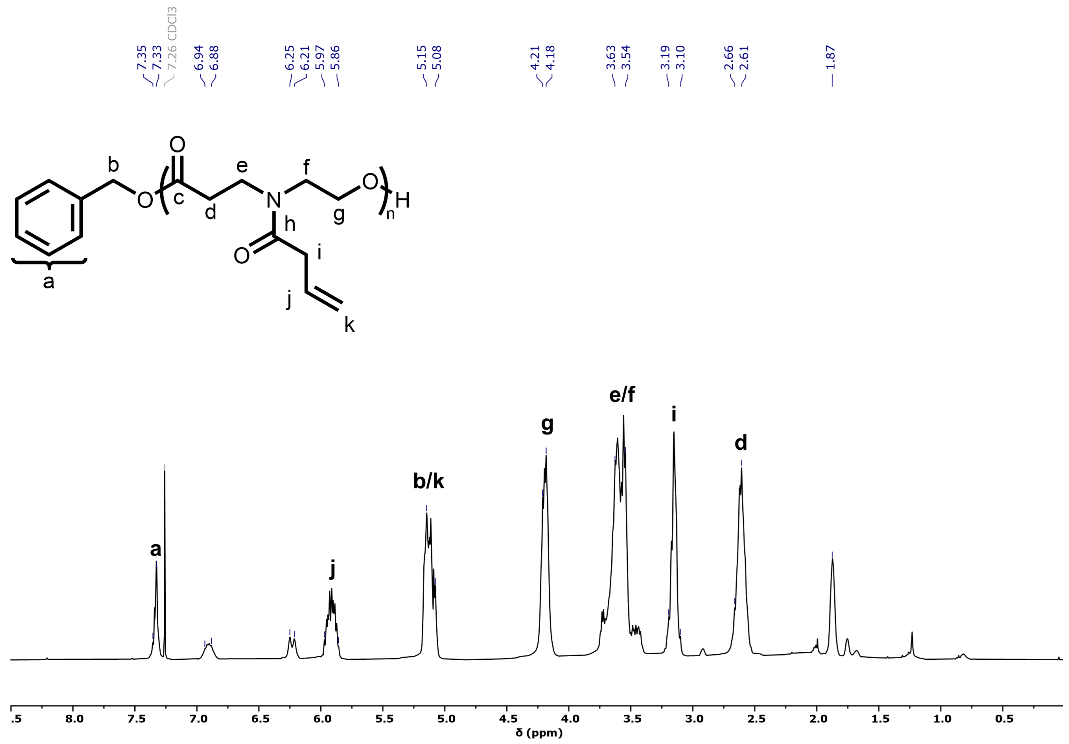

Fig. S47 ^{1}H NMR spectrum of **P(OxP$_{Propylene}$)** (400 MHz, C*D*Cl$_3$).

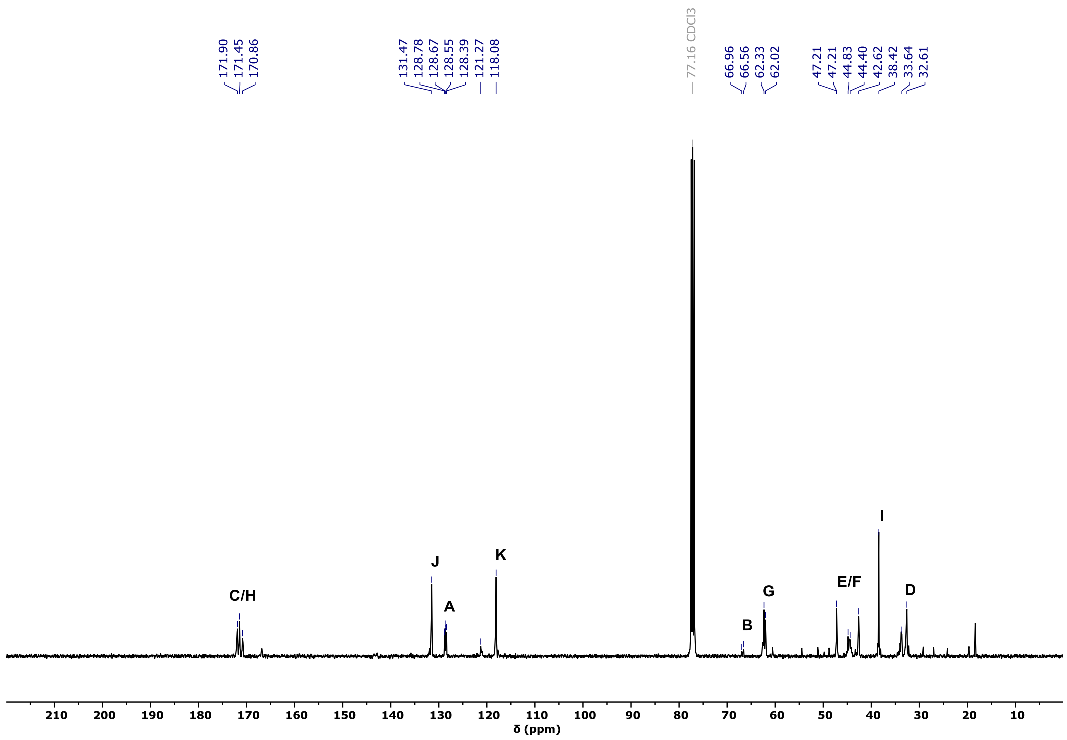

Fig. S48 ^{13}C NMR spectrum of **P(OxP$_{Propylene}$)** (400 MHz, C*D*Cl$_3$).

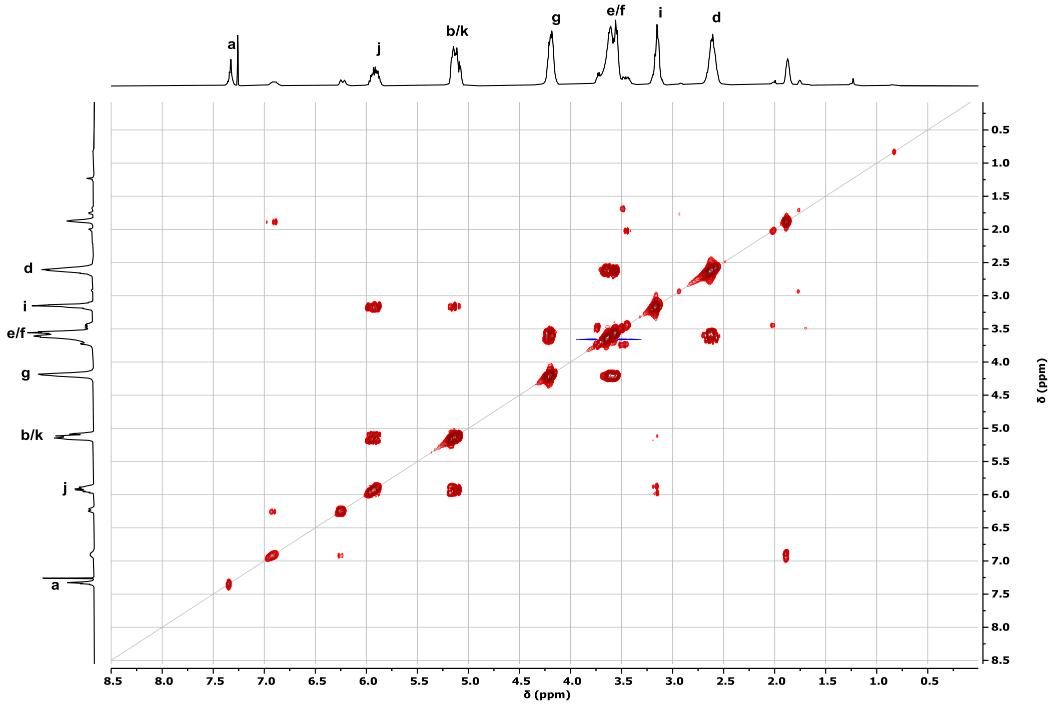

Fig. S49 COSY NMR spectrum of **P(OxP$_{\mathbf{Propylene}}$)** (400 MHz, C*D*Cl$_3$).

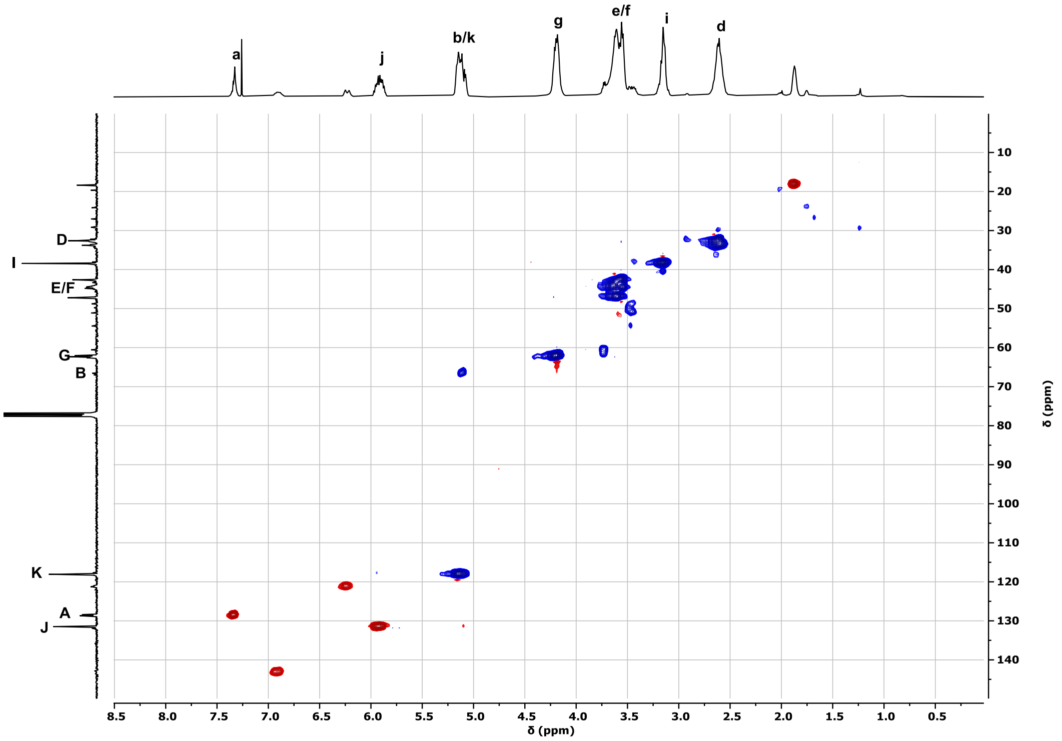

Fig. S50 HSQC NMR spectrum of **P(OxP$_{\mathbf{Propylene}}$)** (400 MHz, C*D*Cl$_3$).

7. Supplementary Data for P(OxP$_{Me}$)$_{25}$-*b*-P(OxP$_{Boc}$)$_{10}$ Block Copolymer Synthesis

General procedure for the synthesis of P(OxP$_{Me}$)$_{25}$-*b*-P(OxP$_{Boc}$)$_{10}$ block copolymer

In an argon-filled glovebox DBU (15 mg, 15 µL, 0.10 mmol, 3.0 eq.), TU (37 mg, 0.10 mmol, 3.0 eq.) and BnOH (4 mg, 3 µL, 0.04 mmol, 1.0 eq.) were dissolved in DCM (0.30 mL). In a different flask, OxP$_{Me}$ (134 mg, 0.85 mmol, 25.4 eq.) was dissolved in DCM (0.30 mL). The amount of solvent was determined to have a final monomer concentration $[M]_0$ = 1 M. After 5 min of stirring, the catalyst/initiator mixture was transferred to the monomer solution to start the polymerization. After 90 min and complete consumption of the first monomer, OxP$_{Boc}$ (75 mg, 0.35 mmol, 10.4 eq.) in DCM (0.24 mL) was added to the reaction flask to induce the polymerization of the second block. After 90 min, the polymerization was quenched by adding benzoic acid. The crude product was precipitated twice in cold Et_2O and dried under reduced pressure.

Characterization data of P(OxP$_{Me}$)$_{25}$-b-P(OxP$_{Boc}$)$_{10}$ block copolymer

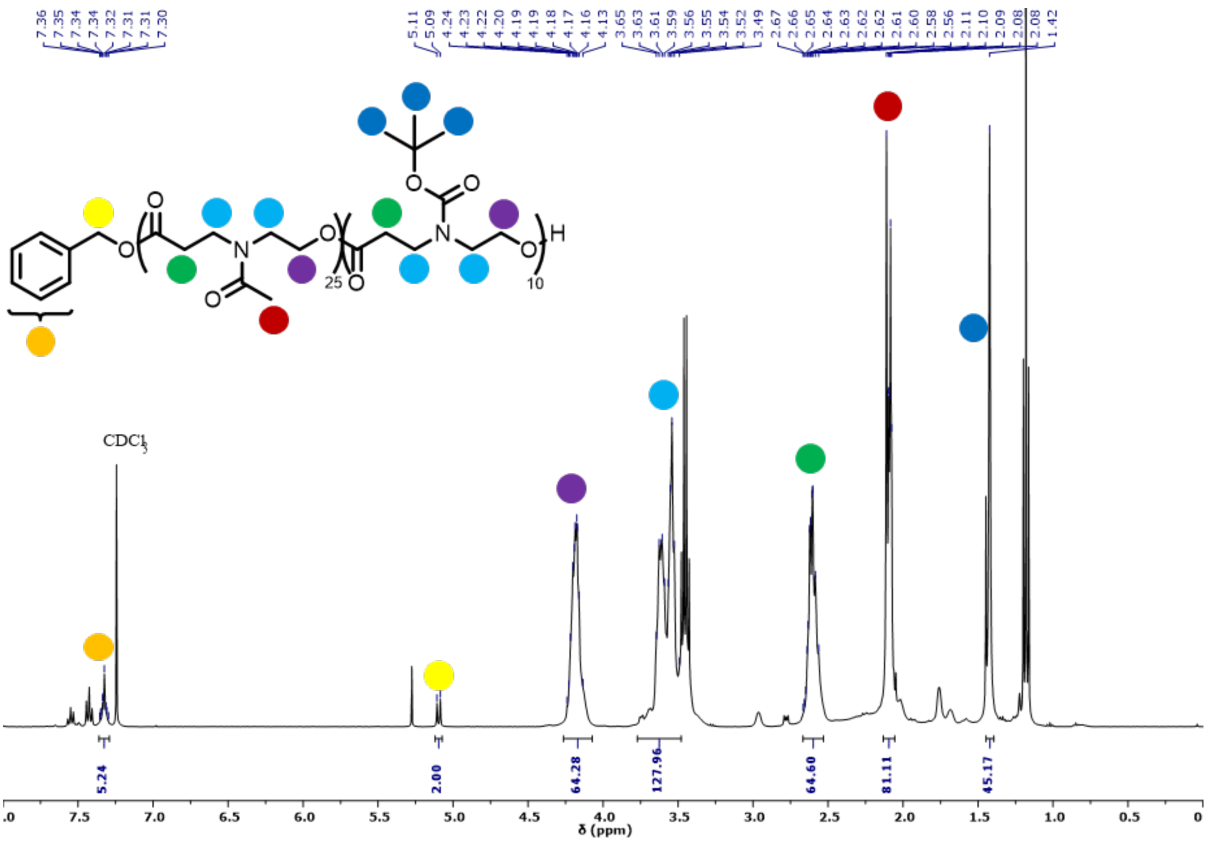

Fig. S51 ^{1}H NMR spectrum of **P(OxP$_{Me}$)$_{25}$-*b*-P(OxP$_{Boc}$)$_{10}$** (400 MHz, C*D*Cl$_3$).

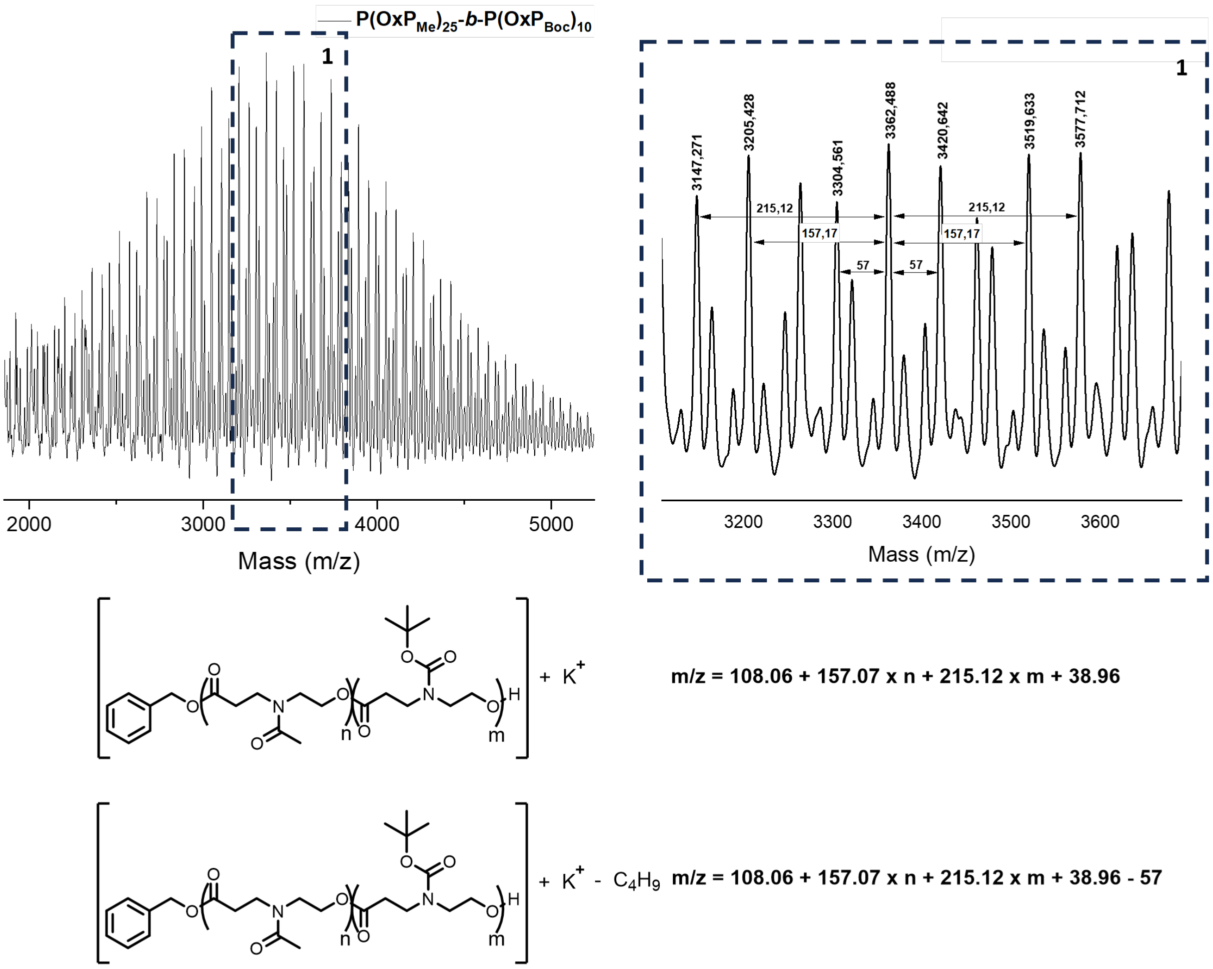

Fig. S52 MALDI-ToF mass spectrum of **P(OxP$_{Me}$)$_{25}$-*b*-P(OxP$_{Boc}$)$_{10}$**.

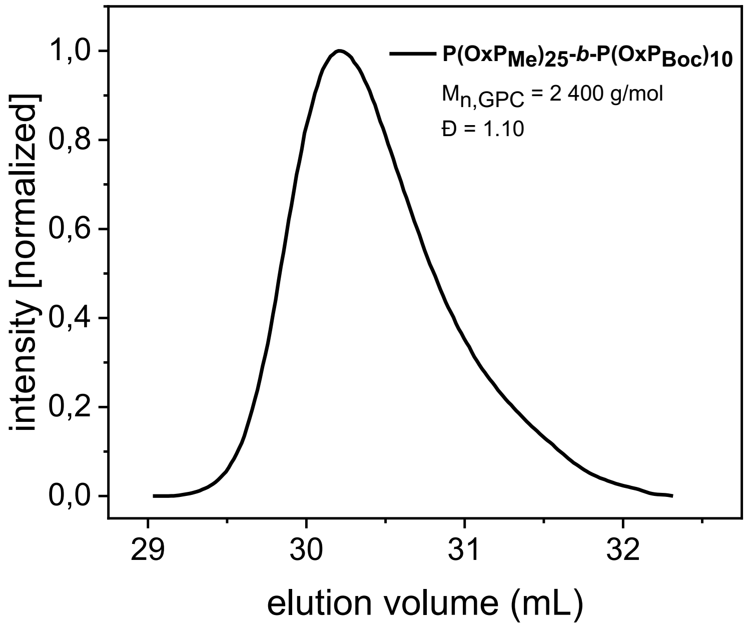

Fig. S53 SEC elution trace of **P(OxP$_{Me}$)$_{25}$-*b*-P(OxP$_{Boc}$)$_{10}$** (RI signal, eluent: THF, 25°C, standard: PMMA).

Table S2 Characterization data of the **P(OxP$_{Me}$)$_{25}$-*b*-P(OxP$_{Boc}$)$_{10}$** block copolymer.

**Polymer**	$\overline{\mathbf{Mn}}$**,theory (g/mol)**	$\overline{\mathbf{Mn}}$**,NMR (g/mol)**	$\overline{\mathbf{Mn}}$**,GPC (g/mol)**	***Đ* (M_w/M_n)**
**P(OxP$_{Me}$)$_{25}$-*b*-P(OxP$_{Boc}$)$_{10}$**	6 200	5 400	2 400	1.10
